# One Health approach probes zoonotic non-typhoidal *Salmonella* infections in China: A systematic review and meta-analysis

**DOI:** 10.7189/jogh.14.04256

**Published:** 2024-12-02

**Authors:** Jiaqi Chen, Linlin Huang, Hongli An, Zining Wang, Xiamei Kang, Rui Yin, Chenghao Jia, Xiuyan Jin, Min Yue

**Affiliations:** 1 Key Laboratory of Systems Health Science of Zhejiang Province, School of Life Science, Hangzhou Institute for Advanced Study, Hangzhou; University of Chinese Academy of Sciences, China; 2 Department of Veterinary Medicine, Zhejiang University College of Animal Sciences, Hangzhou, China; 3 Hainan Institute of Zhejiang University, Sanya, China; 4 State Key Laboratory for Diagnosis and Treatment of Infectious Diseases, National Clinical Research Center for Infectious Diseases, National Medical Center for Infectious Diseases, The First Affiliated Hospital, Zhejiang University School of Medicine, Hangzhou, China

## Abstract

**Background:**

Zoonotic infections, particularly those caused by non-typhoidal *Salmonella* (NTS), pose a significant disease burden. However, there is a notable lack of comprehensive and integrated studies employing the One Health approach to address *Salmonella* prevalence. In this study, we aimed to analyse NTS spatiotemporal prevalence, serovar distribution, and antimicrobial resistance (AMR) across China.

**Methods:**

We conducted a systematic review and meta-analysis to understand the dynamics of NTS in a One Health context in China. We searched the CNKI, Wanfang, and PubMed databases for Chinese and English peer-reviewed articles published before 1 January 2022 dealing with *Salmonella* in the context of China. We examined the dynamic prevalence along the food chain, the risk of dominant serovars and the carriers’ regional contribution by principal component analysis, and the AMR burden before and after the ban on using antimicrobials as feed additives across five decades. We used the inverse variance index as an indicator of the inconsistency across studies, and we adopted the restricted maximum likelihood model due to high heterogeneity for analysis with a 95% confidence level for the pooled prevalence estimate.

**Results:**

Based on 562 retrieved high-quality studies during 1967–2021, representing 5 052 496 samples overall and 80 536 positive samples for NTS isolates, the overall average prevalence was 7.35% (95% confidence interval (CI) = 0.069–0.087), which was regionally relatively higher in northern China (8.19%; 95% CI = 0.078–0.117) than in southern China (6.94%; 95% CI = 0.067–0.088). Poultry was the primary vehicle for serovars Enteritidis and Indiana (especially in the north), while swine and ruminants for Typhimurium and Derby were the first to highlight the regional livestock contribution to serovar prevalence. The overall AMR rate was 73.63% (95% CI = 0.68–0.99), decreasing after the ban on excessive use of feed-based antibiotics in livestock since 2020, with a relatively low resistance towards front-line and last-resort drugs.

**Conclusions:**

Our study emphasises the importance of adopting a One Health framework to better understand the zoonotic nature of human NTS and highlights the dominant serovars on food contamination and human infection. The similarity in AMR patterns between poultry and human isolates further emphasises the integrated approach for evaluating disease burden and implementing targeted interventions.

*Salmonella* is a zoonotic agent, primarily transmitted through food, causing approximately 115 million cases and 370 000 deaths worldwide each year [1], ranking as the leading cause in the US and China, and second among foodborne bacterial diseases in the European Union (EU) [2–4]. Generally, two distinct clinical disorders can be caused by higher primates-restricted typhoidal *Salmonella* and non-typhoidal *Salmonella* (NTS) with a broad vertebrate host range [5]. Serovars other than Typhi, Paratyphi, and Sendai can lead to non-typhoidal salmonellosis, exhibiting self-limiting diarrhoea yet occasionally systematic clinical syndromes, resulting in bacteremia, pneumonia, and splenomegaly, with a fatality rate of 20–25% in under-developed regions, especially for the immunocompromised, young, and elderly [6,7]. However, the disease burden for NTS infection in many developing countries, including China, remains unaddressed.

Intensification of farming, industrialisation, and globalisation, while essential to meet the growing protein needs of humankind, has also accelerated the dissemination of foodborne pathogens, including *Salmonella*. This underscores the crucial need for an improved investigation of food animals (FA) as the primary reservoir [8,9]. A previous regional surveillance data in China detected a carriage rate of 10.5% in chicken and 24.4% in swine [10], 20.0% with food contamination [11], while another study estimated the disease burden at 245 per 100 000 persons from July 2010 to July 2011 [12]. However, most epidemiological studies were limited by specific spatiotemporal data sets [5,12,13], and the research on the prevalence and risk in the One Health context is largely lacking.

The prevalence of *Salmonella* and the dominant serovars in FA and derived foods is dynamic and varies across regions and over time. Understanding the historical context is crucial here, as *S. *Enteritidis would fill the ecological niche left by *S. *Gallinarum following its eradication in the poultry industries of the EU and USA during the 1970s. This shift led to *S. *Enteritidis having the highest carriage rate in poultry (~10%) and the clinical dominance [2,14,15], highlighting the need for regional-specific and temporal control programmes [16].

Antimicrobial resistance (AMR) is a global threat, and the need for updated knowledge on AMR preference and transmission pathways is more pressing than ever for shaping effective clinical practices. In 2019, AMR pathogens, including *Salmonella*, caused an estimated 1.27 million deaths globally, and this number is projected to rise to 10 million by 2050 [17]. Alarmingly, over 70% of antibiotics produced are being used for livestock rearing [18,19]. A significant portion of this usage occurs in China, a country that has been the top consumer of antibiotics during FA production since 2010 [20]. This high consumption in China could lead to the selection of multidrug-resistant clones in farm animals and accelerate the spread of AMR genes, such as those encoding extended-spectrum beta-lactamase along the food chain to other commonly examined bacteria [21]. However, our understanding of food-chain-based AMR *Salmonella* transmission is still limited.

By conducting an evidence-based systematic review and meta-analysis of studies published from 1967 to 2021, we intended to explore the dynamic nature of NTS, the predominant serovars, and the crucial role of animal reservoirs in the prevalence of these serovars in China. This could enhance our understanding of the NTS burden in China and provide baseline knowledge for establishing effective control measures against these commonly detected zoonotic transmissions.

## METHODS

### Search strategy

We searched CNKI, Wanfang, and PubMed using a bilingual search strategy based on keywords related to ‘*Salmonella*’ and ‘China’ to retrieve articles published before 1 January 2022. We excluded articles that were not in English or Chinese. We reported our findings according to PRISMA guidelines [22].

### Inclusion and exclusion criteria

**Figure 1:**
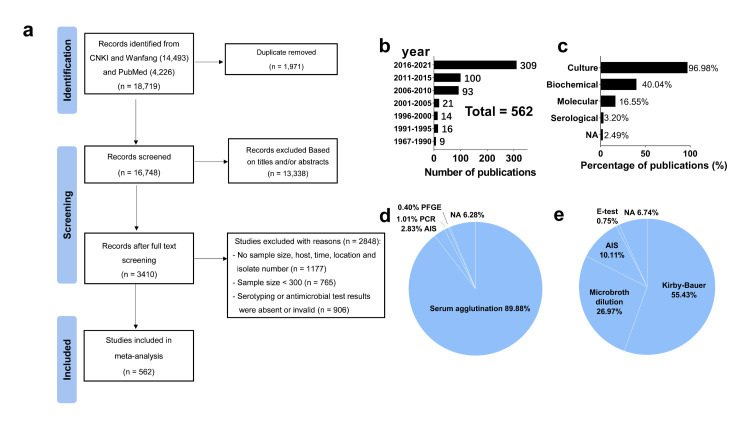
Summary of study processes and data. The black bars indicate the sample size on the left axis and the red lines indicate prevalence on the right axis. Prevalence was calculated per the formula prevalence (%) = N (positive sample)/(N (total sample size) × 100%). **Panel A**. Flow diagram of the literature search. **Panel B**. The publication year of selected studies. **Panel C.** Percentage of methods in articles used for bacterial isolate identification. **Panel D.** Typing method. **Panel E.** Method for Antimicrobial susceptibility test. AIS – automatic identification systems, PCR – polymerase chain reaction, PFGE – pulsed-field gel electrophoresis.

We imported the retrieved studies in Endnote, version 19 (Clarivate Analytics, Philadelphia, USA) for deduplication and title, abstract, and full-text screening. We excluded articles concerned with invasive *Salmonella *infections, reporting typhoidal *Salmonella*, mechanism investigation, case reports, editorial, commentaries, and reviews. After full-text reading, we excluded any remaining studies not providing specific information on sample size, host, location, or isolate number; having a sample size of less than 300, which could lead to potential bias or incorrect calculations; or lacking serotyping or antimicrobial test results, or presenting invalid results (**Figure 1**, Panel A).

### Data extraction

After the exclusion and eligibility assessment, we extracted their data in pre-designed extraction tables in Microsoft Excel 2016 (Microsoft Inc, Seattle WA, USA), including information on publication title, literature reference, publication year, sampling information (sampling year, province or municipal city, host), the methods of isolate identification, serotyping and antimicrobial susceptibility tests, the number of isolates of specific serovars, and the number of AMR strains (Tables S1–4 in the **Online Supplementary Document**). We did not analyse the sequence type (ST) or subtypes (i.e. a monophasic variant of *S. *Typhimurium, 1,4,[5],12: i:-) because of the sparse data. Three independent authors (JC, LH, HA) performed the data extraction and selection.

### Data quality evaluation and analysis

We used the inverse variance index (*I^2^*) to assess the level of inconsistency across studies, with values of 25%, 50%, and 75% considered as cutoff points for low, moderate, and high heterogeneity, respectively. We adopted a restricted maximum likelihood model due to high heterogeneity for analysis with a 95% confidence level for the pooled prevalence estimate using Open Meta-Analyst [23–25]. We performed the plotting using GraphPad Prism software, version 10.1.2 (GraphPad Software Inc., California, US). We presented the situation on serovars prevalence in proportion instead of percentage for clarity.

### Principal component analysis

To understand the correlations among the top 15 serovars dissemination, hosts, and regional factors, we performed a principal component analysis (PCA), following a previously published protocol [16]. We projected the principal components (factors) based on the correlation matrix. For better presentation, we presented the loadings and PC scores separately instead of a combined biplot. We determined the contribution of host or regional factors to serovar dissemination by the distance of clusters in two projections [26]. We assessed the quality of the data by Kaiser-Meyer-Olkin and Bartlett's Test using SPSS, version 27 (IBM Corp., Armonk, New York, USA). We performed the plotting using GraphPad Prism software, version 10.1.2 (GraphPad Software, Inc., US). We determined the regional north-south division according to ‘Qinling Mountains and Huaihe River’ as described in previous studies [27] (Table S5 in the **Online Supplementary Document**). We referred to the government’s official website for the law and legislation [28]. Abbreviations of antimicrobials investigated are listed in Table S6 in the **Online Supplementary Document**.

## RESULTS

During the initial selection, we retrieved 14 493 Chinese- and 4226 English-language studies from three databases before 1 January 2022, from which we excluded 1971 duplicates and 13 338 unrelated studies based on titles and abstracts (**Figure 1**, Panel A). For the remaining 3410 studies, we excluded those with insufficient information (n = 1177), those with a sample size under 300 (n = 765), and those with invalid serotyping or AMR test results (n = 906). We retained 562 studies for analysis, representing 5 052 496 total samples and 80 536 positive samples for *Salmonella* isolates (Tables S1 and S2 in the **Online Supplementary Document**). The sampling origins were mostly animal-borne food-chained based and were summarised into FA, including poultry, swine and ruminants, food, and humans for further interpretation. The number of published studies was growing due to the increasing attention paid to the foodborne and zoonotic nature of NTS (**Figure 1**, Panel B). Traditional culture (96.98%) was most widely used for isolates’ identification (**Figure 1**, Panel C). Serum agglutination (89.88%) and Kirby-Bauer (55.34%) were most frequently adopted for serotyping and antimicrobial susceptibility tests (**Figure 1**, Panels D and E).

### Nationwide spatiotemporal prevalence of non-typhoidal *Salmonella*

**Figure 2:**
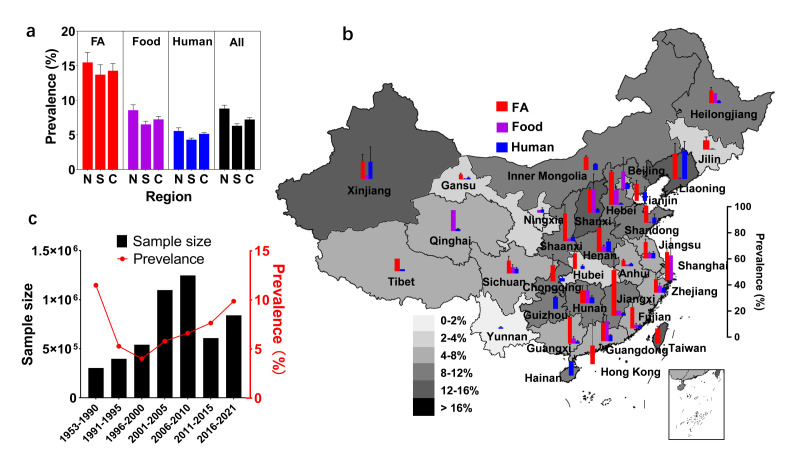
Spatiotemporal prevalence from different sources. **Panel A.** Prevalence of NTS in food animals (FA), food (**F**), and humans (**H**) across regions. **Panel B.** Prevalence of NTS in food animals (FA), food (**F**), and humans (**H**) across provinces. **Panel C.** Temporal changes in overall sample size and prevalence. C – China, N – northern China, NTS – non-typhoidal salmonella, S – southern China.

The overall prevalence was found to be higher in northern China (8.19%; 95% confidence interval (CI) = 0.078–0.117) than in southern China (6.94%; 95% CI = 0.067–0.088), although this difference was not statistically significant. This regional difference persisted regardless of origins (**Figure 2**, Panels A and B). The FA prevalence is higher than in food and humans, indicating the foodborne transmission route of NTS, and most provincial and municipal cities included data from various origins (**Figure 2**, Panels A and B; Figure S1 in the **Online Supplementary Document**). Due to the growing attention on food safety, the sampling size increased until 2010, after which it decreased (**Figure 2**, Panel C). The reason was speculated to be that more large-scale epidemiological investigations were dedicated to chemical toxins after the ‘Melamine Scandal’ of milk in 2008, and numerous emerging diseases such as COVID-19. The overall national disease incidence decreased before 2001, due to the relieved burden in FA, where the poultry carriage rate declined to 10% (95% CI = 0.089–0.191) during 2001–05 (Figure S1, Panels A and D in the **Online Supplementary Document**) owing to the inclusion of non-typhoidal salmonellosis into the mitigation program. Additionally, swine and ruminants witnessed a downward trend to 10% (95% CI = 0.091–0.182) due to benefits from veterinary biologics development and the legislation on epidemics in FA since 1997 (Figure S1, Panels E and F in the **Online Supplementary Document**). Additionally, the food contamination rate was controlled below 8.0% (95% CI = 0.058–0.110) after the food administration law concerning NTS in 2002 (Figure S1, Panel B in the **Online Supplementary Document**). The overall prevalence rose to 9.84% (95% CI = 0.083–0.123) during 2016–21 (**Figure 2**, Panel C), with a FA carriage rate of 14.33% (95% CI = 0.128–0.216), a food contamination rate of 5.74% (95% CI = 0.002–0.215), and a human disease incidence of 2.44% (95% CI = 0.008–0.071) (Figure S1, Panels A–C in the **Online Supplementary Document**).

### Dynamic predominant serovars among hosts from 1953 to 2021

**Figure 3:**
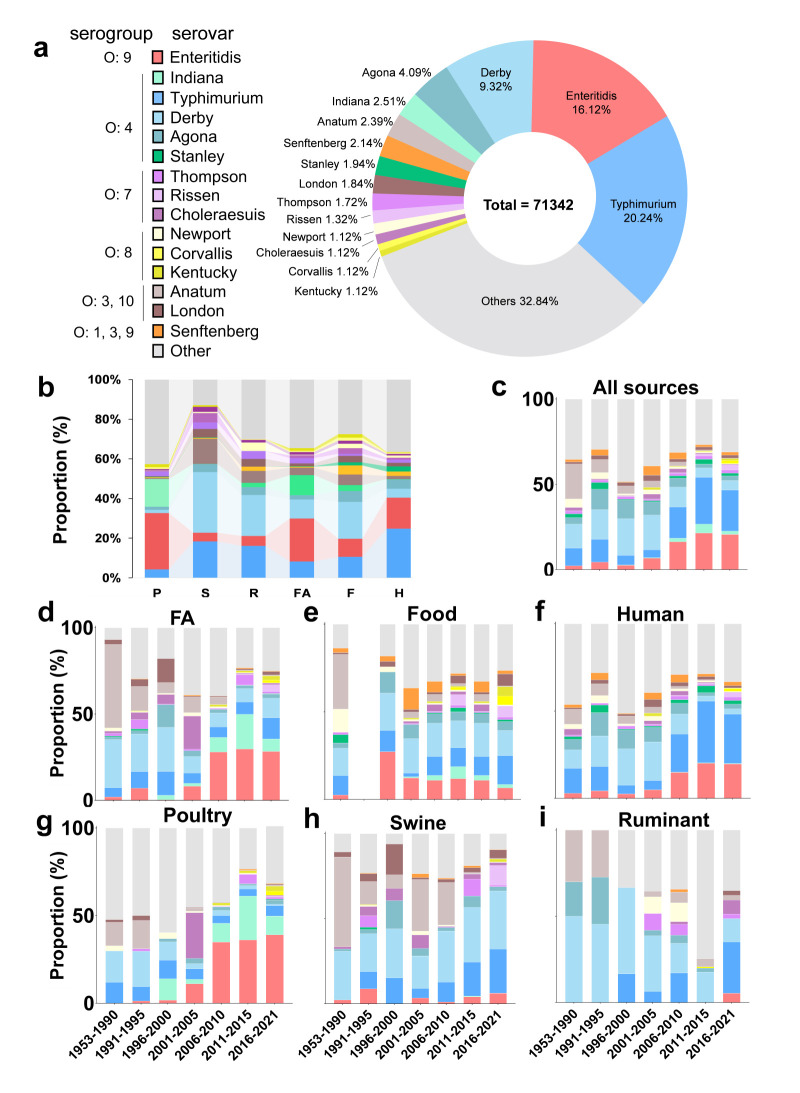
The dynamic pattern of the top fifteen serovars from 1953 to 2021. Total strands for the number of isolates serotyped were calculated per the formula proportion (%) = N (strains of serovars X)/(N(total of strains serotyped) × 100%). **Panel A**. Top fifteen serovars and proportion. **Panel B.** The proportion of the top fifteen serovars in poultry, swine, and ruminants, collectively referred to as food animals, food, and humans. **Panel C.** Dynamics of the top fifteen serovars from 1953 to 2021 from all origins. **Panels D–I.** Dynamics of the top fifteen serovars from 1953 to 2021 from different hosts. F – food, FA – food animals, H – human, P – poultry, R – ruminants, S – swine.

Based on collected data (Table S3 in the **Online Supplementary Document**), we analysed the top fifteen serovars out of 267 serovars in China, accounting for 67.16% of reported incidences, particularly in swine (86.73%) (**Figure 3**, Panels A and B). The most prevalent serovars were *S. *Typhimurium, *S. *Enteritidis and *S. *Derby in most origins (Figure S2, Panels A–C in the **Online Supplementary Document**). Predominant serovars were distributed disproportionally among hosts, where poultry was the most common reservoir for *S. *Enteritidis and *S. *Indiana, in contrast with swine and ruminants, which mostly hosted *S. *Typhimurium and *S. *Derby (**Figure 3**, Panel B). Most predominant serovars were less important clinically after food processing and cooking, while the high food contamination rate of *S. *Senftenberg was noteworthy, indicating a higher tolerance under a range of vitro conditions.

The temporal dynamics of fifteen predominant serovars showed that *S. *Enteritidis and *S. *Typhimurium increased in prevalence, while *S. *Derby and *S. *Agona decreased (**Figure 3**, Panel C). The temporal prevalence in various hosts was quite dynamic (**Figure 3**, Panels D–I), where data on food during 1991–95 is absent. *Salmonella* carriage in poultry is primarily driven by *S. *Enteritidis and *S. *Indiana, which have replaced *S. *Typhimurium, *S. *Derby, and *S. *Choleraesuis (**Figure 3**, Panel G). This shift has contributed to the high carriage rates of these two serovars in FA (**Figure 3**, Panel D), alongside the rapid growth of the poultry industry compared to other livestock [29]. However, *S. *Typhimurium, serogroup O:7 (*S*. Thompson, *S*. Rissen, and *S*. Choleraesuis) and O:3, 10 (*S*. Anatum and *S*. London) mostly threatened swine and ruminants (**Figure 3**, Panels H and I). The advantage of predominant serovars in FA, such as *S. *Enteritidis, was impaired by other serovars, including *S*. Corvallis and *S*. Agona, in food (**Figure 3**, Panel E). In recent decades, *S. *Typhimurium and *S. *Enteritidis became more clinically important than *S. *Derby and *S*. Agona (**Figure 3**, Panel F). Additionally, *S*. Corvallis emerged as a top serovar both in the food chain (FA and food) and clinically between 2016 and 2021, underscoring the impact of foodborne zoonosis from upstream food chain contamination on human non-typhoidal salmonellosis burden (**Figure 3**, Panels B and F).

### Role of contribution to the prevalence of top fifteen serovars

**Figure 4:**
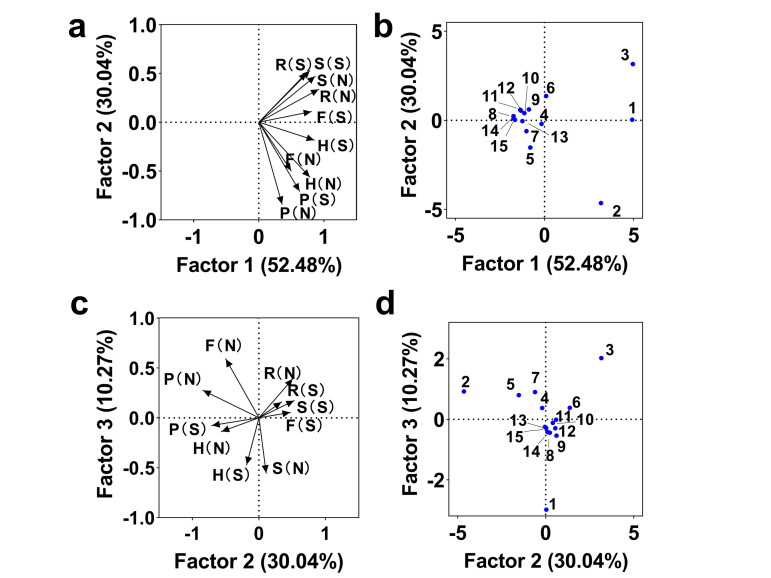
The contribution to the prevalence of the top fifteen serovars among various host groups in northern and southern China using PCA analysis. Numerics 1–15 stand for the top fifteen serovars in order: 1 – Typhimurium, 2 – Enteritidis, 3 – Derby, 4 – Agona, 5 – Indiana, 6 – Anatum, 7 – Senftenberg, 8 – Stanley, 9 – London, 10 – Thompson, 11 – Rissen, 12 – Newport, 13 – Choleraesuis, 14 – Corvallis, and 15 – Kentucky. F – food, H – human, N – northern China, P – poultry, S – southern China, S – swine.

To further clarify the contribution of each host to the top serovar prevalence in northern and southern China, we also applied PCA analysis after assessing the data quality using Kaiser-Meyer-Olkin and Bartlett's Test. The PCA factors 1, 2, and 3 explained 92.79% of the data variance (**Figure 4**, Panels A–D). All hosts were vital for transmitting *S. *Typhimurium, *S. *Derby, and *S. *Enteritidis according to factor 1. Poultry was the critical vehicle in *S. *Enteritidis transmission nationally, while *S. *Indiana carriage was more common in the northern poultry industry, as indicated by factor 2. Factors 2 and 3 suggested swine and ruminants were the primary carriers of *S. *Typhimurium and *S. *Derby, with serovars being more complicated than poultry, especially in northern swine, harbouring *S*. Stanley, *S*. London, *S*. Thompson, and *S*. Newport. Furthermore, in the south, food and humans were closely associated with swine and ruminants, while in the north, the poultry industry had a stronger influence. These patterns were highly associated with north-south differences in livestock industry distribution and diet preference, as shown in the visualised data map on the Chinese National Bureau of Statistics [30]. Additionally, the north-south difference may be attributed to the regional dominance of different subtypes within the same serovar, as recently documented [31].

### Assessing AMR NTS potential along the foodborne chain

**Figure 5:**
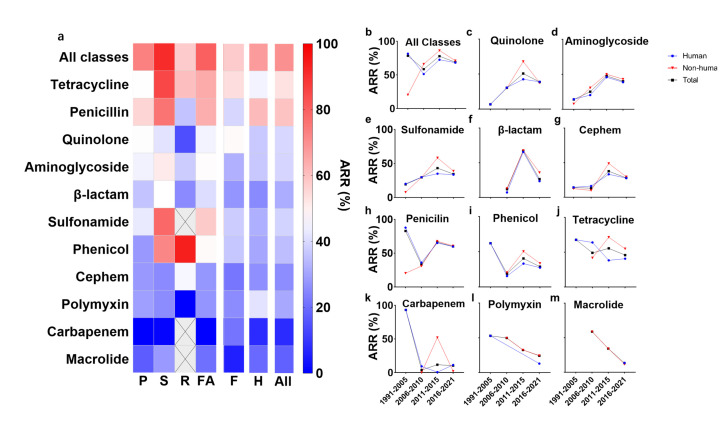
Antimicrobial resistance rate in different hosts and periods. The dark grey wells with a cross indicate that data are not available in this point. **Panel A.** Antimicrobial resistance rate of eleven antimicrobial classes in poultry, swine, ruminant, food animal, food, humans, and all sources. **Panels B–M**. Dynamics of antimicrobial resistance rates from different origins. ALL – all sources, ARR – antimicrobial-resistant rate, F – food, FA – food animals, H – human, P – poultry, R – ruminants, S – swine.

To evaluate the AMR burden of NTS in China, we investigated the resistance rate for 11 antimicrobial classes under surveillance since 1991, before which the survey was rare (Table S4 in the **Online Supplementary Document**). The overall AMR rate was 73.15%, with tetracycline (52.69%), penicillin (59.29%), and quinolone (39.39%) not being recommended for anti-infection therapy. In comparison, carbapenem (7.87%) and macrolide (17.67%) can still be used as last-resort drugs (**Figure 5**, Panel A). The AMR was more severe in FA than in humans, especially for swine, the most extensive antibiotic consumer [32], exhibiting a high AMR rate for penicillin (87.41%), and ampicillin (64.52%) (Figure S3 in the **Online Supplementary Document**). First-line antibiotics, including ciprofloxacin (16.33%) and ceftriaxone (12.41%), were still comparatively effective.

We also found the dynamics of AMR during 1991–2021, where FA and food data were referred to collectively as non-human origin due to limited samples (**Figure 5**, Panel B). The trend of the two origins corresponded for most antimicrobial compounds, strongly supporting the significance of food chain upstream control from livestock. The overall AMR rate of non-human origin increased sharply during 1991–2015, due to wide prophylactic antibiotics usage in livestock, including quinolone, aminoglycoside, sulfonamide, β-lactam, cephem, phenicol, and tetracycline (**Figure 5**, Panels B–M). The effectiveness of the ban on colistin and other antibiotics as feed additives in 2017 and 2020 was confirmed by the AMR rate downward trend in the antibiotics mentioned above as well as in penicillin, carbapenem, polymyxin, and macrolide during 2016–20. The explanation of AMR strains losing their niche can be looked for in the trade-off between AMR and fitness cost, including thermal, alkali, acid tolerance, and biofilm, as in previous studies [33,34]. Our results confirmed the prosperity of antimicrobial withdrawal in reversing the severity of clinical AMR issues, despite excessive use in livestock production during the past 50 years [35]. In addition, inadequate sampling may be the underlying reason for the abnormally high resistance rate against carbapenem in humans before 2011.

## DISCUSSION

Food safety was highlighted as a ‘grave concern and national priority’ by the Chinese government. *Salmonella* is the most problematic in this context according to the statistics from PulseNet China [36], as it is known for its widespread presence and association with gastroenteritis, as well as invasive complications. Non-typhoidal salmonellosis is considered a priority for global surveillance, usually occurring after food consumption derived from FA carriers, and the risk level is highly region-dependent due to economic development and hygiene conditions, which may be the underlying reason for north-south prevalence disparity in China [22]. To our knowledge, this is the first meta-analysis investigating geographical and temporal NTS under the One Health context in China.

Based on 562 studies, we observed a higher threat of NTS in northern China than in the south, suggesting that closer attention should be paid to food processing and sanitation procedures. We expected our conclusions from this review to contradict those of the National Foodborne Disease Outbreak Surveillance [4], considering that more breakdowns occurred in the Chinese farm-to-fork continuum than in industrialised countries, especially in low-income regions of northern China, indicating that the deployment of more sophisticated surveillance system is necessary [36,37]. Additionally, the decrease in the overall prevalence before 2011 can be attributed to the administration of animal epidemic prevention and food control, while afterwards, the burden of human NTS infection increased significantly, which can be attributed to the emergence of virulent and multidrug-resistant clones [38,39]. The situation calls for advanced surveillance and control strategies in China.

Though thousands of serovars lead to identical clinical conditions of NTS infections, some subtypes are associated with invasive diseases, such as *S. *Typhimurium ST313 [7], which adopted a stepwise pseudogenisation evolutionary trajectory that resembled other host-restricted and invasive serovars [40]. Hence, investigating predominant serovars is necessary for monitoring purposes and further insights into prevalent mechanisms. We reported that, after 2006, *S. *Typhimurium and *S. *Enteritidis emerged as the predominant clinical threats, replacing *S. *Derby and *S. *Agona. This shift may be due to significant subtype substitution, which enhanced transmission, colonisation, and AMR abilities, such as the replacement of biphasic ST29 with monophasic ST34 in *S. *Typhimurium [41–43]. Additionally, *S*. Stanley has increased the disease burden recently, along with *S. *Typhimurium, possibly due to the cold-blooded animal handling and prorogation of Typhimurium ST34 [44]. Despite subtype shift and serovar competition, most of the prevalent serovars belong to serogroup O: 9, O: 4, and O:8 in China, the EU, and the US [45,46]. Fast and precise antigen detection methods should be generalised in producing factories and farms to enable quick recognition and response.

Host adaptation of serovars and regional diet preference can significantly impact the regional prevalence patterns. Notably, *S*. Newport and *S*. Kentucky, the most dominant serovars in the EU and the US, ranked 12 (1.13%) and 15 (0.61%) in the top fifteen serovars in our study. This difference may be due to the poultry preference of the two serovars and the diet preference for poultry instead of swine [16]. Poultry, especially turkey, are major reservoirs of *S*. Javiana (O: 9), *S. *Saintpaul (O: 4) and *S. *Hadar (O: 8), respectively ranking clinically fourth in the US, Oceania, and EU [45,47]. However, these serovars were only sporadic in China, likely due to low turkey consumption. Considering specific serovars can be host-adapted, previous studies have evaluated the risk of various food commodities in disseminating foodborne pathogens and the geographical difference [11,16]. However, no such extensive research on NTS is available in China, which is crucial for understanding host preference and the contribution of each host to the clinical burden. Our PCA analysis showed that poultry was an essential vehicle of *S. *Enteritidis and *S. *Indiana transmission, while swine and ruminants were the primary carriers of *S. *Typhimurium and *S. *Derby. Poultry farms are considered the leading reservoirs for disseminating NTS [46,48]. Consistently, poultry-derived food products constitute a significant source of NTS [13]. These results agreed with a previous study in the US that showed egg-based dishes were the most common food vehicle associated with outbreaks of *S. *Enteritidis [49]. Moreover, *S. *Indiana was reported among the top three dominant serovars in chickens, which can be transmitted to humans, leading to severe cases of NTS [50]. Conversely, pigs were reported to be a significant reservoir for *S. *Typhimurium and *S. *Derby in China and other regions [16,51]. Importantly, we confirmed the dominance of *S. *Typhimurium and *S. *Enteritidis in humans, suggesting the possible transfer of these serovars from poultry and pigs’ production chains to humans. Therefore, when evaluating the risk of hosts carrying dominant serovars, poultry is defined as a key vehicle for transmitting *S. *Enteritidis and *S*. Indiana, while swine and ruminants are associated with *S. *Typhimurium and *S. *Derby. The condition in northern swine, however, is more complex. Again, we identified the farm-to-fork impact of NTS, not only for regional prevalence, but also for dominant serovars. The findings regarding host preference highlight the importance of tailed mitigation programs, rather than general ones, for different hosts in addressing specific dominant serovars. Although the reasons for the variation in serovar distribution among hosts remain unclear, researchers have agreed that host adaptation is primarily influenced by the ability of the serovars to adhere to and invade host cells, as well as the level of inflammation they induce [52,53].

The failure of therapies for NTS infections due to AMR continues to be a public health threat, driven by excessive antibiotic use in food-animal production. China has been the biggest antibiotic consumer since 2017, accounting for 45% of global use, with this proportion projected to be 43% in 2030 [20]. We reported an overall AMR rate of 73.63%, with higher resistance observed in *Salmonella* from non-human sources compared to those from human sources, especially in swine, a significant antibiotic consumer [18]. This is attributed to the overuse and misuse of antimicrobial agents in animal farming, which promotes the development and selection of multidrug-resistant strains. Hence, the indiscriminative and extensive use of antimicrobials in livestock, particularly as feed additives in the swine industry in China. This poses a significant challenge for the treatment of foodborne non-typhoidal salmonellosis. Importantly, the ban on feed-based antibiotics has been succesful, leading to a reduction in AMR in both non-human and human origins, and ensuring that first-line and last-resort drugs remain relatively effective. However, antimicrobial susceptibility tests and cautious choices of antibiotics are recommended to avoid accumulating AMR selection pressure [44].

Our systematic review and meta-analysis determined the temporal and spatial prevalence of NTS across a wide range of hosts, emphasising the importance of specific and targeted interventions in food chain components. Although a reduction in AMR was observed after 2015, the systematic monitoring system still requires ongoing implementation. Nevertheless, this studyprovided a comprehensive database and baseline knowledge on NTS from 1953 to 2021, offering valuable insights to guide targeted and rational policymaking using the One Health approach.

## CONCLUSIONS

NTS is a commonly reported foodborne zoonotic agent with pressing public health concerns. The One Health approach has shown that spatiotemporal distribution and AMR patterns of NTS are closely linked with animal-derived food products and subsequent human infections. We identified a temporal shift in dominant serovars in different hosts, emphasising the importance of a more comprehensive surveillance system, a tailored mitigation program for a diverse range of *Salmonella* serovars, and the effectiveness of the ban on prophylactic antibiotics. This study provides a key reference for assessing the disease burden and conducting risk evaluations associated with NTS infections.

## Additional material


Online Supplementary Document
