# Supplementary material for: One Health approach probes zoonotic non-typhoidal Salmonella infections in China: A systematic review and meta-analysis
Source: J Glob Health. 2024 Dec 2;14:04256. doi: 10.7189/jogh.14.04256 (PMC11610537; doi:10.7189/jogh.14.04256)
Supplement: Online Supplementary Document [file jogh-14-04256-s001.pdf]

ONE Health approach probes zoonotic non-typhoidal *Salmonella* infections in China

Jiaqi Chen [1,2], Linlin Huang [2], Hongli An [2,3], Zining Wang [1,2], Xiamei Kang [2], Rui Yin [2], Chenghao Jia [2], Xiuyan Jin [2], Min Yue [1, 2, 3, 4*]

[1] *Key Laboratory of Systems Health Science of Zhejiang Province, School of Life Science, Hangzhou Institute for Advanced Study, Hangzhou 310024; University of Chinese Academy of Sciences, China*

[2] *Department of Veterinary Medicine, Zhejiang University College of Animal Sciences, Hangzhou 310058, China; 22117046@zju.edu.cn (J.C.); 22217045@zju.edu.cn (L.H.); anhongli@zju.edu.cn (H.A.); znwang@zju.edu.cn (Z.W); abdelaziz_iaa@yahoo.fr (A.E-D); 12017032@zju.edu.cn (X.K.); 22117103@zju.edu.cn (R.Y.); 22017118@zju.edu.cn (C.J.); 22017052@zju.edu.cn (X.J.)*

[3] *Hainan Institute of Zhejiang University, Sanya 572025, China*

[4] *State Key Laboratory for Diagnosis and Treatment of Infectious Diseases, National Clinical Research Center for Infectious Diseases, National Medical Center for Infectious Diseases, The First Affiliated Hospital, Zhejiang University School of Medicine, Hangzhou 310003, China.*

* Correspondence: Min Yue (myue@zju.edu.cn or myue@ucas.ac.cn); Tel./Fax: +86-571-88982832

Contents

Figure S1 3
Figure S2, panel a 4
Figure S2, panel b 4
Figure S2, panel c 5
Figure S3 6
Table S1-6 7

Figure S1. Temporal changes of sample size and prevalence during 1953-2021 in (a) food animals (FA), (b) food, (c) humans, (d) poultry, (e) swine, and (f) ruminants during 1953-2021 in China. The black bars indicate the sample size on the left axis, and the red lines indicate prevalence on the right axis.

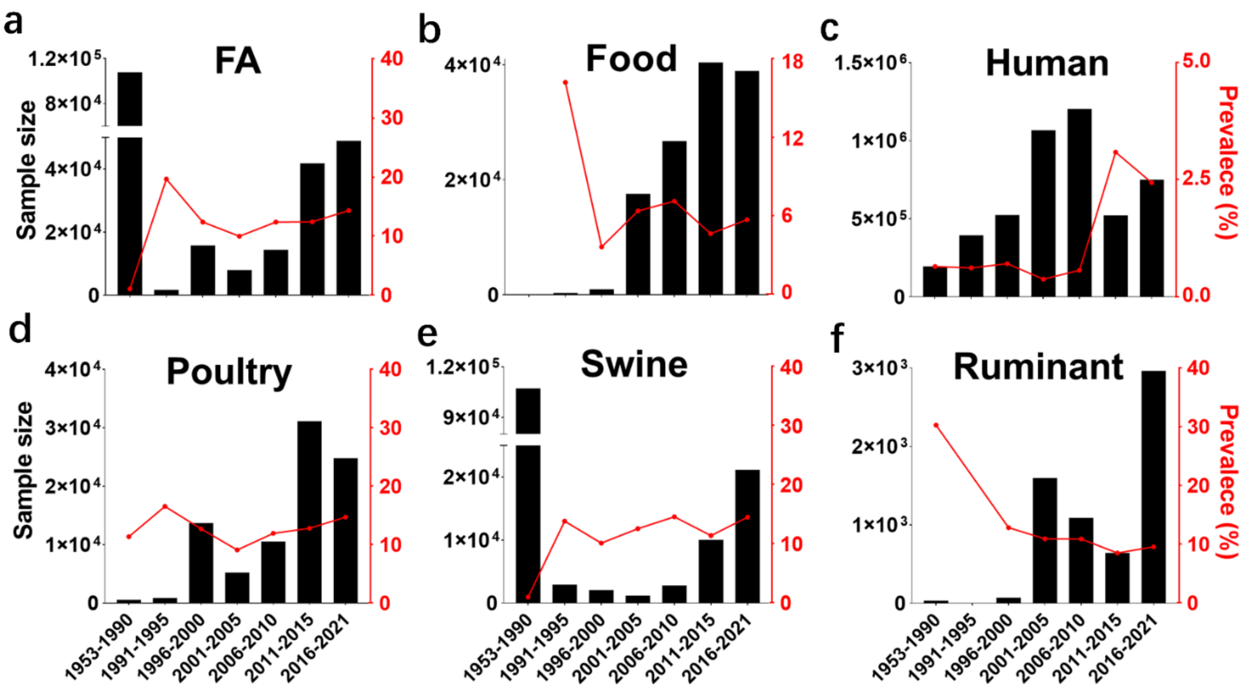

Figure S2. Top fifteen serovars and proportion in isolates serotyped from (a) food animals (FA), (b) food, (c) humans during 1953-2021. Total stands for the number of isolates serotyped from each origin.

a

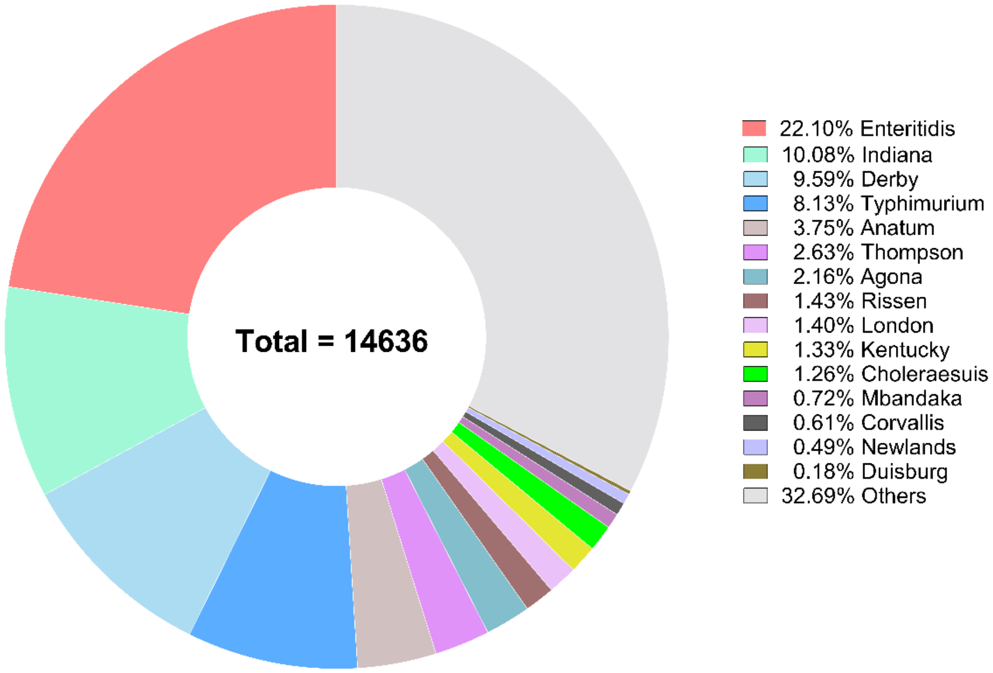

b

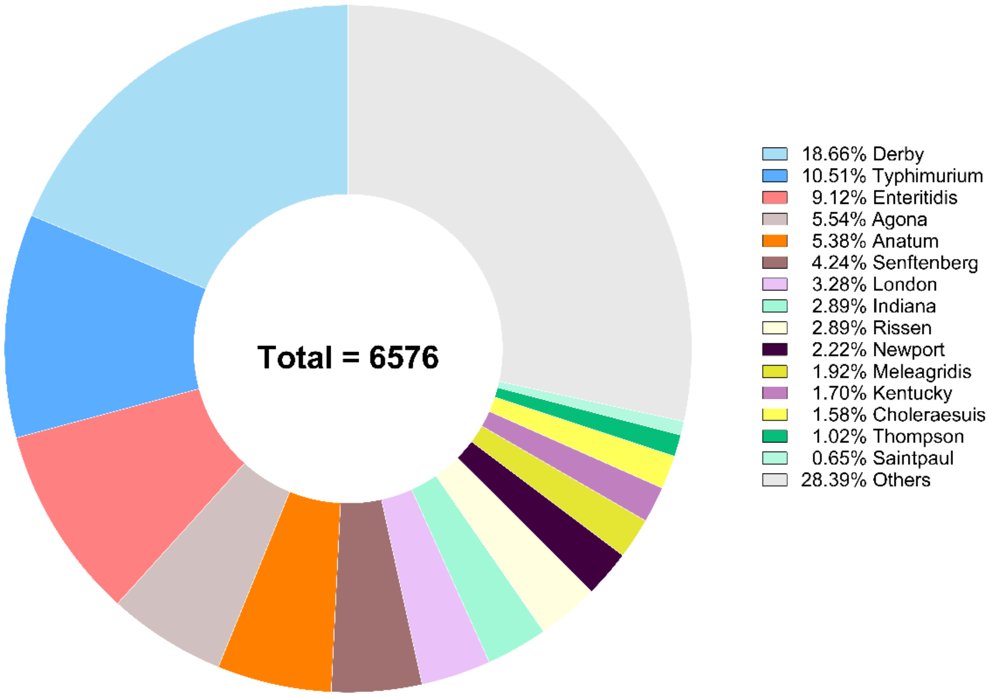

c

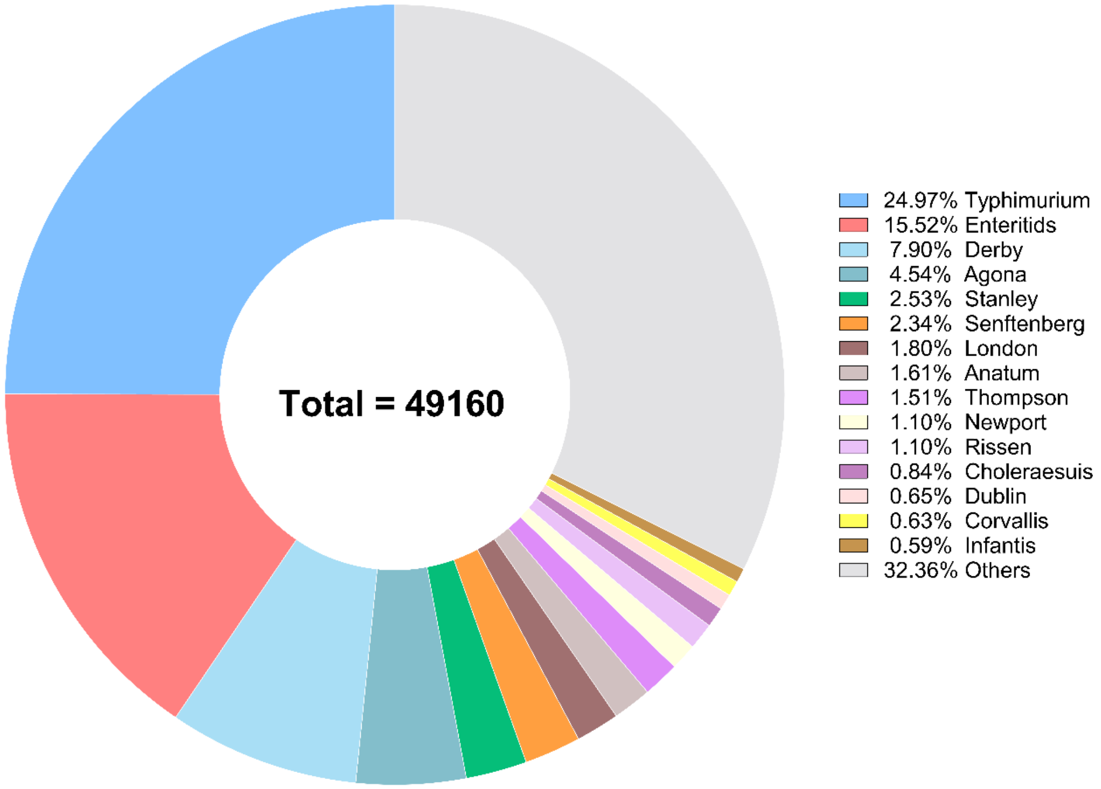
Total = 49160
24.97% Typhimurium
15.52% Enteritids
7.90% Derby
4.54% Agona
2.53% Stanley
2.34% Senftenberg
1.80% London
1.61% Anatum
1.51% Thompson
1.10% Newport
1.10% Rissen
0.84% Choleraesuis
0.65% Dublin
0.63% Corvallis
0.59% Infantis
32.36% Others

Figure S3. Antimicrobial resistance rate of isolates from poultry (P), swine (S), ruminants (R), collectively referred as food animals (FA), food (F), human (H), and all sources (ALL) against antimicrobial class Penicillin, including penicillin (PEN), ampicillin (AMP) and streptomycin (STR); class Quinolone, including nalidixic acid (NAL), ciprofloxacin (CIP); class Aminoglycoside, gentamicin (GEN) and kanamycin (KAN); and Cephem, cefotaxime (CTX), ceftazidime (CAZ), cefazolin (CZ), cephalothin (CEP), ceftriaxone (CRO), cefoxitin (FOX) and cefepime (FEP). The dark grey with a cross indicates that data is not available.

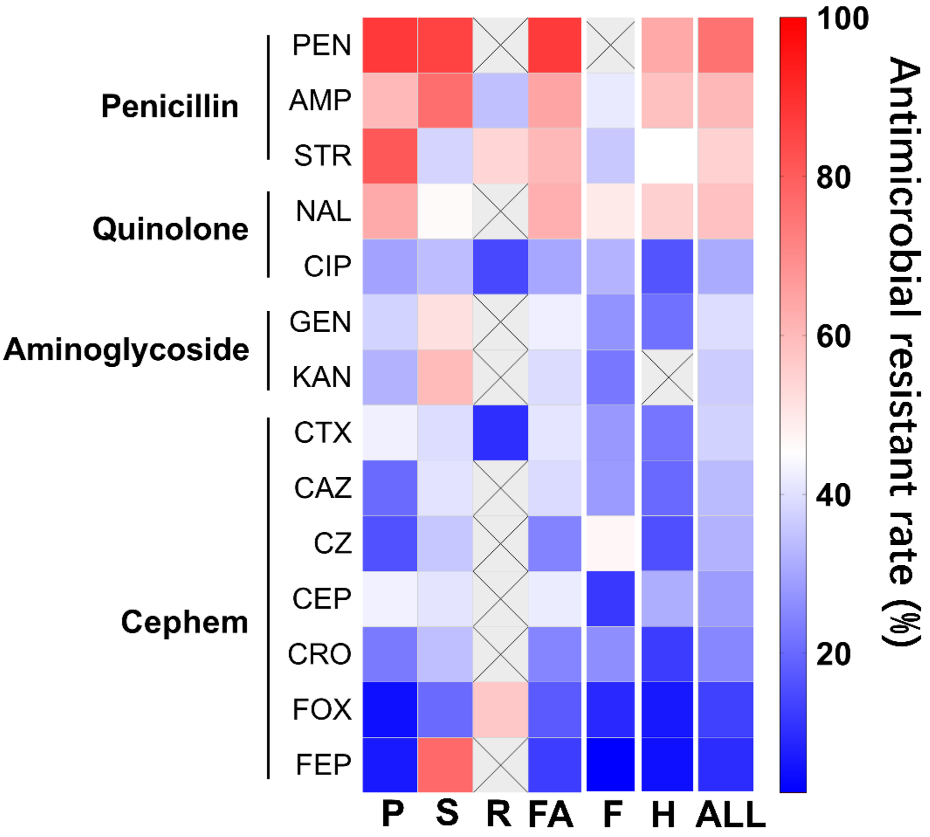

Table S1

No.	Literature reference	Title	Publication year
18	DOI:10.19568/j.cnki.23-1318.2021.01.0026	综合医院食源性疾病病原学监测结果分析	2021
40	DOI:10.19568/j.cnki.23-1318.2021.01.0026	乌鲁木齐地区不同健康状态和饲养环境中宠物源沙门菌耐药特征分析	2021
45	DOI:10.3969/j.issn.1002-1957.2021.03.042	湖南湘潭县屠宰生猪沙门氏菌和链球菌感染情况调查及耐药性分析	2021
51	ISSN:1007-2705	北京市海淀区2015-2019年食源性疾病病原检测结果分析	2021
54	DOI:10.19485/j.cnki.issn2096-5087.2021.03.024	温州市食源性疾病流行特征分析	2021
65	DOI:10.19428/j.cnki.sjpm.2021.20536	上海市嘉定区腹泻患者沙门菌流行病学特征及耐药性分析	2021
69	DOI:10.13820/j.cnki.gdyx.20200787	2016—2018年广州地区儿童非伤寒沙门菌感染的临床流行病学特征	2021
79	DOI:10.3969/j.issn.1672-3619.2021.06.029	2017-2019年玉林市饮服从业人员沙门菌检测及分型研究	2021
98	ISSN:0529-6005	辽宁辽南地区庄河大骨鸡沙门菌病原分离、及药敏试验	2021
110	DOI:10.19812/j.cnki.jfsq11-5956/ts.2021.08.010	生猪屠宰环节沙门氏菌携带状况、耐药性及其耐药基因分析	2021
111	DOI:10.7619/jcmp.20211991	山西省儿童医院2014-2019年小儿感染性腹泻细菌谱及耐药性变化分析	2021
113	DOI:10.16506/j.1009-6639.2021.02.003	2017-2019年北京市感染性腹泻细菌病原谱与流行特征分析	2021
114	ISSN:2096-5249	珠海某医院腹泻患者粪便沙门菌血清分型及药敏情况分析	2021
126	DOI:10.3969/j.issn.1671-7414.2021.04.011	2018～2020年西安地区儿童食源性腹泻病病原菌分布特征和耐药性及鼠伤寒沙门菌聚类分析	2021
129	ISSN:1007-2705	深圳市龙岗区2020年从业人员沙门菌携带情况调查	2021
135	DOI:10.13493/j.issn.1672-7878.2021.03-026	628例食源性腹泻婴幼儿粪便与肛拭子标本中病原菌的及主要致病菌谱的分析及其防治对策	2021
142	DOI:10.12325/j.issn.1672-5336.2021.12.047	细菌性食物中毒的病原学情况与微生物检验结果研究	2021
145	DOI:10.3784/jbjc.202103110118	黑龙江省人源沙门菌流行特征及指纹图谱多态性分析	2021
150	DOI:10.3784/jbjc.202105150270	2018-2020年浙江省湖州市食源性疾病监测结果分析	2021
165	DOI:10.13429/j.cnki.cjcr.2021.08.016	南京市肠道感染性腹泻样本中食源性致病菌分离及耐药监测分析	2021
168	DOI:10.3969/j.issn.1006-1959.2021.16.045	2017年～2019年北京市顺义区成年人食源性疾病主动监测结果分析	2021
169	DOI:10.16372/j.issn.1004-6364.2021.06.011	肉鸡屠宰环节沙门菌和弯曲杆菌的流行性调查及耐药性分析	2021
179	DOI:10.13590/j.cjfh.2021.03.010	北京市顺义区腹泻病例分离沙门氏菌耐药特征分析	2021
192	ISSN:1004-1257	2015-2019年北京市海淀区感染性腹泻病原菌监测结果	2021
200	ISSN:1003-9961	北京市密云区腹泻相关病原流行特征及腹泻病原菌耐药谱研究	2021
202	DOI:10.13344/j.microbiol.china.210357	重庆市北碚区宠物源沙门氏菌耐药性监测及ESBL和PMQR基因检测	2021
218	DOI:10.19485/j.cnki.issn2096-5087.2021.06.023	三门县食源性疾病监测结果分析	2021
233	DOI:10.13329/j.cnki.zyyjk.2021.0457	2016-2019年北京市通州区食源性疾病主动监测病原体流行特征分析	2021
239	DOI:10.11847/zgggws1128260	南京市2018年食源性疾病主动监测流行病学特征	2021
246	DOI:10.13590/j.cjfh.2021.05.005	2018-2020年天津市津南区食源性致病菌分布及药敏分析	2021
250	ISSN:1672-2116	2015-2019年北京市海淀区食源性腹泻病原监测结果流行特征分析	2021
259	DOI:10.12259/j.issn.2095-610X.S20210713	2017年至2020年儿童感染性腹泻沙门菌和志贺菌分布及药敏结果	2021
261	DOI:10.3969/j.issn.1006-3110.2021.08.029	2018-2019年金山区食源性致病菌污染情况	2021
264	DOI:10.13329/j.cnki.zyyjk.2021.0266	2019年扬州市食源性疾病主动监测病原学结果分析	2021
271	DOI:10.3969/j.issn.1008-0589.202101031	2019年～2020年河北地区鸡源沙门菌流行病学调查及耐药性分析	2021
292	ISSN:1003-8507	2016-2018年江西省食源性腹泻患者沙门氏菌感染状况和耐药性分析	2020
294	DOI:10.19812/j.cnki.jfsq11-5956/ts.2020.24.053	2011～2018年吉林省食品中沙门氏菌的污染监测及血清型别分布	2020
303	DOI:10.3969/j.issn.1001-361X.2020.12.024	食源性沙门氏菌的分离与生物学特性分析	2020
304	ISSN:1004-8685	腹泻病原在儿童中的感染流行特征及耐药性分析	2020
306	DOI:10.13344/j.microbiol.china.200631	苏北地区白羽肉鸡父母代种鸡及其商品鸡中沙门氏菌的分离、与生物特性	2020
309	DOI:10.19568/j.cnki.23-1318.2020.06.032	余姚市2007-2018年感染性腹泻病原谱监测分析	2020
315	DOI:10.19812/j.cnki.jfsq11-5956/ts.2020.23.074	上海地区市售生鲜肉中单核细胞增生李斯特菌和沙门氏菌的污染监测分析	2020
317	DOI:10.11816/cn.ni.2020-200819	遵义地区儿童感染性腹泻病原学及其sIL-2R诊断价值	2020
323	ISSN:1672-1721	小儿感染性腹泻452例临床和病原学分析探索	2020
329	DOI:10.13590/j.cjfh.2020.06.016	2015—2018年上海市浦东新区食源性疾病主动监测病原学及流行病学特征分析	2020
348	DOI:10.3969/j.issn.1007-5038.2020.11.011	吉林省规模化种鸡场沙门菌血清型与毒力基因及耐药性检测	2020
355	DOI:10.13329/j.cnki.zyyjk.2020.0803	2014-2019年上海市浦东新区食源性致病菌监测分析	2020
356	DOI:10.3969/j.issn.1006-5180.2020.20.032	余姚市2019年食源性沙门菌血清分布及药敏结果分析	2020
373	ISSN:1004-8685	2015年-2018年哨点医院食源性疾病主动监测结果	2020
381	DOI:10.19812/j.cnki.jfsq11-5956/ts.2020.19.072	辽宁省不同来源沙门氏菌耐药性分析	2020
397	DOI:10.3969/j.issn.1672-3619.2020.09.028	沙门菌感染急性腹泻患儿预后及危险因素分析	2020
400	ISSN:1006-4028	成都市沙门菌感染患儿160例临床特征及病原学分析	2020

411	DOI:10.13329/j.cnki.zyyjk.2020.0658	2017-2018年天津市津南区食源性疾病病原体监测结果	2020
417	ISSN:0529-5130	徐州及周边地区鸭源沙门菌的分离及其耐药性、毒力基因分析	2020
423	DOI:10.3969/j.issn.1672-3619.2020.08.029	2016-2018年上海市奉贤区腹泻病流行特征	2020
426	DOI:10.16303/j.cnki.1005-4545.2020.08.13	西藏部分地区藏猪源沙门菌分离及耐药表型	2020
427	DOI:10.3969/j.issn.1002-2694.2020.00.094	2010-2018年安徽省马鞍山市腹泻患者中非伤寒沙门菌的分子分型与耐药性研究	2020
434	DOI:10.3969/j.issn.1004-437X.2020.24.070	细菌感染性腹泻患儿粪便及抗菌药物敏感性分析	2020
442	DOI:10.19812/j.cnki.jfsq11-5956/ts.2020.15.039	2019年江苏省部分地区儿童腹泻沙门氏菌的感染率及耐药状况研究	2020
446	ISSN:1004-8685	余姚市食源性疾病特定病原体主动监测结果分析	2020
447	ISSN:1008-3847	广东省某肉鸡养殖场沙门菌的分离与耐药性检测	2020
451	DOI:10.3760/cma.j.issn.1008-6706.2020.14.025	2014－2017年北京市昌平区成年人急性感染性腹泻1 520例病原体检测结果分析	2020
453	DOI:10.13590/j.cjfh.2020.04.014	2018年上海市非伤寒沙门菌感染流行病学特征分析	2020
457	DOI:10.14053/j.cnki.pPCR.202007014	2017-2019年新疆乌鲁木齐地区急性感染性腹泻常见病原菌分布及其耐药性分析	2020
462	DOI:10.19812/j.cnki.jfsq11-5956/ts.2020.14.037	2018年6～10月河北省市售猪肉馅及冷鲜鸡中沙门氏菌污染情况调查与分析	2020
467	DOI:10.19812/j.cnki.jfsq11-5956/ts.2020.13.021	2018～2019年云南省沙门氏菌感染引起食源性腹泻状况分析	2020
470	DOI:10.7683/xxyxyxb.2020.07.006	2019年郑州市食源性疾病流行特征及优势菌群分析	2020
474	DOI:10.13304/j.nykjdb.2019.0204	新疆焉耆县不同动物源耐药沙门氏菌的MLST分析	2020
477	DOI:10.3969/j.issn.1009-881X.2020.02.024	2012年—2019年苏州地区儿童肠道沙门菌感染状况及耐药性分析	2020
478	DOI:10.16303/j.cnki.1005-4545.2020.06.12	2018年湖北省猪源沙门菌的分离及耐药性分析	2020
503	DOI:10.3969/j.issn.1006-3765.2020.05.093	2017～2019年我院沙门菌感染情况和耐药性分析	2020
509	ISSN:1003-8507	2014-2018年铜陵市食品中食源性致病菌监测结果分析	2020
515	DOI:10.16281/j.cnki.jocml.2020.40.013	急性腹泻患者非伤寒沙门菌的耐药性检测和基因分型研究	2020
561	ISSN:1003-8507	2018年陕西省感染性腹泻患者中分离沙门菌的病原学特征分析	2020
563	ISSN:1004-8685	余姚市食源性疾病病原菌监测结果分析	2020
571	DOI:10.3760/cma.j.issn.0254-6450.2020.03.026	上海市浦东新区2013-2017年感染性腹泻病原流行特征分析	2020
576	DOI:10.3784/j.issn.1003-9961.2020.03.008	基于方法学评估与检测基线预测广西玉林市从业人员沙门菌带菌水平	2020
577	DOI:10.3969/j.issn.1672-3619.2020.03.032	2015-2017年广州某医院儿童腹泻患者沙门菌感染的血清型和耐药性	2020
579	ISSN:1004-8685	2015年-2018年舟山市食源性疾病住院病例流行特征及病原分布分析	2020
586	DOI:10.13329/j.cnki.zyyjk.2020.0199	2014-2018年北京市房山区腹泻监测病例沙门菌监测结果	2020
600	DOI:10.6048/j.issn.1001-4330.2020.02.014	牛羊源沙门氏菌在某定点屠宰场和农贸市场的污染分布与耐药性调查	2020
654	DOI:10.13329/j.cnki.zyyjk.2020.0012	2015-2018年上海市宝山区食源性疾病病原监测结果分析	2020
657	DOI:10.13329/j.cnki.zyyjk.2020.0127	玉林市婴幼儿腹泻感染非伤寒沙门菌分子分型和耐药情况分析	2020
666	DOI:10.3969/j.issn.1672-3619.2019.12.024	深圳市龙华区其他感染性腹泻人群病原构成及耐药情况分析	2019
684	DOI:10.3784/j.issn.1003-9961.2019.08.011	2014-2018年浙江省余姚市非伤寒沙门菌监测及其耐药谱分析	2019
704	DOI:10.3969/j.issn.1003-6350.2019.13.017	2016-2018年阳江市人民医院沙门菌监测及其病原学特征	2019
705	DOI:10.3969/j.issn.1671-5217.2019.04.087	门诊感染性腹泻沙门菌监测结果分析	2019
719	DOI:10.3969/j.issn.1006-3110.2019.10.034	北京市昌平区感染性腹泻标本常见病原菌谱分析	2019
726	ISSN:1001-7062	2018年昌平区感染性腹泻监测结果分析	2019
738	DOI:10.3784/j.issn.1003-9961.2019.04.008	2013-2015年安徽省合肥市食源性疾病患者分离沙门菌的病原学特征分析	2019
750	DOI:10.3969/j.issn.1006-2483.2019.05.025	2015—2018年珠海市10岁及以下儿童沙门菌感染情况	2019
764	DOI:10.19812/j.cnki.jfsq11-5956/ts.2019.22.022	安宁市食品从业人员携带沙门氏菌血清型分布特征及耐药性分析	2019
766	DOI:10.16372/j.issn.1004-6364.2019.24.018	浙江省部分地区鸭源沙门菌流行情况及耐药性调查	2019
772	DOI:10.16498/j.cnki.hnnykx.2019.007.002	湖南地区猪源和鸡源沙门氏菌的分离与耐药性分析	2019
778	DOI:10.16462/j.cnki.zhjbkz.2019.07.026	2015-2017年泰州市沙门菌污染及其感染检出状况分析	2019
784	DOI:10.3969/j.issn.1673-5501.2019.03.003	1991至2017年某儿童专科医院儿童细菌性腹泻病原菌分布变迁	2019
794	DOI:DOI:10.19485/j.cnki.issn1007-0931.2016.05.006	婴幼儿感染性腹泻病原微生物及耐药性探究	2019
798	DOI:10.3969/j.issn.1673-4130.2019.11.011	成都市某医院腹泻儿童非伤寒沙门菌感染状况及耐药分析	2019
800	DOI:10.13217/j.scjpm.2019.0045	2016-2017年河源市腹泻病例沙门菌感染情况分析	2019
816	DOI:10.16659/j.cnki.1672-5654.2019.11.155	河南省食品加工从业人员食源性致病菌带菌状况 监测分析	2019
833	DOI:10.3969/j.issn.1672-3619.2019.07.025	2012-2017年福田区食品中沙门菌污染状况监测及分子特征研究	2019
834	DOI:10.16372/j.issn.1004-6364.2019.09.006	2015~2018年华东地区野禽沙门菌的分离及耐药性分析	2019
854	DOI:10.13881/j.cnki.hljxmsy.2018.07.0325	重庆地区鸡源大肠杆菌、沙门氏杆菌耐药性的测定与分析	2019
863	DOI:10.3969/j.issn.1005-944X.2019.04.018	河南省猪源沙门氏菌分离、血清分型及药物敏感性分析	2019
874	DOI:10.3784/j.issn.1003-9961.2019.09.007	2016-2018年北京市通州区感染性腹泻细菌病原谱监测分析	2019
875	DOI:10.3969/j.issn.1674-9316.2019.06.003	饮食和服务行业健康人群携带沙门菌调查	2019

882	DOI:10.3969/j.issn.2095-1191.2019.10.28	2015—2017年广西鸡源沙门氏菌耐药性与致病性的相关性分析	2019
884	DOI:10.16303/j.cnki.1005-4545.2019.08.19	辽宁省规模化猪场生产链中沙门菌优势血清型及其主要毒力因子分布	2019
885	DOI:10.13515/j.cnki.hnjpm.1006-8414.2019.010.023	2015-2018年洛阳市食品中沙门氏菌污染状况及血清型分布情况	2019
887	DOI:10.13590/j.cjfh.2019.05.012	温州市食品中沙门菌污染状况及特征分析	2019
896	DOI:10.3784/j.issn.1003-9961.2019.06.010	2013-2017年北京市门头沟区感染性腹泻病原菌监测结果分析	2019
908	DOI:10.7629/yxdwfz201902008	2015-2017年河北省廊坊市食品中沙门氏菌污染状况及血清型监测分析	2019
909	DOI:10.11748/bjmy.issn.1006-1703.2019.05.014	北京大型社区成人感染性腹泻肠道致病菌监测与耐药性分析	2019
923	ISSN:1004-8685	北京市丰台区2016年-2017年食源性疾病监测分析	2019
924	DOI:10.16408/j.1004-9770.2019.05.012	梧州市急性感染性腹泻的病原菌及耐药性分析	2019
931	DOI:10.16718/j.1009-7708.2019.04.015	2017年深圳市儿童医院临床分离细菌分布及耐药性监测	2019
946	DOI:10.3969/j.issn.1672-9455.2019.17.031	某市腹泻患者中沙门氏菌感染状况及耐药状况的分析	2019
961	ISSN:1004-8685	2017年丰台区肠道致病菌检出及耐药情况分析	2019
963	DOI:10.3969/j.issn.1674-9316.2019.07.035	上海市嘉定区儿童感染性腹泻病原菌检测结果分析	2019
964	ISSN:1006-4028	2014-2017年深圳市龙华区食源性所致感染性腹泻病原学分析	2019
984	DOI:10.16050/j.cnki.issn1674-6309.2019.09.014	2010—2016年沙坡头区感染性腹泻细菌病原谱监测结果分析	2019
991	DOI:10.14170/j.cnki.cn31-1278/s.2019.01.019	上海部分鸡场禽沙门菌血清检测分析	2019
997	ISSN:1004-8685	2015年-2017年山西省阳泉市食源性多病原监测与溯源分析	2019
1001	DOI:10.13329/j.cnki.zyyjk.2019.0699	2018年汉中市食源性沙门菌血清分型及PFGE的分子分型研究	2019
1002	ISSN:1003-8507	2017年郑州市食源性疾病病原学监测结果分析	2019
1009	DOI:10.16506/j.1009-6639.2019.01.004	2016年浙江省食源性疾病监测结果分析	2019
1013	ISSN:1004-8685	浙江常山食源性疾病病原菌分布与耐药性研究	2019
1014	DOI:10.3969/j.issn.1001-8689.2019.02.019	2015-2017年中山市某医院沙门菌的药敏分析及肠毒素基因检测	2019
1024	DOI:10.3969/j.issn.1002-2694.2019.00.094	山西省2015-2016年食源性疾病主动监测的病原学特征分析	2019
1030	DOI:10.3969/j.issn.1005-0019.2018.20.402	上海市黄浦区感染性食源性疾病流行特征及病原学分析	2018
1032	DOI:10.3760/cma.j.issn.1673-4912.2018.02.011	2013至2015年中山市儿童肠道感染非伤寒沙门菌流行特征及耐药性分析	2018
1040	DOI:10.13329/j.cnki.zyyjk.2018.0899	2017年北京市门头沟区食源性疾病主动监测结果	2018
1043	ISSN:1672-9692	辽宁省鸡源沙门氏菌血清型与耐药性分析	2018
1046	DOI:DOI:10.3760/cma.j.cn101070-20200317-00432.	广州地区腹泻儿童沙门菌感染状况调查及耐药性分析	2018
1056	DOI:10.11852/zgetbjzz2018-26-05-28	儿童细菌感染性腹泻分离菌株的分布和耐药性分析	2018
1062	ISSN:1672-9692	鸡肠炎沙门氏菌的分离与药敏试验	2018
1074	ISSN:1003-8507	成都市从业人员43株肠道沙门菌血清分型与耐药状况	2018
1079	DOI:10.3969/j.issn.1002-2694.2018.00.119	2011-2013年河南省沙门菌污染分布状况及其耐药研究	2018
1081	DOI:10.16303/j.cnki.1005-4545.2018.07.11	种鸡场孵化死胚中沙门菌的分离与耐药性分析	2018
1094	DOI:10.16833/j.cnki.jbmc.2018.02.037	不同饲养方式下牛源沙门氏菌的流行特征	2018
1095	DOI:10.3969/j.issn.1002-2694.2018.00.132	山东地区屠宰场猪肉污染沙门氏菌菌株毒力基因筛查与ERIC-PCR分型	2018
1098	DOI:10.16833/j.cnki.jbmc.2018.01.043	包头市不同饲养方式下牛源沙门氏菌的耐药性	2018
1100	DOI:10.16408/j.1004-9770.2018.02.003	威海地区腹泻致病菌及药敏分析	2018
1103	ISSN:1006-4028	2015-2017年南充市熟肉制品加工过程微生物污染情况监测	2018
1106	DOI:10.3969/j.issn.1673-4130.2018.05.035	2013-2016年广州地区沙门菌感染流行病学特征和耐药性分析	2018
1127	DOI:10.13329/j.cnki.zyyjk.2018.0326	2015-2016年北京市房山区腹泻患者沙门菌耐药性及飞行mass spectrometry的聚类分析	2018
1140	ISSN:1004-8685	2015年-2017年桐庐县食源性特定病原菌监测结果分析	2018
1148	DOI:10.13329/j.cnki.zyyjk.2018.0532	2017年沧州市食品微生物及其致病因子风险监测结果	2018
1150	DOI:10.16372/j.issn.1004-6364.2018.04.018	肉鸡养殖加工中沙门菌和弯曲杆菌污染研究	2018
1159	ISSN:1004-8685	丽水市食源性腹泻病原体检测结果分析	2018
1175	DOI:10.19813/j.cnki.weishengyanjiu.2018.02.017	2015-2016年淮安市售禽畜肉中沙门菌污染及其病原学特征	2018
1180	DOI:10.3969/j.issn.1002-2694.2018.00.037	成都地区鸭源沙门氏菌的分离及其耐药特征、毒力基因分析	2018
1186	DOI:10.7629/yxdwfz201801032	2016年河北省三河市餐服人员沙门氏菌检出情况分析	2018
1189	ISSN:1003-8507	淄博市342份市售生肉中食源性沙门菌的型别特点及耐药性	2018
1190	DOI:10.3969/j.issn.1672-3619.2018.10.029	2014-2016年中山市食品从业人员肠道致病菌带菌状况分析	2018
1194	DOI:10.3969/j.issn.1002-2694.2018.00.124	2013-2014年贵阳市感染性腹泻监测病例中沙门菌的病原学监测分析	2018
1213	DOI:10.3784/j.issn.1003-9961.2018.10.004	2014-2017年北京市顺义区腹泻病例中沙门菌流行特征与分子分型特征分析	2018
1225	DOI:10.3969/j.issn.1673-8640.2018.12.009	急性感染性腹泻沙门菌分离株的耐药谱及其相关基因分析	2018
1235	DOI:10.16372/j.issn.1004-6364.2018.05.018	河南省肉鸡沙门菌血清型分布及耐药检测	2018
1237	DOI:10.13590/j.cjfh.2018.02.002	2016年上海市市售肉制品中沙门菌耐药谱与分型研究	2018

1248	ISSN:1003-8507	淮安市417份腹泻患者粪便标本病原菌检测及病原学分析	2018
1262	DOI:10.3969/j.issn.2095-0381.2018.03.034	淮安市2010～2016年肉及肉制品中沙门氏菌污染状况及耐药性分析	2018
1271	ISSN:1004-8685	2016年北京市延庆区感染性腹泻病原学监测分析	2018
1281	DOI:10.16760/j.cnki.sdggws.2018.05.012	2013-2017年北京市顺义区腹泻病例中沙门菌流行特征	2018
1287	DOI:10.16406/j.pmt.issn.1672-9153.2018.07.019	2017年青岛市黄岛区医院感染性腹泻常见病原菌检测与耐药性分析	2018
1288	DOI:10.13590/j.cjfh.2018.01.005	2016年广西壮族自治区食源性沙门菌的耐药性与耐药谱研究	2018
1294	ISSN:1004-8685	2013年-2016年阿勒泰市细菌性腹泻病监测结果分析	2018
1295	ISSN:1004-8685	2015年安宁市从业人员携带沙门菌血清型及药敏分析	2018
1305	DOI:10.19567/j.cnki.1008-0414.2018.08.001	四川省规模化猪场沙门菌的分离及耐药性分析	2018
1308	DOI:10.3969/j.issn.1002-2694.2018.00.092	深圳市腹泻患者沙门菌感染状况和耐药性分析	2018
1310	DOI:10.3969/j.issn.1005-8257.2018.13.068	2016年惠州市惠阳区食品加工从业人员沙门氏菌和志贺氏菌带菌状况调查	2018
1311	DOI:10.3969/j.issn.1671-9638.2018.06.007	临床分离沙门菌的耐药性及超广谱β-内酰胺酶类耐药基因	2018
1314	DOI:10.19428/j.cnki.sjpm.2018.18418	上海市嘉定区生禽畜类食品中沙门氏菌污染情况及血清研究	2018
1324	DOI:10.11726/j.issn.1001-7658.2018.06.016	江西省餐饮从业人员沙门菌带菌状况调查	2018
1325	DOI:10.3969/j.issn.1007-5038.2018.08.004	新疆部分地区肉牛源沙门菌的分离与耐药性分析	2018
1333	DOI:10.16833/j.cnki.jbmc.2018.08.007	儿科粪便菌群构成比及耐药性分析	2018
1339	DOI:10.3969/j.issn.1008-0589.201805001	河南地区麻种鸡死胚中沙门菌的分离与耐药性分析	2018
1345	DOI:10.3969 / j.issn.1672-9455.2018.12.013	坪山区从业人员携带沙门菌的血清型分布及药敏试验分析	2018
1354	DOI:10.11843/j.issn.0366-6964.2018.02.018	广州市生鲜鸡中肠炎沙门菌的监测及耐药基因的分子	2018
1359	DOI:10.16303/j.cnki.1005-4545.2018.03.14	扬州地区部分屠宰场、农贸市场及动物医院猪源沙门菌分离、耐药性分析及PFGE分型	2018
1362	DOI:10.3969/j.issn.1001-8689.2018.09.004	北京地区腹泻病原菌流行病学及耐药特征和检测技术研究	2018
1371	DOI:10.16286/j.1003-5052.2018.04.022	潍坊市2011-2017年食品中沙门菌污染状况调查	2018
1387	DOI:10.13304/j.nykjdb.2017.0064	新疆乌鲁木齐市周边鸡场鸡源沙门氏菌耐药性及耐药基因的检测	2017
1389	ISSN:1003-8507	北京市肠道门诊腹泻儿童沙门菌感染状况和耐药性分析	2017
1405	ISSN:1004-8685	浙江嘉善2014年-2015年感染性腹泻监测结果分析及菌株耐药性研究	2017
1407	DOI:10.3969/j.issn.1002-2694.2017.04.009	泰安市感染性腹泻非伤寒沙门菌耐药监测及分型研究	2017
1413	ISSN:1005-944X	河南省猪源沙门氏菌的分离与血清分型	2017
1419	DOI:10.13604/j.cnki.46-1064/r.2017.07.12	深圳市福田区412例感染性腹泻患者沙门菌感染分析	2017
1428	DOI:10.3969/j.issn.1671-4695.2017.03.032	腹泻患者沙门菌血清型分布及耐药特征分析	2017
1440	ISSN:1006-4028	成都市食品和公共场所从业人员肠道沙门菌带菌状况调查分析	2017
1456	DOI:10.3969/j.issn.1672-9455.2017.10.041	2538例急性腹泻患者粪便标本细菌分布研究	2017
1458	DOI:10.3969/j.issn.1008-0425.2017.07.07	宠物犬及犬粮源沙门氏菌的耐药性分析及PFGE分型	2017
1474	DOI:10.3969/j.issn.1002-2694.2017.08.017	2015-2016年河南省食源性疾病沙门氏菌监测情况分析	2017
1482	DOI:10.3969/j.issn.1674-0270.2017.09.062	实时荧光PCR检测技术在冷冻禽畜肉类沙门氏菌快速分型检验中的应用	2017
1507	DOI:10.11726/j.issn.1001-7658.2017.09.017	江西省食品中沙门菌污染与耐药性调查	2017
1533	ISSN:1003-6245	西安市沙门氏菌病原学检测及药敏试验结果分析	2017
1554	ISSN:1006-4028	2013-2015年北京市顺义区947份感染性腹泻病原菌监测结果分析	2017
1567	DOI:10.3969/j.issn.1002-2376.2017.23.033	感染性腹泻标本中沙门菌与志贺菌的检测结果分析	2017
1579	DOI:10.19568/j.cnki.23-1318.2017.03.007	黑龙江省生鸡肉中沙门菌污染调查和耐药分析	2017
1587	DOI:10.14170/j.cnki.cn31-1278/s.2017.05.013	上海市活禽交易市场鸡群沙门氏菌的分离与耐药性分析	2017
1598	DOI:10.16506/j.1009-6639.2017.01.009	2014年温州市317例食源性疾病病例流行病学调查研究	2017
1620	DOI:10.3969/j.issn.1671-8348.2017.16.026	2012-2015年成都铁路辖区从业人员沙门菌携带情况分析	2017
1628	ISSN:1003-8507	2015-2016年中山市感染性腹泻病原学监测结果分析	2017
1634	ISSN:1004-8685	2014年-2016年丽水市食源性致病菌监测结果分析	2017
1639	ISSN:1005-5320	从业人员肠道沙门菌检测结果分析及PCR技术的应用	2017
1648	DOI:10.13407/j.cnki.jpp.1672-108X.2017.02.013	单中心儿童肠道门诊大便细菌及药敏分析	2017
1664	ISSN:1672-2078	吉林省规模化猪场沙门菌携带情况调查	2017
1671	DOI:10.13329/j.cnki.zyyjk.2017.0499	2015年北京市昌平区沙门菌临床菌株分子特征及耐药性	2017
1691	ISSN:1007-2705	福建省食品中沙门菌的分布特征	2017
1696	ISSN:1003-8507	2012-2016年北京市昌平区感染性腹泻病原菌检测结果分析	2017
1704	DOI:10.14163/j.cnki.11-5547/r.2017.10.003	小儿感染性腹泻流行特征、病原及耐药性分析	2017
1706	ISSN:1004-8685	浙江省宁海县2015年-2016年食源性疾病监测结果分析	2017
1709	DOI:10.13590/j.cjfh.2017.01.022	2014年金华市食源性疾病监测结果分析	2017
1734	DOI:10.13304/j.nykjdb.2017.0064	新疆乌鲁木齐市周边鸡场鸡源沙门氏菌耐药性及耐药基因的检测	2017

1754	ISSN:1004-8685	浙江嘉善2014年-2015年感染性腹泻监测结果分析及菌株耐药性研究	2017
1761	ISSN:1005-944X	河南省猪源沙门氏菌的分离与血清分型	2017
1765	DOI:10.3969/j.issn.1002-2694.2017.04.017	无锡市腹泻病人沙门菌的病原学特征及分子分型研究	2017
1767	DOI:10.13604/j.cnki.46-1064/r.2017.07.12	深圳市福田区412例感染性腹泻患者沙门菌感染分析	2017
1777	DOI:10.13515/j.cnki.hnjpm.1006-8414.2017.09.029	肉鸡养殖和屠宰加工过程中沙门氏菌污染状况及血清型分析	2017
1791	ISSN:1006-4028	成都市食品和公共场所从业人员肠道沙门菌带菌状况调查分析	2017
1803	ISSN:1004-8685	北京市密云地区腹泻病原菌耐药性分析	2017
1826	DOI:10.3969/j.issn.1002-2694.2017.08.017	2015-2016年河南省食源性疾病沙门氏菌监测情况分析	2017
1849	DOI:10.13329/j.cnki.zyyjk.2017.0835	2015-2016年自贡市食源性疾病主动监测结果	2017
1920	DOI:10.13517/j.cnki.ccm.2016.12.046	2013-2015年康平县沙门菌感染的流行病学调查及耐药性分析	2016
1938	ISSN:1004-8685	张家港市食源性疾病哨点医院监测结果分析	2016
1946	DOI:10.3969/j.issn.1007-614x.2016.24.77	乌鲁木齐天山区急性腹泻患者志贺菌、沙门菌监测结果分析	2016
1956	DOI:10.13590/j.cjfh.2016.06.023	上海市浦东新区生鲜食品中食源性致病菌监测	2016
1986	DOI:10.3969/j.issn.1672-9455.2016.17.024	阳江市人民医院283株沙门菌结果分析	2016
1987	ISSN:1004-8456	2014年金华市食源性疾病主动监测结果分析	2016
1997	DOI:10.3969/j.issn.1672-3619.2016.11.031	2014 - 2015年深圳市龙华新区感染性腹泻病原体监测结果分析	2016
2000	ISSN:1003-8507	淮安市233份畜禽肉及152份淡水龙虾中致病菌分布状况及耐药性分析	2016
2002	DOI:10.13604/j.cnki.46-1064/r.2016.05.18	上海市长宁区中外籍沙门菌感染患者流行病学特征比较	2016
2005	DOI:10.14164/j.cnki.cn11-5581/r.2016.01.131	儿童腹泻患者沙门菌感染血清型分布及耐药性分析	2016
2013	DOI:10.3969/j.issn.1671-7414.2016.03.040	2013～2014年深圳市腹泻疾病的病原学分析研究	2016
2014	ISSN:1004-8685	2011年-2014年宁波地区腹泻患者中食源性病原菌检测与流行特征分析	2016
2021	ISSN:1004-8685	深圳市宝安区2013年-2015年感染性腹泻病原菌监测结果分析	2016
2029	DOI:10.3969/j.issn.1673-8640.2016.011.006	2014年无锡市感染性腹泻的病原学分析	2016
2061	DOI:10.13590/j.cjfh.2016.04.016	黑龙江省肉鸡养殖和屠宰加工环节中沙门菌调查	2016
2065	ISSN:1004-8685	2014年-2015年余姚市沙门菌菌型分布及其药敏分析	2016
2078	DOI:10.3969/j.issn.1006-3110.2016.08.030	2013-2015年湘潭市食品中金黄色葡萄球菌和沙门菌的污染状况及耐药性分析	2016
2086	DOI:10.3969/j.issn.1006-3110.2016.02.029	2014年上海市宝山区腹泻病监测结果分析	2016
2091	DOI:10.3969/j.issn.1000-3606.2016.04.002	儿童沙门菌感染临床特点及耐药模式	2016
2097	ISSN:1004-8685	2014年北京市房山区沙门菌血清及耐药性分析	2016
2115	ISSN:1004-8685	宁波市鄞州区感染性腹泻细菌病原学监测与分析	2016
2133	DOI:10.11816/cn.ni.2016-160548	2011 - 2013年上海西区哨点医院成人急性腹泻细菌感染的病原学分析	2016
2134	DOI:10.16281/j.cnki.jocml.2016.20.061	儿童常见肠道感染疾病临床特征及耐药分析	2016
2138	DOI:10.3969/j.issn.1006-3110.2016.03.039	2011-2013年阳江市沙门菌监测结果分析	2016
2140	DOI:10.11726/j.issn.1001-7658.2016.11.020	上海市奉贤区感染性腹泻病原菌分布特点	2016
2149	ISSN:1004-8685	2013年-2014年浙江省仙居腹泻病沙门菌监测结果分析	2016
2161	ISSN:0529-6005	四川地区猪沙门菌的分离及其耐药性分析	2016
2165	ISSN:1004-8685	2010年-2014年深圳市龙岗区感染性腹泻病原谱监测结果分析	2016
2173	DOI:10.13329/j.cnki.zyyjk.2016.0062	腹泻病监测中非伤寒沙门菌血清型及耐药性分析	2016
2183	ISSN:0529-5130	南宁地区生猪屠宰环节沙门菌分离株毒力基因检测与耐药性分析	2016
2190	DOI:10.13215/j.cnki.jbyfkztb.1605035	2013-2015年白银市食源性疾病监测分析	2016
2191	DOI:10.3969/j.issn.1672-9455.2016.01.002	内江地区2012~2014年食源性沙门菌监测及耐药分析	2016
2201	ISSN:1003-8507	厦门地区156株儿童感染性腹泻沙门菌的血清分型及耐药性分析	2016
2203	DOI:10.13350/j.cjpb.161215	771例婴幼儿腹泻病原微生物监测分析	2016
2224	DOI:10.13590/j.cjfh.2016.06.023	上海市浦东新区生鲜食品中食源性致病菌监测	2016
2254	DOI:10.3969/j.issn.1672-9455.2016.17.024	阳江市人民医院283株沙门菌结果分析	2016
2265	DOI:10.3969/j.issn.1672-3619.2016.11.031	2014 - 2015年深圳市龙华新区感染性腹泻病原体监测结果分析	2016
2268	ISSN:1003-8507	淮安市233份畜禽肉及152份淡水龙虾中致病菌分布状况及耐药性分析	2016
2270	DOI:10.13604/j.cnki.46-1064/r.2016.05.18	上海市长宁区中外籍沙门菌感染患者流行病学特征比较	2016
2281	DOI:10.3969/j.issn.1671-7414.2016.03.040	2013～2014年深圳市腹泻疾病的病原学分析研究	2016
2282	ISSN:1004-8685	2011年-2014年宁波地区腹泻患者中食源性病原菌检测与流行特征分析	2016
2289	ISSN:1004-8685	深圳市宝安区2013年-2015年感染性腹泻病原菌监测结果分析	2016
2329	DOI:10.13590/j.cjfh.2016.04.016	黑龙江省肉鸡养殖和屠宰加工环节中沙门菌调查	2016
2333	ISSN:1004-8685	2014年-2015年余姚市沙门菌菌型分布及其药敏分析	2016
2346	DOI:10.3969/j.issn.1006-3110.2016.08.030	2013-2015年湘潭市食品中金黄色葡萄球菌和沙门菌的污染状况及耐药性分析	2016

2354	DOI:10.3969/j.issn.1006-3110.2016.02.029	2014年上海市宝山区腹泻病监测结果分析	2016
2359	DOI:10.3969/j.issn.1000-3606.2016.04.002	儿童沙门菌感染临床特点及耐药模式	2016
2365	ISSN:1004-8685	2014年北京市房山区沙门菌血清及耐药性分析	2016
2402	DOI:10.16281/j.cnki.jocml.2016.20.061	儿童常见肠道感染疾病临床特征及耐药分析	2016
2406	DOI:10.3969/j.issn.1006-3110.2016.03.039	2011-2013年阳江市沙门菌监测结果分析	2016
2408	DOI:10.11726/j.issn.1001-7658.2016.11.020	上海市奉贤区感染性腹泻病原菌分布特点	2016
2417	ISSN:1004-8685	2013年-2014年浙江省仙居腹泻病沙门菌监测结果分析	2016
2423	DOI:10.3969/j.issn.1671-9638.2016.11.003	深圳市儿童医院儿童粪便分离的459株沙门菌分布特征及耐药性	2016
2429	ISSN:0529-6005	四川地区猪沙门菌的分离及其耐药性分析	2016
2441	DOI:10.13329/j.cnki.zyyjk.2016.0062	腹泻病监测中非伤寒沙门菌血清型及耐药性分析	2016
2445	ISSN:1004-8685	2013年-2014年北京市房山区食源性疾病监测结果分析	2016
2448	DOI:10.3969/j.issn.1673-4130.2016.19.017	某儿童医院感染病房病原菌分布及耐药性分析	2016
2454	ISSN:1003-8507	2015年泰州市食源性疾病病原微生物监测结果分析	2016
2469	ISSN:1003-8507	厦门地区156株儿童感染性腹泻沙门菌的血清分型及耐药性分析	2016
2490	ISSN:1004-8685	2013年-2014年杭州市西湖区食源性疾病哨点医院监测结果分析	2015
2496	DOI:10.19812/j.cnki.jfsq11-5956/ts.2015.09.035	乌鲁木齐市零售牛羊肉中沙门氏菌的调查	2015
2499	DOI:10.3969/j.issn.0529-6005.2015.12.030	山东地区生猪屠宰环节沙门菌毒力基因携带状况分析	2015
2500	ISSN:1672-3619	2010-2013年深圳市龙岗区沙门菌分子流行病学分析	2015
2504	DOI:10.16406/j.pmt.issn.1672-9153.2015.09.006	潍坊市肉鸡孵化养殖和屠宰销售环节沙门菌污染状况调查	2015
2508	DOI:10.3969/j.issn.1673-4130.2015.12.019	544例急性腹泻门诊病例粪便的细菌菌谱及药敏分析	2015
2526	DOI:10.13604/j.cnki.46-1064/r.2015.07.18	深圳市南山区2013-2014年感染性腹泻病原体监测结果	2015
2531	DOI:10.3969/j.issn.1001-8689.2015.02.012	水产品中沙门菌的污染情况调查和耐药性分析	2015
2544	DOI:10.3969/j.issn.1671-2587.2015.01.005	感染性腹泻中沙门菌血清型及耐药性分析	2015
2560	DOI:10.11816/cn.ni.2015-144147	感染性腹泻患者病原菌分布与耐药性研究	2015
2575	DOI:10.3784/j.issn.1003-9961.2015.01.009	2010-2013年广东省深圳市南山区腹泻患者沙门菌血清和脉冲场凝胶电泳分析	2015
2578	ISSN:1004-8685	北京市密云县腹泻患者沙门菌病原学特征及耐药性分析	2015
2595	DOI:DOI:10.13329/j.cnki.zyyjk.2015.0856	2014年北京市密云县腹泻患者病原菌监测结果	2015
2596	ISSN:1003-8507	北京市海淀区食源性疾病主动监测结果分析	2015
2603	DOI:DOI:10.16656/j.issn.1673-4696.2015.05.019	四川省2009-2014年鸡源沙门菌耐药性变迁的调查	2015
2605	DOI:10.13590/j.cjfh.2015.05.018	宁波地区食品中致病菌监测与流行株分析	2015
2623	DOI:10.3969/j.issn.0258-7033.2015.06.016	猪肉生产链细菌耐药性及其耐药基因调查研究	2015
2627	DOI:DOI:10.13881/j.cnki.hljxmsy.2015.1437	市售鲜畜肉中沙门菌的血清型及耐药性研究	2015
2632	DOI:10.3877/cma.j.issn.1674-0785.2015.13.018	2013年东莞市沙门菌感染流行病学特征及耐药性分析	2015
2652	ISSN:1003-8507	淄博市肉鸡产业链中沙门菌污染状况及其血清型与分子分型分析	2015
2653	DOI:10.3969/j.issn.1671-9638.2015.06.001	2011-2013年北京某城区感染性腹泻患者致病菌检测分析	2015
2665	DOI:10.16431/j.cnki.1671-7236.2015.08.034	鸡源致病性沙门氏菌的分离及血清型和药物敏感性分析	2015
2684	DOI:10.16372/j.issn.1004-6364.2015.07.017	山东地区肉鸡屠宰环节沙门菌流行病学调查与毒力分析	2015
2689	DOI:DOI:10.13329/j.cnki.zyyjk.2015.0767	2012-2013年开封市肉鸡产业链沙门菌污染状况调查	2015
2706	DOI:10.3969/j.issn.1006-3110.2015.010.030	2013-2014年北京市顺义区感染性腹泻病原菌监测分析	2015
2723	DOI:DOI:10.13329/j.cnki.zyyjk.2015.0171	2012-2013年深圳市光明新区感染性腹泻病原体监测结果分析	2015
2725	DOI:10.16462/j.cnki.zhjbkz.2015.03.015	2007-2013年广东省肠炎沙门菌耐药情况分析	2015
2738	DOI:10.11984/j.issn.1000-7083.20150099	四川省猪源沙门氏菌及耐药性变迁调查	2015
2749	DOI: DOI:10.19428/j.cnki.sjpm.2015.03.003	上海市虹口区2010-2012年腹泻病例沙门菌监测及分子型特征	2015
2753	DOI:10.3760/cma.j.issn.1008-6706.2015.16.013	山西省腹泻病原菌检测分析及耐药性研究	2015
2756	ISSN:1005-5916	宁波地区腹泻病人中食源性致病菌检测与分析	2015
2761	DOI:10.3969/j.issn.0529-6005.2015.11.029	吉林省猪源沙门菌毒力基因检测及耐药性分析	2015
2768	ISSN:1000-6850	规模化养殖场猪粪源沙门氏菌的耐药性调查	2015
2773	DOI:10.3969/j.issn.1673-4130.2015.18.010	2009～2014年珠海市腹泻儿童沙门菌感染的流行病学特征	2015
2788	ISSN:1672-3619	深圳市生肉食品6种食源性致病菌污染状况调查	2015
2795	DOI:10.3760/cma.j.issn.0253-9624.2014.08.010	湖南省动物源性食品中沙门菌流行特征分析	2014
2803	ISSN:1006-4028	2007-2012年自贡市食品食源性致病菌监测分析	2014
2815	DOI:10.11816/cn.ni.2014-130196	综合医院非伤寒沙门菌感染腹泻患者病原学分析	2014
2817	ISSN:1004-8685	桐庐县感染性腹泻病原菌调查及其耐药分析	2014
2822	ISSN:1671-7236	内蒙古地区奶牛源沙门氏菌的分离、及其对小鼠的致病性研究	2014

2834	DOI:10.13590/j.cjfh.2014.06.003	江西省食源性沙门菌血清分型及脉冲场凝胶电泳指纹图谱研究	2014
2837	DOI:10.3969/cjz.j.issn.1002-2694.2014.01.004	鸡源肠炎沙门氏菌对抗菌药物的耐药性分析	2014
2851	DOI:DOI:10.16303/j.cnki.1005-4545.2014.11.015	肉鸡产业链中沙门菌流行情况及其耐药性	2014
2863	DOI:10.3969/j.issn.1009-7708.2014.06.014	72株沙门菌血清型分布及药敏试验结果分析	2014
2889	DOI:10.13590/j.cjfh.2014.06.022	市售活鸡和腹泻患者中非伤寒沙门菌分子特征和耐药性研究	2014
2893	DOI:10.3969/j.issn.1005-9369.2014.08.008	猪沙门氏菌的分离及其耐药性分析	2014
2900	ISSN:1001-0084	百色市动物饲料中沙门氏菌分离检测报告	2014
2906	ISSN:1671-7236	上海市零售禽肉制品和活禽中沙门氏菌血清型与耐药性研究	2014
2912	DOI:10.3784/j.issn.1003-9961.2014.06.004	婴幼儿腹泻感染的非伤寒沙门菌分子分型和耐药情况研究	2014
2924	DOI:10.3969/j.issn.1672-9455.2014.02.010	婴幼儿感染性腹泻的致病菌及肠道病毒监测结果分析	2014
2946	DOI:DOI:10.19813/j.cnki.weishengyanjiu.2014.06.010	2012年山东省肉鸡生产加工环节沙门菌污染水平及耐药分析	2014
2964	DOI:10.3969/j.issn.1673-4130.2014.15.035	6417例感染性腹泻患者沙门氏菌感染情况分析	2014
2965	DOI:10.3969/j.issn.1009-7708.2014.04.013	东莞市腹泻患者沙门菌同源性及耐药性特征	2014
2993	ISSN:1004-8685	石家庄2013年鸡肉沙门菌污染状况分析	2014
2994	DOI: DOI:10.16718/j.1009-7708.2014.02.010	小儿沙门菌肠炎344例病原菌分布及药敏试验结果	2014
3001	ISSN:2095-7629	对腹泻患儿进行病原学检测结果的分析及其临床意义探讨	2014
3006	DOI:DOI:10.13515/j.cnki.hnjpm.2014.06.005	龙岩市9类食品中食源性致病菌监测与分析	2014
3022	DOI: DOI:10.13604/j.cnki.46-1064/r.2014.05.001	梧州市2009～2012年食品中致病菌监测结果分析	2014
3023	ISSN:1006-4028	2007-2009年达州市全球沙门菌监测项目分析	2014
3033	DOI:10.3969/j.issn.1672-5190.2014.07.006	动物源性沙门氏菌的分离及耐药性研究	2014
3037	DOI:10.13590/j.cjfh.2014.05.022	2012年河南省市售生制速冻面米制品食源性致病菌污染状况监测	2014
3317	DOI:10.3969/j.issn.1006-3110.2013.01.005	湖南省即食食品中食源性致病菌污染状况及耐药性研究	2013
3462	DOI:10.3969/j.issn.1006-3110.2012.12.019	2010-2011年广西食品中沙门菌监测与分析	2012
3488	DOI:10.3969/j.issn.1673-4130.2012.19.057	社区细菌感染性腹泻病原学监测结果分析	2012
3534	ISSN:1004-1257	2010-2011年上海市闵行区腹泻患者病原菌检测结果	2012
3538	DOI:DOI:10.13590/j.cjfh.2012.02.027	2010年江苏省肉鸡沙门菌污染专项监测分析	2012
3542	DOI:10.3969/j.issn.1001-5949.2012.03.040	银川市市售食品中5种食源性致病菌监测结果分析	2012
3567	ISSN:1673-9388	濮阳市鼠类沙门氏菌感染状况调查分析	2012
3593	DOI:10.3969/j.issn.1674-070X.2012.10.022.048.02	腹泻患者病原菌分布和耐药性分析	2012
3604	DOI:10.3784/j.issn.1003-9961.2012.2.005	四川省成都市433例婴幼儿腹泻病原微生物监测分析	2012
3618	DOI:10.3969/j.issn.1674-1129.2012.02.006	2009年至2011年江西省食品中沙门菌污染状况调查	2012
3633	DOI:10.3969/j.issn.1003-6245.2012.03.017	2010年石家庄市食品、公共场所从业人员沙门氏菌携带情况分析	2012
3649	ISSN:1004-1257	上海市金山区2006-2010年急性腹泻患者沙门氏菌监测结果分析	2012
3651	DOI:10.3969/j.issn.1006-9070.2012.03.003	2011年宿迁市食源性致病菌监测分析	2012
3664	ISSN:1006-9070	上海市金山区2009年-2010年腹泻病人及食品中沙门菌检测结果分析	2011
3673	DOI:DOI:10.13604/j.cnki.46-1064/r.2011.02.063	云南战区腹泻病原菌监测及流行病学特征研究	2011
3675	DOI:DOI:10.13515/j.cnki.hnjpm.2011.05.032	2009-2010年睢县致泻性病原菌监测结果分析	2011
3679	DOI:10.3969/j.issn.1006-3110.2011.11.069	长沙地区儿童腹泻病原菌分布及耐药性分析	2011
3688	DOI:DOI:10.13241/j.cnki.pmb.2011.20.010	2010年上海市食源性沙门菌菌型分布和药敏分析以及快速检测方法的建立	2011
3716	ISSN:1671-7236	四川省动物性食品源沙门氏菌的耐药性监测与分析	2011
3727	ISSN:1672-3619	惠州市区腹泻病人沙门菌感染水平研究	2011
3732	ISSN:1004-8685	泰州市市售食品食源性致病菌谱及耐药状况分析	2011
3734	DOI:DOI:10.16021/j.cnki.1007-8622.2011.03.008	细菌性腹泻739例病原菌分布和耐药性分析	2011
3744	DOI:10.3969/j.issn.1673-6184.2011.03.003	2010年夏季急性细菌感染性腹泻患儿沙门菌感染分析	2011
3763	ISSN:1003-8507	商丘市2006～2010年沙门菌感染性腹泻监测结果分析	2011
3773	DOI:10.3969/j.issn.1001-8883.2011.04.008	达州市2007～2010年沙门菌监测结果分析	2011
3774	DOI: DOI:10.13329/j.cnki.zyyjk.2011.20.043	2010年北京市西城区肠道病原菌监测结果	2011
3778	ISSN:1673-9388	惠州市饮服从业人员与腹泻患者沙门氏菌感染状况调查	2011
3787	DOI:10.3969/j.issn.1673-6184.2011.03.005	72株非伤寒沙门菌药敏分析及其所致儿童肠炎的临床特点	2011
3827	DOI:10.3969/j.issn.1674-0742.2011.03.022	对芷江县饮食服务行业人群中肠道致病菌的检测与分析	2011
3837	DOI:DOI:10.16153/j.1002-7777.2011.08.023	婴幼儿腹泻沙门菌分型与耐药机制分析	2011
3842	DOI:DOI:10.13381/j.cnki.cjm.2011.07.008	合肥地区鸡沙门菌带菌情况调查及其血清型与基因型分析	2011
3856	ISSN:1002-6630	2007—2008年西安地区鸡肉源沙门氏菌相关特性分析	2011
3867	DOI:10.3969/j.issn.1003-6245.2011.08.017	泰州市食品、公共场所从业人员沙门氏菌携带状况及耐药谱变化的研究	2011

3870	DOI:10.3969/j.issn.1671-8194.2011.33.293	某区食品中沙门菌的监测与分析	2011
3878	ISSN:1672-3619	2008-2010年江门市腹泻病原菌现状调查	2011
3887	DOI:10.3969/j.issn.1007-8134.2011.02.007	乌鲁木齐地区2010年腹泻病原菌耐药性监测与分析	2011
3913	DOI:DOI:10.16766/j.cnki.issn.1674-4152.2010.04.022	腹泻患者细菌分离及药敏480例分析	2010
3918	DOI:10.3969/j.issn.1672-9455.2010.09.036	钦州地区儿童细菌感染性腹泻的病原菌及药敏试验分析	2010
3919	DOI:10.3969/j.issn.1006-7159.2010.03.029	江西口岸饮食和公共场所从业人员肠道沙门氏菌检测结果分析	2010
3935	DOI:DOI:10.13329/j.cnki.zyyjk.2010.23.031	2004-2009年上海市金山区饮食和服务行业从业人员携带沙门菌检测结果	2010
3937	ISSN:1004-8685	2005年-2008年黑龙江省食品中沙门菌污染监测分析	2010
3938	DOI:10.3969/j.issn.1002-2694.2010.09.026	规模猪场健康猪沙门菌带菌情况调查	2010
3942	ISSN:1004-8685	生肉制品中沙门菌污染状况及耐药性分析	2010
3956	DOI:DOI:10.19568/j.cnki.23-1318.2010.02.026	2003～2008年山东省流通领域食品沙门氏菌污染状况调查	2010
3964	ISSN:1004-8685	广西食源性沙门菌主动监测网建立及其结果分析	2010
3972	DOI: DOI:10.16372/j.issn.1004-6364.2010.17.002	河南省鸡源沙门菌新近流行株的分离及其耐药性分析	2010
3975	DOI:10.3969/j.issn.1673-7555.2010.36.111	2009年闵行区肠道门诊腹泻患者肛拭病原菌检测结果分析	2010
3982	ISSN:1004-8685	广安市6类食品中食源性致病菌污染调查报告	2010
3996	ISSN:1004-8685	2008年江苏省食源性致病菌监测分析	2010
4010	DOI:10.3969/j.issn.1002-2694.2010.12.013	河南省鹤壁市食源性和禽源性沙门氏菌耐药性研究	2010
4011	DOI:10.3969/j.issn.1004-3594.2010.06.009	兰州地区683例细菌性腹泻病原菌分布和耐药性分析	2010
4023	DOI: DOI:10.13604/j.cnki.46-1064/r.2010.06.013	佛山市食品中食源性致病菌污染状况监测	2010
4035	DOI:10.3969/j.issn.1002-2481.2010.09.18	山西省鸡沙门氏菌病流行病学调查分析	2010
4038	ISSN:1004-8685	陕西省市售肉与肉制食品中食源性致病菌污染监测与分析	2010
4044	DOI: DOI:10.13343/j.cnki.wsxb.2010.06.017	陕西食源性沙门氏菌耐药及相关基因	2010
4047	DOI:10.3784/j.issn.1003-9961.2010.11.015	2007-2009年浙江省余姚市感染性腹泻沙门菌监测结果分析	2010
4057	ISSN:1003-8280	濮阳市鼠类肠道寄生虫及沙门菌感染状况调查	2010
4067	ISSN:1007-1040	南通市饮服从业人员肠道沙门氏菌携带情况调查	2010
4088	DOI:10.3969/j.issn.1007-614x.2010.19.168	腹泻儿童大便沙门菌及药敏分析	2010
4091	ISSN:1001-0580	上海市黄浦区饮服从业人员沙门菌监测分析	2010
4094	DOI:10.3969/j.issn.1006-9070.2010.04.001	江苏地区2008年～2009年食源性致病菌监测研究	2010
4105	DOI:10.3969/j.issn.1000-3606.2010.06.008	北京地区儿童感染性腹泻病原学和耐药性分析	2010
4121	DOI:10.3969/j.issn.1006-9070.2010.03.006	南通市市售食品沙门菌菌型分布及耐药性研究	2010
4126	DOI:10.3321/j.issn:1005-4529.2009.21.072	腹泻病原菌的分离与	2009
4147	DOI:10.3784/j.issn.1003-9961.2009.05.014	2008年夏季北京市沙门菌腹泻监测结果分析	2009
4159	DOI:10.3969/j.issn.1673-758X.2009.01.014	2007年梧州市从业人员肠道沙门氏菌检测结果分析	2009
4163	DOI:10.3969/j.issn.1673-6184.2009.02.008	2007年上海市儿童社区获得性腹泻致病菌谱分析	2009
4169	ISSN:1004-8685	2001～2008年三门县从业人员粪便沙门菌检测结果分析	2009
4191	ISSN:1004-8685	舟山海岛儿童细菌性腹泻病原菌分布及耐药分析	2009
4199	DOI:10.3969/j.issn.1008-1089.2009.08.015	成人感染性腹泻病原学分类与临床分析	2009
4206	ISSN:1004-8685	2006～2007年河南省生肉食品中沙门菌的主动监测及其DNA指纹图谱库的建立	2009
4210	ISSN:1004-8685	下城区饮食和公共场所从业人员肠道沙门菌携带状况调查分析	2009
4230	ISSN:1672-4208	社区环境中沙门菌特性研究	2009
4232	DOI:10.3784/j.issn.1003-9961.2009.05.006	2007年重庆市人群沙门菌监测分析	2009
4239	DOI:10.3969/j.issn.1007-0931.2009.01.001	715株感染性腹泻病原菌分布及耐药状况	2009
4245	ISSN:1671-5039	2005-2008年深圳市盐田区食品中常见食源性致病菌监测结果分析	2009
4246	ISSN:1009-9727	江门市区2004～2009年食源性致病菌监测研究	2009
4254	ISSN:1001-4411	2007年儿童急性感染性腹泻病原菌监测结果分析	2009
4261	DOI:DOI:10.13590/j.ejfh.2009.02.005	2007年江苏省食源性致病菌监测分析	2009
4262	ISSN:1004-8685	陕西省市售熟肉制品食源性致病菌污染状况主动监测结果分析	2009
4272	ISSN:1004-8685	食品、公共场所从业人员沙门菌带菌检测分析	2009
4312	ISSN:1005-2224	儿童细菌性腹泻病原菌10年变迁与药敏分析	2009
4315	ISSN:1004-1257	上海市奉贤区服务行业从业人员肠道致病菌检测结果分析	2009
4318	DOI:DOI:10.19568/j.cnki.23-1318.2009.01.060	山东省2003～2007年食源性致病菌监测结果分析	2009
4347	ISSN:1004-8685	许昌市食品食源性致病菌检测	2009
4352	DOI:10.3784/j.issn.1003-9961.2009.12.007	2006-2008年上海市闵行区腹泻病病原菌的流行特征	2009
4361	DOI:10.3969/j.issn.1001-0025.2009.04.007	192株志贺菌属和76株沙门菌属菌群菌型分布及耐药性分析	2009

4374	DOI:10.3321/j.issn:0254-6450.2008.12.007	广东省2007年度非伤寒沙门菌监测及病原学特征分析	2008
4378	DOI:10.3969/j.issn.1671-5039.2008.06.025	广州市荔湾区饮食及公共场所服务行业从业人员沙门菌带菌调查	2008
4380	DOI:10.3969/j.issn.1006-3110.2008.05.016	河北省2005-2007年沙门菌主动监测及基因分型结果	2008
4387	DOI:10.3969/j.issn.1672-9455.2008.23.014	2007年自贡市食源性沙门菌菌型分布及药敏结果分析	2008
4391	DOI:10.3969/j.issn.1004-8685.2008.04.048	服务行业从业人员肠道致病菌检测结果分析	2008
4402	DOI:10.3969/j.issn.1673-5234.2008.01.027	上海口岸饮食和公共场所从业人员肠道沙门氏菌调查分析	2008
4409	DOI:10.3969/j.issn.1004-9231.2008.03.004	上海市奉贤区从业人员肠道沙门菌和志贺菌检测结果	2008
4415	DOI:10.3969/j.issn.1006-3110.2008.03.125	儿童肠道感染细菌及耐药性研究	2008
4437	ISSN:1008-3847	豫北地区规模化鸡场病原菌及耐药性趋势的调查	2008
4447	DOI:10.3969/j.issn.1673-758X.2008.01.002	2005年广西食源性沙门氏菌污染监测分析	2008
4449	DOI:10.3969/j.issn.1673-758X.2008.06.017	南宁市生食蔬菜中病原菌污染监测	2008
4466	DOI:10.3321/j.issn:1001-0580.2008.02.047	陕西省2002～2006年食源性致病菌污染状况	2008
4469	DOI:10.3969/j.issn.1004-8685.2008.02.012	河北省食源性致病菌监测网的建立及主动监测结果分析	2008
4492	DOI:10.3969/j.issn.1004-1257.2008.10.029	福州市2006年食品行业从业人员沙门菌检出情况及其耐药性	2008
4535	DOI:10.3969/j.issn.1006-3617.2008.03.006	上海市沙门菌病流行特征分析	2008
4641	DOI:10.3969/j.issn.1672-3619.2007.04.008	武汉社区腹泻病人粪便中致病菌的构成与耐药性研究	2007
4655	DOI:10.3969/j.issn.1004-8456.2007.03.017	丹东口岸2003-2005年进口海产品中3种致病菌的检验	2007
4666	DOI:10.3969/j.issn.1004-8685.2007.07.016	合肥市屠宰生猪主要微生物学指标调查研究	2007
4676	DOI:10.3969/j.issn.1004-8685.2007.09.060	宁波市江北区食源性致病菌监测结果分析	2007
4688	DOI:10.3969/j.issn.1004-8685.2007.09.059	舟山海岛腹泻病人的病原菌监测及致泻性大肠埃希菌药敏试验	2007
4690	DOI:10.3969/j.issn.1007-2705.2007.06.025	济宁市食品从业人员肠道沙门菌带菌调查	2007
4694	DOI:10.3969/j.issn.1004-1257.2007.15.025	临沂市感染性腹泻病原菌调查分析	2007
4707	DOI:10.3969/j.issn.0529-6005.2007.01.026	猪/胴/体/沙/门/氏/菌/污/染/的/调/查	2007
4740	DOI:10.3969/j.issn.1004-9231.2007.05.008	上海市卢湾区健康从业人员沙门菌感染菌型及耐药性分析	2007
4754	DOI:10.3969/j.issn.1004-8685.2007.03.057	生肉制品中沙门菌、单核细胞增生李斯特菌及金黄色葡萄球菌流行特征研究	2007
4771	DOI:10.3969/j.issn.1004-8685.2006.03.040	河北省食品中沙门菌污染状况调查分析	2006
4787	DOI:10.3969/j.issn.1004-8685.2006.09.039	食品中沙门菌污染状况及耐药性研究	2006
4797	DOI:10.3969/j.issn.1006-3110.2006.05.070	2002～2005年广西食品中沙门氏菌的监测与分析	2006
4833	DOI:10.3969/j.issn.1003-8507.2006.10.007	河北省食品中沙门氏菌的耐药性研究	2006
4848	DOI:10.3969/j.issn.1004-8456.2006.03.003	免疫磁珠法检测食品中的沙门菌及分离菌株的耐药性	2006
4860	DOI:10.3969/j.issn.1674-5354.2006.05.002	东宁口岸中俄界河(瑚布图河)卫生监督调查报告	2006
4871	DOI:10.3969/j.issn.1671-5039.2006.01.035	广州市芳村区食品从业人员沙门菌带菌状况分析	2006
4875	DOI:10.3969/j.issn.1001-5949.2006.10.033	小儿肠道感染的致病菌及其耐药状况研究	2006
4907	DOI:10.3969/j.issn.1004-8685.2006.07.042	2005年河南省食源性致病菌和耐药性监测	2006
4914	DOI:10.3969/j.issn.1004-8456.2006.01.006	扬州市食品中7种食源性致病菌污染状况及耐药性研究	2006
4926	DOI:10.3969/j.issn.1672-9153.2006.01.047	1998～2003年广州铁路辖区部分从业人员沙门菌携带情况分析	2006
4936	DOI:10.3969/j.issn.1672-1721.2006.08.017	小儿感染性腹泻452例临床和病原学分析	2006
5125	DOI:10.3969/j.issn.1005-376X.2005.05.017	儿童感染性腹泻病原学研究	2005
5151	DOI:10.3969/j.issn.1006-4028.2004.04.043	个体餐饮及公共场所从业人员沙门菌的感染情况	2004
5157	DOI:10.3969/j.issn.1004-9231.2004.09.009	上海市闵行区饮食和公共场所从业人员沙门和志贺菌属带菌情况	2004
5158	DOI:10.3969/j.issn.1006-3110.2004.05.059	2000～2002年龙岩市食源性致病菌监测分析	2004
5184	DOI:10.3969/j.issn.1004-8685.2004.04.055	扬州市2002-2003食源性致病菌监测研究	2004
5368	DOI:10.3969/j.issn.1003-8507.2003.04.028	成都市从业人员肠道带菌调查分析	2003
5370	DOI:10.3969/j.issn.1000-2200.2003.04.044	服务从业人员沙门菌带菌菌型分布及药敏试验	2003
5375	DOI:10.3969/j.issn.1004-8685.2003.04.006	南昌市西湖区从业人员携带的沙门菌血清分型及药敏试验	2003
5376	DOI:10.3969/j.issn.1004-8456.2003.05.005	福建省2000年～2002年食品中沙门氏菌的监测与分析	2003
5384	DOI:10.3969/j.issn.1009-9727.2003.04.060	广州市东山区有关从业人员中检出沙门氏菌的药敏分析	2003
5394	ISSN:1001-7062	小儿感染性腹泻病685例病因分析	2003
5396	DOI:10.3969/j.issn.1004-8456.2003.06.003	2002年陕西省食品中食源性致病菌监测	2003
5397	DOI:10.3969/j.issn.1004-8685.2003.05.014	北京市食品中五种食源性致病菌污染状况调查研究	2003
5400	DOI:10.3969/j.issn.1004-8685.2002.06.055	1999年-2001年常熟市从业人员肠道沙门菌检测结果	2002
5413	DOI:10.3969/j.issn.1671-5039.2002.03.013	广州市东山区饮食及公共场所服务行业人员携带沙门氏菌的分析	2002
5414	DOI:10.3969/j.issn.1004-1257.2002.07.044	饮食服务业人员沙门氏菌携带情况调查及分析	2002
5474	DOI:10.3969/j.issn.1006-9070.2002.03.019	江苏省5类食品中沙门菌污染的调查	2002

5510	DOI:10.3969/j.issn.1000-7377.2002.03.002	感染性腹泻病2 489例病因分析	2002
5518	DOI:10.3969/j.issn.1077-6131.2001.03.005	公共场所及饮食从业人员沙门氏菌带菌状况调查及菌型分析	2001
5520	DOI:10.3969/j.issn.0529-6005.2001.09.011	内蒙古地区鸡沙门氏菌病流行病学调查	2001
5623	DOI:10.3969/j.issn.1003-8507.2000.03.025	广州市天河区食品从业人员肠道沙门氏菌带菌调查分析及防治对策探讨	2000
5625	DOI:10.3969/j.issn.1003-8507.2000.02.071	自贡地区猪、鱼、鸭、兔等带染沙门氏菌的调查	2000
5627	DOI:10.3969/j.issn.1674-3679.1999.02.033	67 885名饮、服行业人员沙门菌带菌调查	2000
5664	ISSN:1002-2694	鼠伤寒沙门氏菌肠道感染59例临床与实验研究	1995
5665	ISSN:1002-2694	1989～1993年深圳市748株沙门氏菌的菌型分布	1995
5684	ISSN:1007-2705	福州市从人员沙门氏菌菌型分布及耐药性调查	2000
5705	ISSN:1007-1040	马鞍山市食品、公共场所从业人员沙门氏菌感染情况调查	1999
5706	ISSN:1004-8685	屠猪带染沙门氏菌调查分析	1999
5710	ISSN:1003-8507	成都市食品从业人员肠道沙门氏菌属与志贺氏菌属的带菌调查分析	1999
5743	ISSN:1008-0619	家禽屠宰场沙门氏菌分离株的血清型和药物感受性	1998
5759	DOI:DOI:10.19428/jcnki.sjpm.1997.05.011	健康人群沙门氏菌属和志贺氏菌属带菌情况分析	1997
5827	ISSN:1009-9158	167例沙门菌性腹泻菌型分布和药敏试验	1996
5849	ISSN:1671-5039	饮食从业人员健康体检粪便中检出504株沙门氏菌菌型分布	1995
5866	ISSN:1005-944X	山西12市五种肉品沙门氏菌检验情况	1994
5877	ISSN:2096-5087	临安县健康人群携带沙门氏菌的菌谱和动态	1994
5878	ISSN:1004-0188	西藏地区沙门氏菌属的PCR检测及病原体耐药性研究	1994
5892	ISSN:1004-9770	从饮食从业人员的粪便中检出5株沙门氏菌	1994
5916	ISSN:1002-2694	赣湘边境地区蛇类检出沙门氏菌型分布	1993
5937	ISSN:1001-0580	猪鸭沙门氏菌带菌调查	1993
5944	ISSN:1001-0580	广州市天河区健康体检检出的382株沙门氏菌菌型分布	1993
5956	ISSN:1003-7950	西宁市农贸市场鲜猪肉沙门氏菌污染调查	1992
6174	ISSN:1005-4529	我院小儿科散发性鼠伤寒沙门氏菌医院感染调查	1999
6203	ISSN:1004-8685	感染性腹泻患者病原学监测结果报告	1997
6211	ISSN:1004-8685	饮食服务行业从业人员肠道致病菌带菌调查	1996
6225	DOI:10.3760/cma.j.issn.0254-6450.1996.05.108	豫东睢县住院腹泻患者致泻病原学研究	1996
6247	ISSN:1008-1070	我国中原地区农村腹泻病病原谱及流行病学分析	1993
6252	ISSN:2095-1116	小儿感染性腹泻常见病原菌研究	1993
6254	ISSN:1003-4315	鸡源肠道杆菌的分离与鉴定	1993
6260	ISSN:1671-5039	广州市芳村区1990~1991年食品从业人员带菌状况调查	1992
6264	ISSN:2096-5087	富阳县22914名"健康人"肠道致病菌带菌调查	1992
6279	ISSN:2096-5087	温州市食品行业从业人员健康体检	1990
6308	ISSN:2096-5532	511例小儿夏季腹泻病原学分析	1989
6326	ISSN:1004-8456	农贸市场肉品沙门氏菌污染情况调查	1989
6336	ISSN:2097-2717	豫东平原睢县城乡腹泻病原学的调查研究	1989
6346	ISSN:0253-9624	741株沙门氏菌的菌型调查	1987
6393	ISSN:1008-7044	1985-1987年蚌埠市饮食服务从业人员29, 549人沙门氏菌属带菌调查报告	1989
6430	DOI:10.3760/cma.j.issn.0254-6450.1985.06.106	怀化市435例急性腹泻患者的病原菌分析及耐药性调查	1985
6598	ISSN: 1682-6485	中部地区产蛋鸡与带壳蛋之沙门氏菌监测调查	2010
6609	ISSN: 1682-6485	台湾中部地区流浪犬及家犬沙门氏菌之调查	2008
6669	DOI:DOI:10.19485/j.cnki.issn2096-5087.2021.11.024	柯桥区食源性疾病致病菌监测结果	2021
6673	DOI: DOI:10.19485/j.cnki.issn2096-5087.2021.11.024	2015—2019年湖南省食源性沙门菌流行病学特征分析	2021
6685	ISSN:1008-0589	2019年～2020年河北地区鸡源沙门菌流行病学调查及耐药性分析	2021
6712	DOI:10.1016/j.ijfoodmicro.2020.108640	Detection of chicken carcasses contaminated with Salmonella enterica serovar in the abattoir environment of Taiwan	2020
6733	DOI:10.3382/ps/pex293	Comparison of prevalence, phenotype, and antimicrobial resistance of Salmonella serovars isolated from turkeys in Taiwan	2018
6788	DOI:10.1637/9420-060910-Reg.1	Emergence of Salmonella enterica serovar Potsdam as a major serovar in waterfowl hatcheries and chicken eggs	2011
6791	DOI:10.4315/0362-028X.JFP-10-394	Transmission of Salmonella between swine farms by the housefly (Musca domestica)	2011
6806	DOI:10.1186/1471-2180-10-86	Characterization of 13 multi-drug resistant Salmonella serovars from different broiler chickens associated with those of human isolates	2010
6808	DOI:10.1177/104063871002200107	Prevalence and antimicrobial susceptibility of salmonellae isolates from reptiles in Taiwan	2010
6837	DOI:10.1007/s11259-007-0009-4	Salmonellae and campylobacters in household and stray dogs in northern Taiwan	2007
6877	DOI:10.1292/jvms.67.7	The prevalence and antimicrobial susceptibilities of Salmonella and Campylobacter in ducks in Taiwan	2005
6990	DOI:10.1016/j.fm.2014.04.010	IncreAISng prevalence of hydrogen sulfide negative Salmonella in retail meats	2014

7039	DOI:10.1017/s002217240005614x	Salmonella in pig carcasses for human consumption in Hong Kong: a study on the mode of contamination	1977
7043	DOI:10.1017/s0022172400045654	Human infection with Salmonella choleraesuis in Hong Kong	1967
7063	DOI:10.3389/fmicb.2021.684400	Genomic Investigation of Antimicrobial-Resistant Salmonella enterica Isolates From Dead Chick Embryos in China	2021
7080	DOI:10.1089/fpd.2021.0018	Prevalence and Characteristics of Salmonella spp. from a Pig Farm in Shanghai, China	2021
7085	DOI:10.1016/j.ijfoodmicro.2021.109246	Tracking Salmonella enterica by whole genome sequencing of isolates recovered from broiler chickens in a poultry production system	2021
7091	DOI:10.1089/mdr.2020.0585	Prevalence and Antimicrobial Resistance of Salmonella and Staphylococcus aureus in Fattening Pigs in Hubei Province, China	2021
7093	DOI:10.3389/fmicb.2021.636332	Genomic Characterization of Salmonella enterica Isolates From Retail Meat in Beijing, China	2021
7094	DOI:10.1186/s13568-021-01221-9	Subtyping Salmonella isolated from pet dogs with multilocus sequence typing (MLST) and clustered regularly interspaced short palindromic repeats (CRISPRs)	2021
7098	DOI:10.3389/fvets.2021.607491	Characteristics of Salmonella From Chinese Native Chicken Breeds Fed on Conventional or Antibiotic-Free Diets	2021
7101	DOI:10.3389/fvets.2021.581946	Prevalence and Antimicrobial Resistance of Salmonella Isolated From Dead-in-Shell Chicken Embryos in Shandong, China	2021
7110	DOI:10.1016/j.psj.2020.09.079	Prevalence and antimicrobial resistance of Salmonella enterica subspecies enterica serovar Enteritidis isolated from broiler chickens in Shandong Province, China, 2013-2018	2021
7113	DOI:10.1089/fpd.2020.2881	Antimicrobial Resistance and Molecular Characterization of Class 1 Integron in Salmonella Isolates Recovered from Pig Farms in Chongqing, China	2021
7125	DOI:10.1128/mSystems.01234-20	Evolution of Ciprofloxacin Resistance-Encoding Genetic Elements in Salmonella	2020
7127	DOI:10.1016/j.psj.2020.09.071	Characterization of integrons and antimicrobial resistance in Salmonella from broilers in Shandong, China	2020
7145	DOI:10.3389/fmicb.2020.01482	Prevalence, Serotype, Antibiotic Susceptibility, and Genotype of Salmonella in Eggs From Poultry Farms and Marketplaces in Yangling, Shaanxi Province, China	2020
7173	DOI:10.3390/microorganisms8030444	Prevalence, Antimicrobial Resistance, Virulence Genes and Genetic Diversity of Salmonella Isolated from Retail Duck Meat in Southern China	2020
7177	DOI:10.1089/fpd.2019.2737	Prevalence and Characterization of Salmonella in Three Typical Commercial Pig Abattoirs in Wuhan, China	2020
7185	DOI:10.1186/s13568-019-0936-9	Serotype distribution, antimicrobial susceptibility, antimicrobial resistance genes and virulence genes of Salmonella isolated from a pig slaughterhouse in Yangzhou, China	2019
7194	DOI:10.1111/jam.14515	Isolation and characterization of Salmonella in pork samples collected from retail and wholesale markets in each season from 2016 to 2018 in Wuhan, China	2020
7197	DOI:10.3389/fmicb.2019.02025	Epidemiological and Molecular Investigations on Salmonella Responsible for Gastrointestinal Infections in the Southwest of Shanghai From 1998 to 2017	2019
7201	DOI:10.1002/fsn3.1126	Antimicrobial resistance and genetic diversity of Salmonella enterica from eggs	2019
7205	DOI:10.1089/fpd.2019.2671	Prevalence and Characterization of Antimicrobial Resistance in Salmonella enterica Isolates from Retail Foods in Shanghai, China	2020
7207	DOI:10.4315/0362-028X.JFP-19-001	Prevalence and Characteristics of Quinolone Resistance in Salmonella Isolated from Retail Foods in Lanzhou, China	2019
7210	DOI:10.3389/fmicb.2019.01513	Antibiotic Resistance Profiles of Salmonella Recovered From Finishing Pigs and Slaughter Facilities in Henan, China	2019

Table S2

No.	Identificatin methods	Province	Sampling time	Host	Sample size	Number of isolates
18	culture, biochemical	Gansu	2013-2019	Human	2230	74
40	culture, PCR	Xinjiang	2017	Dogs and Cats	307	99
45	culture	Hunan	2019-2020	Swine	302	17
51	culture, biochemical	Shanghai	2015-2019	Human	1810	102
54	culture, biochemical	Zhejiang	2019	Human	5459	158
65	culture, biochemical	Shanghai	2018-2019	Human	1810	155
69	culture, biochemical	Guangdong	2016-2018	Human	2658	258
79	culture, biochemical	Guangxi	2017-2019	Human	122340	2845
98	culture, biochemical	Liaoning	2020	Poultry	390	49
110	culture, PCR	Henan	2018-2019	Swine	825	241
111	culture	Shanxi	2014-2019	Human	9209	517
113	culture	Beijing	2017-2019	Human	18182	874
114	culture, AIS, biochemical	Guangdong	2019-2020	Human	617	39
126	culture, mass spectrometry, AIS	Shaanxi	2018-2020	Human	1435	106
129	culture, PCR	Guangdong	2020	Human	26253	85
135	culture	Henan	2016-2020	Human	628	126
142	culture, biochemical	Shandong	2020-2021	Human	1300	164
145	culture, serum agglutination	Heilongjiang	2016-2019	Human	9977	303
150	culture, PCR	Zhejiang	2018-2020	Human	4515	193
165	culture, PCR	Jiangsu	2019	Human	1246	35
168	culture, biochemical	Beijing	2017-2019	Human	1057	66
169	culture, PCR	Shandong	2020	Poultry	115	11
		Henan	2020	Poultry	120	1
		Hebei	2020	Poultry	120	26
		Anhui	2020	Poultry	145	16
179	culture, AIS	Beijing	2013-2018	Human	2076	113
		Gansu	2020	Food	312	2
192	culture	Beijing	2015	Human	662	11
		Beijing	2016-2019	Human	1442	75
200	culture, AIS, PCR	Beijing	2014-2019	Human	1870	84
202	culture, PCR	Chongqing	2020	Dogs	686	26
		Chongqing	2020	Cats	326	9
		Chongqing	2020	animals	6	6
		Chongqing	2020	Parrot	19	0
		Chongqing	2020	Hedgehog	1	0
218	culture, biochemical	Zhejiang	2019	Human	529	56
233	culture, biochemical	Beijing	2016-2019	Human	1675	68
239	culture	Jiangsu	2018	Human	1246	43
246	culture, AIS, biochemical	Tianjin	2018-2020	Human	607	74
250	agglutination, AIS	Beijing	2015-2019	Human	1810	102
259	culture, AIS, biochemical	Yunnan	2017-2020	Human	29674	151
261	culture, AIS	Shanghai	2018-2019	Swine	612	1
264	culture, biochemical	Jiangsu	2019	Human	1036	40
271	culture, microscope examination, PCR	Hebei	2019-2020	Poultry	588	150
292	culture, biochemical	Jiangxi	2016-2018	Human	5339	170
294	culture, biochemical, AIS	Jilin	2011	Food	1554	7
		Jilin	2012	Food	1217	2
		Jilin	2013	Food	2925	12
		Jilin	2014	Food	1513	9
		Jilin	2015	Food	1982	18
		Jilin	2016	Food	1561	16
		Jilin	2017	Food	1338	4
		Jilin	2018	Food	982	7
303	culture, AIS, biochemical, 16srRNA	Xinjiang	2019	Food	330	11
304	culture, PCR	Jiangsu	2015-2017	Human	389	15
306	culture, PCR	Jiangsu	2018-2019	Poultry	360	110
309	culture, PCR	Zhejiang	2007-2018	Human	3327	288
315	culture, AIS	Shanghai	2018-2019	Swine	114	53
		Shanghai	2018-2019	Poultry	92	55
		Shanghai	2018-2019	Ruminants	102	14
317	culture, AIS	Guizhou	2019	Human	347	36
323	culture, biochemical	Shandong	2018-2019	Human	452	120
329	culture, biochemical	Shanghai	2015-2018	Human	2871	94
348	biochemical, PCR	Jilin	2019	Poultry	1024	148
355	culture, biochemical	Shanghai	2014-2019	Food	5667	300
356	culture, AIS	Jiangsu	2019	Human	324	85
373	culture, biochemical	Zhejiang	2015-2018	Human	1933	205
381	culture, biochemical	Liaoning	2019	Food	1786	54
		Liaoning	2019	Human	600	55
397	culture, biochemical	Hubei	2015-2017	Human	1216	71
400	culture, PCR	Sichuan	2012-2019	Human	1829	160
411	culture, biochemical	Tianjin	2017-2018	Human	1067	31

417	culture, PCR	Jiangsu	2016-2018	Poultry	329	49
423	culture, biochemical	Shanghai	2016-2018	Human	414	48
426	culture, biochemical, PCR	Tibet	2019	Swine	300	33
427	culture, AIS	Anhui	2010-2018	Human	2998	265
434	culture	Henan	2018-2019	Human	744	21
442	culture, biochemical	Jiangsu	2019	Human	3582	105
446	culture, AIS	Jiangsu	2014-2018	Human	1943	249
447	culture, biochemical, PCR	Guangdong	2016-2018	Poultry	435	17
451	culture	Beijing	2014-2017	Human	1520	95
453	culture, biochemical	Shanghai	2018	Human	5580	234
457	culture, biochemical	Xinjiang	2017-2019	Human	1200	641
462	culture, biochemical	Zhejiang	2017-2019	Human	1363	43
467	culture	Yunnan	2018-2019	Human	8558	320
470	NA	Henan	2019	Human	495	78
474	culture, PCR	Xinjiang	2016	Poultry	400	110
		Xinjiang	2016	Ruminants	97	63
		Xinjiang	2016	Swine	400	40
		Xinjiang	2016	Ruminants	250	17
477	culture, AIS	Jiangsu	2012-2019	Human	9096	563
478	culture, biochemical, PCR	Hubei	2018	Swine	640	39
503	culture, biochemical	Guangdong	2017-2019	Human	2312	343
509	culture, PCR	Anhui	2014-2018	Food	717	11
515	culture, biochemical	Guangxi	2018	Human	8702	286
561	culture, AIS	Shaanxi	2018	Human	2294	101
563	culture, AIS	Zhejiang	2015-2017	Human	932	208
571	culture, PCR	Shanghai	2013-2017	Human	9301	305
576	culture	Guangxi	2014-2017	Human	408	22
577	culture, AIS	Guangdong	2015-2017	Human	12890	532
579	culture	Guangdong	2016	Human	345	55
586	culture, biochemical	Beijing	2014-2018	Human	1827	89
600	culture	Xinjiang	2018-2019	Ruminants	841	44
654	culture, biochemical	Shanghai	2015-2018	Human	3845	139
657	culture, biochemical	Guangxi	2018	Human	401	29
666	culture	Guangdong	2017-2018	Human	1219	128
684	culture, biochemical	Zhejiang	2014-2018	Human	2327	229
704	culture, AIS	Hainan	2016-2018	Human	6837	766
705	culture, biochemical	Shaanxi	2016-2018	Human	1329	28
719	culture, AIS	Beijing	2017-2018	Human	380	19
726	culture, biochemical	Beijing	2018	Human	380	23
738	culture, AIS	Anhui	2013-2015	Human	2618	76
750	culture, biochemical	Guangdong	2015-2018	Human	2619	594
764	culture, mass spectrometry	Yunnan	2016-2018	Human	60533	186
766	culture, mass spectrometry	Zhejiang	2018	Poultry	596	78
772	culture, PCR	Hunan	2017-2018	Swine	300	37
		Hunan	2017-2018	Poultry	170	32
778	culture	Jiangsu	2015-2017	Poultry	80	11
		Jiangsu	2015-2017	Food	259	4
		Jiangsu	2015-2017	Human	1606	79
784	culture, AIS	Chongqing	1991-2017	Human	33957	319
794	culture, biochemical	Zhejiang	2013	Human	517	31
798	culture, biochemical	Sichuan	2012-2017	Human	5934	340
800	culture, biochemical, AIS	Guangdong	2016-2017	Human	905	120
816	culture, PCR	Henan	2018	Human	2083	12
833	culture, AIS	Guangdong	2012-2017	Poultry	129	25
			2012-2017	Food	501	27
834	culture, microscope examination, PCR	Jiangsu	2015-2018	Poultry	5889	56
854	culture, PCR	Chongqing	2018	Poultry	350	8
863	culture, biochemical, PCR	Henan	2018	Swine	840	45
874	culture, biochemical	Beijing	2016-2018	Human	1269	58
875	culture, biochemical	Jiangsu	2015-2018	Human	1000	100
882	microscope examination	Guangxi	2015-2017	Poultry	1554	55
884	culture, PCR	Liaoning	2017-2018	Swine	412	39
885	culture	Henan	2015-2018	Food	465	17
		Henan	2015-2018	Poultry	43	24
887	culture, AIS	Zhejiang	2017-2018	Food	2039	37
896	culture, AIS	Beijing	2013-2017	Human	1672	58
908	culture	Hebei	2015-2017	Food	680	24
909	culture, AIS	Beijing	2015-2018	Human	1847	37
923	NA	Beijing	2016-2017	Human	1123	26
924	culture, AIS	Guangxi	2018	Human	1238	63
931	culture, AIS	Guangdong	2017	Human	10487	422
946	culture, biochemical, AIS	Jiangsu	2016-2017	Human	982	32
961	culture, biochemical	Beijing	2017	Human	515	18
963	culture, PCR	Shanghai	2015-2018	Human	333	18

964	culture, biochemical	Guangdong	2014-2017	Human	2685	180
984	culture, biochemical	Ningxia	2010-2016	Human	1175	51
991	culture, biochemical	Shanghai	2018	Poultry	615	70
997	culture, biochemical	Shanxi	2015-2017	Human	752	17
1001	culture, biochemical	Shaanxi	2018	Human	594	58
1002	culture, biochemical	Henan	2017	Human	504	100
1009	culture, biochemical	Zhejiang	2016	Human	49774	1102
1013	culture, AIS	Zhejiang	2013-2017	Human	1664	78
1014	culture, AIS	Guangdong	2015-2017	Human	4019	108
1024	culture, biochemical	Shanxi	2015-2016	Human	2505	51
1030	culture, biochemical	Shanghai	2013-2017	Human	16860	222
1032	culture, biochemical	Guangdong	2013-2015	Human	3080	402
1040	culture, PCR	Beijing	2017	Human	352	10
1043	culture, AIS	Liaoning	2017	Poultry	632	131
1046	culture, AIS	Guangdong	2015-2017	Human	3757	271
1056	culture, AIS	Qinghai	2015-2016	Human	817	17
1062	culture, biochemical, PCR	Liaoning	2017	Poultry	2000	73
1074	culture, biochemical	Sichuan	2016-2017	Human	36451	43
1079	culture, biochemical	Henan	2011-2013	Poultry	1699	149
		Henan	2011-2013	Ruminants	690	24
		Henan	2011-2013	Swine	1382	138
		Henan	2011-2013	Food	532	20
1081	culture, PCR, AIS	Henan	2017	Poultry	351	62
1094	culture	InnerMongolia	2017	Ruminants	317	30
1095	culture, PCR	Shandong	2015	Swine	1480	298
1098	culture	InnerMongolia	2018	Ruminants	317	30
1100	culture, biochemical	Shandong	2016-2017	Human	2400	27
1103	culture, AIS	Sichuan	2015-2017	Food	376	32
1106	culture, AIS	Guangdong	2013-2016	Human	5820	480
1127	culture, AIS	Beijing	2015-2016	Human	732	31
1140	NA	Zhejiang	2015-2017	Human	1258	25
1148	culture, AIS, PCR	Hebei	2017	Food	570	7
1150	culture, biochemical	Henan	2017	Poultry	452	97
1159	culture, AIS	Zhejiang	2014-2016	Human	883	17
1175	culture, biochemical	Jiangsu	2015-2016	Swine	116	12
		Jiangsu	2015-2016	Poultry	189	24
		Jiangsu	2015-2016	Ruminants	63	1
1180	culture, biochemical, PCR	Sichuan	2018	Poultry	343	99
1186	culture, biochemical	Hebei	2016	Human	36452	6
1189	culture, AIS	Shandong	2009-2016	Swine	85	19
		Shandong	2009-2016	Ruminants	30	7
		Shandong	2009-2016	Poultry	227	83
1190	culture, biochemical	Guangdong	2014-2016	Human	90887	24
1194	culture, biochemical	Guizhou	2013-2014	Human	432	35
1213	culture, AIS	Beijing	2014-2017	Human	1453	76
1225	culture, AIS	Shanghai	2016-2017	Human	400	22
1235	culture, biochemical	Henan	2015	Poultry	554	77
1237	culture, biochemical	Shanghai	2016	Swine	254	82
		Shanghai	2016	Poultry	306	75
		Shanghai	2016	Ruminants	46	1
1248	culture, biochemical	Jiangsu	2016	Human	417	5
1262	culture, AIS	Jiangsu	2010-2016	Food	719	53
1271	culture, AIS, biochemical	Beijing	2016	Human	340	21
1281	culture, AIS	Beijing	2013-2017	Human	1455	73
1287	culture, biochemical	Shandong	2017	Human	665	21
1288	culture	Guangxi	2016	Food	12120	70
		Guangxi	2016	Human	5400	234
1294	culture	Xinjiang	2013-2016	Human	1552	29
1295	culture, biochemical	Yunnan	2015	Human	10342	56
1305	culture, AIS	Sichuan	2017	Swine	600	49
1308	culture, biochemical	Guangdong	2014-2017	Human	4847	192
1310	culture, biochemical	Guangdong	2016	Human	7673	15
1311	culture, AISbiochemical	Beijing	2012-2017	Human	16349	677
1314	culture	Shanghai	2011-2014	Poultry	264	77
		Shanghai	2011-2014	Swine	144	40
		Shanghai	2011-2014	Ruminants	192	37
1324	culture, biochemical	Jiangxi	2015-2016	Human	1260	30
1325	culture, PCR	Xinjiang	2017	Ruminants	753	63
1333	culture, AIS	Guangdong	2015-2017	Human	1136	108
1339	culture, PCR	Henan	2014-2015	Poultry	695	263
1345	culture, AISbiochemical	Guangdong	2015	Human	4620	61
1354	culture, biochemical	Guangdong	2016	Poultry	316	243
1359	culture, PCR	Jiangsu	2017	Swine	1727	184
1362	culture, AIS	Beijing	2015-2017	Human	1335	126

1371	culture, AIS	Shandong	2011-2017	Poultry	800	104
		Shandong	2011-2017	Food	881	18
1387	culture, PCR	Xinjiang	2015	Poultry	950	80
1389	culture, AIS	Beijing	2010-2015	Human	3428	156
1405	culture, PCR	Zhejiang	2014-2015	Human	417	16
1407	culture, AISbiochemical	Shandong	2010-2015	Human	513	31
1413	culture, PCR	Henan	2016	Swine	840	67
1419	culture, biochemical, PCR	Guangdong	2014-2015	Human	412	50
1428	culture, AISbiochemical	Beijing	2015-2016	Human	3068	33
1440	culture, biochemical	Sichuan	2017	Swine	15613	6
1456	culture	Chongqing	2013-2015	Human	2538	155
1458	culture	Anhui	2012-2014	Dogs	746	17
		Anhui	2012-2014	Feed	404	10
1474	culture	Henan	2015-2016	Human	5720	221
1482	NA	Hunan	2016	Food	3647	121
1507	culture, biochemical	Jiangsu	2014-2016	Poultry	427	36
		Jiangsu	2014-2016	Food	3759	76
1533	AIS	Shaanxi	2008-2014	Human	2113	34
1554	culture, PCR	Beijing	2013-2015	Human	947	52
1567	culture, biochemical	Jiangxi	2015-2016	Human	600	19
1579	culture, biochemical	Heilongjiang	2016	Poultry	880	86
1587	spectrometry	Shanghai	2016	Poultry	500	28
1598	NA	Zhejiang	2014	Human	317	44
1620	culture, biochemical	Sichuan	2012-2015	Human	86971	43
1628	NA	Guangdong	2015-2016	Human	1643	49
1634	NA	Zhejiang	2014-2016	Human	6262	54
1639	culture, biochemical	Jiangsu	2016	Human	71694	118
1648	culture, AIS	Shanghai	2010-2014	Human	2119	374
1664	culture, biochemical, PCR	Jilin	2016-2019	Swine	450	17
1671	NA	Beijing	2015	Human	403	25
1691	culture, AIS	Fujian	2000-2013	Poultry	663	79
		Fujian	2000-2013	Swine	614	87
		Fujian	2000-2013	Ruminants	800	90
		Fujian	2000-2013	Food	7635	52
1696	culture, AIS, PCR	Beijing	2012-2016	Human	1948	122
1704	culture, AIS	Guangdong	2014-2015	Human	555	61
1706	culture, biochemical	Zhejiang	2015-2016	Human	802	17
1709	culture, biochemical	Zhejiang	2014	Human	4095	46
1734	culture, PCR	Xinjiang	2015	Poultry	950	80
1754	culture, PCR	Zhejiang	2014-2015	Human	417	16
1761	culture, PCR	Henan	2016	Swine	840	67
1765	culture, PCR	Jiangsu	2015	Human	756	32
1767	culture, PCR	Guangdong	2014-2015	Human	412	50
1777	culture, AIS	Henan	2016	Poultry	2915	1031
1791	culture, PCR	Sichuan	2016	Human	15613	6
1803	culture, AIS	Beijing	2014-2016	Human	1400	56
1826	culture, biochemical	Henan	2015	Human	2824	79
		Henan	2016	Human	2896	142
		Chongqing	2015	Poultry	94	11
		Chongqing	2015	Swine	168	35
1849	culture, biochemical	Sichuan	2015	Human	230	27
		Sichuan	2016	Human	264	32
		Chongqing	2015	Poultry	94	11
		Chongqing	2015	Swine	168	35
1920	culture, AIS, biochemical	Liaoning	2013-2015	Human	1288	439
1938	culture, biochemical	Jiangsu	2013-2014	Human	609	14
1946	NA	Xinjiang	2013-2015	Human	1363	15
1956	culture, biochemical	Shanghai	2015	Food	1057	8
		Shanghai	2015	Poultry	144	3
1986	culture, biochemical	Guangdong	2014-2015	Human	2456	283
1987	culture, biochemical	Zhejiang	2014	Human	3927	46
1997	culture, PCR	Guangdong	2014-2015	Human	1319	76
2000	culture, biochemical	Jiangsu	2015	Poultry	88	18
				Ruminants	80	9
				Swine	65	4
				animals	152	1
2002	culture, biochemical	Shanghai	2010-2014	Human	8353	285
2005	culture, biochemical	Henan	2010-2014	Human	1762	82
2013	culture, PCR	Guangdong	2013-2014	Human	1719	76
2014	culture, AIS	Zhejiang	2011-2014	Human	14658	447
2021	culture, biochemical	Guangdong	2013-2015	Human	3373	271
2029	culture, PCR	Jiangsu	2014	Human	915	55
2061	culture, biochemical	Heilongjiang	2012	Poultry	1590	339

2065	culture, biochemical, AIS	Jiangsu	2014-2015	Human	860	108
2078	culture, biochemical	Hunan	2013-2015	Food	545	14
2086	culture, PCR	Shanghai	2014	Human	380	10
2091	culture, AIS	Jiangsu	2012-2014	Human	2678	87
2097	culture, biochemical	Beijing	2014	Human	308	19
2115	culture, AIS	Zhejiang	2014	Human	499	5
2133	culture	Shanghai	2011-2013	Human	697	147
2134	NA	Heilongjiang	2010-2015	Human	1754	26
2138	culture, biochemical, AIS	Guangdong	2011-2013	Human	2893	227
2140	culture, biochemical	Shanghai	2014	Human	1218	47
2149	culture, biochemical	Zhejiang	2013-2014	Human	426	26
2161	culture, microscope examination, serur	Sichuan	2014	Swine	600	27
2165	culture, AIS	Guangdong	2010-2014	Human	6707	290
2173	culture, biochemical	Shanghai	2011-2014	Human	7626	360
2183	culture	Guangxi	2016	Swine	300	98
2190	culture	Gansu	2013-2015	Human	1617	9
2191	culture, AIS	Sichuan	2012-2014	Human	982	35
2201	culture, biochemical, AIS	Fujian	2009-2014	Human	3287	156
2203	culture	InnerMongolia	2014	Human	771	43
2224	culture	Shanghai	2015	Food	780	11
2254	culture, AIS	Guangdong	2014-2015	Human	2465	283
2265	culture	Guangdong	2014－2015	Human	1319	76
2268	culture	Jiangsu	2015	Food	385	32
2270	culture	Shanghai	2010-2014	Human	8535	285
2281	culture	Guangdong	2013-2014	Human	1719	76
2282	culture, AIS, biochemical	Zhejiang	2011-2014	Human	14658	447
2289	culture	Guangdong	2013-2015	Human	3373	271
2329	culture	Heilongjiang	2012	Poultry	3766	339
2333	culture, AIS	Zhejiang	2014-2015	Human	860	108
2346	culture	Hunan	2013-2015	Food	545	14
2354	culture	Shanghai	2014	Human	380	10
2359	culture, AIS	Jiangsu	2012-2014	Human	2678	87
2365	culture, biochemical	Beijing	2014	Human	308	19
2402	culture, AIS	Heilongjiang	2010-2015	Human	1754	26
2406	culture, AIS	Guangdong	2011-2013	Human	2893	227
2408	culture, microscope examination, PCR	Shanghai	2014	Human	1218	47
2417	culture	Zhejiang	2013-2014	Human	426	26
2423	culture	Guangdong	2012-2015	Human	7047	459
2429	culture, PCR, microscope examination	Sichuan	2014-2015	Swine	600	27
2441	culture, biochemical	Shanghai	2011-2014	Human	7626	360
2445	culture, biochemical	Fujian	1993-2015	Human	15074	474
2448	culture, AIS	Guangdong	2014	Human	3745	79
2454	culture	Jiangsu	2015	Human	464	25
2469	culture, biochemical	Fujian	2009-2014	Human	3287	156
2490	culture	Zhejiang	2013-2014	Human	1698	5
2496	culture	Xinjiang	2013-2014	Ruminants	535	30
2499	culture	Shandong	2014	Swine	1000	188
2500	culture	Guangdong	2010-2013	Human	3604	142
2504	culture	Shandong	2012-2013	Human	13	1
		Shandong	2012-2013	Poultry	720	128
2508	culture, AIS	Shanghai	2011-2012	Human	544	20
2526	culture	Guangdong	2013-2014	Human	582	24
2531	culture, PCR	Zhejiang	2011-2013	Cold-blooded animals	800	47
2544	culture, biochemical	Shanghai	2012	Human	2423	69
2560	culture, biochemical	Zhejiang	2010-2013	Human	10917	614
2575	culture	Guangdong	2010-2013	Human	1922	92
2578	culture, biochemical	Beijing	2013-2014	Human	605	35
2595	culture, PCR	Beijing	2014	Human	330	20
2596	culture	Beijing	2013-2014	Human	542	34
2603	culture, biochemical, PCR, AIS	Sichuan	2009-2014	Poultry	5445	154
2605	culture, biochemical	Zhejiang	2014	Food	6812	208
2623	culture	Zhejiang	2014	Swine	300	104
2627	culture, PCR	Guangxi	2012-2014	Ruminants	192	26
		Guangxi	2012-2014	Swine	192	66
2632	culture, AIS	Guangdong	2013	Human	9847	329
2652	culture	Shandong	2012	Poultry	1267	298
2653	culture	Beijing	2011-2013	Human	1179	69
2665	culture, serum agglutination	Guangxi	2013-2015	Poultry	310	34
2684	culture, PCR	Shandong	2014	Poultry	995	239
2689	culture	Henan	2012	Poultry	802	234
		Henan	2013	Poultry	654	169
2706	culture, AIS, PCR	Beijing	2013-2014	Human	366	18
2723	culture	Guangdong	2012-2013	Human	863	80
2725	culture	Guangdong	2007-2013	Human	63687	386

2738	culture, biochemical, PCR	Sichuan	2009-2014	Swine	2660	151
2749	culture, biochemical	Shanghai	2010-2012	Human	2579	185
2753	culture, biochemical, PCR	Shanxi	2013-2014	Human	789	18
2756	culture, biochemical	Zhejiang	2008-2015	Human	9256	369
2761	culture	Jilin	2013	Swine	540	18
2768	culture	Xinjiang	2014	Swine	550	58
2773	culture, AIS	Guangdong	2009-2014	Human	4395	546
2788	culture	Guangdong	2012-2013	Poultry	312	23
		Guangdong	2012-2013	Food	264	82
2795	culture, biochemical, AIS	Hunan	2010	Poultry	152	36
		Hunan	2010	Food	540	58
2803	culture	Sichuan	2007-2012	Food	1081	19
2815	culture, biochemical	Hunan	2009-2011	Human	767	35
2817	culture, biochemical	Zhejiang	2011-2013	Human	361	7
2822	culture	InnerMongolia	2006-2011	Ruminants	460	38
2834	culture	Jiangxi	2009-2014	Food	3450	136
2837	culture, PCR	Shandong	2006-2007	Poultry	992	178
2851	culture	Sichuan	2012	Poultry	588	57
				Poultry	127	16
2863	culture	Guangdong	2013	Human	1150	72
2889	culture, biochemical	Heilongjiang	2012	Swine	600	60
2893	culture, biochemical, PCR	Heilongjiang	2012	Swine	600	35
2900	culture	Guangxi	2009-2012	Feed	468	7
2906	culture, biochemical	Shanghai	2012	Poultry	320	70
2912	culture, biochemical	Shanghai	2013	Human	583	19
2924	culture	Guangdong	2010-2012	Human	1095	45
2946	culture, biochemical, AIS	Shandong	2012	Poultry	2496	497
2964	culture	Guangdong	2010-2013	Human	6417	460
2965	culture, biochemical	Guangdong	2007-2009	Human	760	53
2993	culture, AIS	Hebei	2013	Poultry	331	89
2994	culture, biochemical, AIS	Guangdong	2009-2013	Human	6920	344
3001	culture, AIS	Zhejiang	2004-2013	Human	1258	32
3006	culture	Fujian	2010-2012	Food	477	12
3022	culture	Guangxi	2009-2012	Food	701	31
3023	culture	Sichuan	2007-2009	Human	1008	52
3033	culture, PCR	Henan	2013	Swine	1820	125
3037	culture, AIS	Henan	2012	Food	344	6
3317	culture	Hunan	2012	Food	991	12
3462	culture	Hubei	2009	Human	1022	15
3488	culture, AIS	Guangdong	2010	Human	1027	20
3534	culture	Shanghai	2010-2011	Human	11035	64
3538	culture	Jiangsu	2010	Poultry	414	94
3542	culture	Ningxia	2009-2011	Food	441	11
3567	culture	Henan	2011	Mouse	386	137
3593	culture	Jiangsu	2010-2011	Human	456	8
3604	culture	Sichuan	2010	Human	433	25
3618	culture	Jiangsu	2009-2011	Poultry	212	58
		Jiangsu	2009-2011	Food	3225	97
3633	culture	Hebei	2010	Human	117288	93
3649	culture	Shanghai	2006-2010	Human	4105	181
3651	culture	Jiangsu	2011	Food	891	67
3664	culture	Shanghai	2009-2010	Human	4170	206
		Shanghai	2009-2010	Food	632	69
3673	culture	Yunnan	2009	Human	588	2
3675	culture	Henan	2009-2010	Human	1509	123
3679	culture, AIS	Hunan	2009-2010	Human	10072	82
3688	culture, biochemical	Shanghai	2010	Poultry	189	21
		Shanghai	2010	Food	651	15
3716	culture	Sichuan	2006-2008	Food	2559	76
3727	culture, PCR	Guangdong	2008-2009	Human	2472	44
3732	culture, biochemical	Jiangsu	2006-2010	Food	533	33
3734	culture, AIS	Gansu	2007-2010	Human	739	9
3744	culture, biochemical	Shanghai	2010	Human	1045	160
3763	culture, biochemical	Henan	2006-2010	Human	2327	85
3773	culture, AIS	Sichuan	2007-2010	Human	1130	14
3774	culture, biochemical	Beijing	2010	Human	308	10
3778	culture, AIS	Guangdong	2008-2009	Human	65613	162
3787	culture	Guangdong	2009-2010	Human	1665	72
3827	culture	Hunan	2010	Human	11068	118
3837	culture, biochemical	Hubei	2006	Human	3746	221
3842	culture, PCR	Anhui	2010	Poultry	500	21
3856	culture, biochemical	Shaanxi	2007-2008	Poultry	515	260
3867	culture, biochemical	Jiangsu	2008-2010	Human	58382	258
3870	culture	Guangdong	2008-2011	Food	2321	164

3878	culture, AIS	Guangdong	2008-2010	Human	1260	52
3887	culture, AIS	Xinjiang	2010	Human	392	15
3913	culture, biochemical	Anhui	2008	Human	480	13
3918	culture, AIS, serum agglutination	Guangxi	2006-2009	Human	1930	32
3919	culture	Jiangxi	2008-2009	Human	1630	9
3935	culture	Shanghai	2004-2009	Human	189068	134
3937	culture	Heilongjiang	2005-2008	Food	1070	94
3938	culture, biochemical, PCR	Anhui	2008-2009	Swine	360	22
3942	agglutination	Zhejiang	2008-2009	Swine	157	16
		Zhejiang	2008-2009	Poultry	135	12
		Zhejiang	2008-2009	Food	19	2
		Zhejiang	2008-2009	Ruminants	46	1
3956	culture	Shandong	2003	Swine	41	1
		Shandong	2003	Poultry	100	7
		Shandong	2003	Ruminants	127	1
		Shandong	2003	Food	122	0
		Shandong	2004	Swine	45	1
		Shandong	2004	Poultry	100	8
		Shandong	2004	Ruminants	80	1
		Shandong	2003	Food	78	0
		Shandong	2005	Swine	40	3
		Shandong	2005	Poultry	101	4
		Shandong	2005	Ruminants	82	2
		Shandong	2005	Food	162	0
		Shandong	2006	Swine	14	1
		Shandong	2006	Poultry	74	3
		Shandong	2006	Ruminants	28	0
		Shandong	2006	Food	168	1
		Shandong	2007	Swine	29	3
		Shandong	2007	Poultry	23	2
		Shandong	2006	Ruminants	14	0
		Shandong	2007	Food	78	0
		Shandong	2008	Swine	23	3
		Shandong	2008	Poultry	50	5
		Shandong	2008	Ruminants	29	3
		Shandong	2008	Food	77	0
3964	culture	Guangxi	2009	Poultry	120	31
		Guangxi	2009	Food	1061	30
3972	culture, PCR	Henan	2009-2010	Poultry	617	98
3975	culture	Shanghai	2009	Human	2936	32
3982	culture, AIS	Sichuan	2009	Food	827	51
3996	culture, AIS	Jiangsu	2008	Poultry	33	11
		Jiangsu	2008	Food	724	33
4010	culture, biochemical	Henan	2008-2009	Poultry	362	96
4011	culture	Gansu	2007-2009	Human	683	7
4023	culture, AIS	Guangdong	2006-2008	Food	369	18
4035	culture, biochemical	Shanxi	2008-2009	Poultry	961	177
4038	culture	Shanxi	2002-2008	Swine	290	56
		Shanxi	2002-2008	Ruminants	245	53
		Shanxi	2002-2008	Ruminants	640	95
4044	culture	Shanxi	2007-2008	Food	764	359
4047	culture, biochemical	Zhejiang	2007-2009	Human	719	18
4057	culture	Jiangxi	2008	Mouse	358	134
4067	culture, biochemical	Jiangsu	2005-2007	Human	47132	67
4088	culture, AIS	Guangxi	2007-2008	Human	1400	43
4091	culture, biochemical	Shanghai	2008	Human	83032	175
4094	culture, AIS	Jiangsu	2008	Poultry	93	16
		Jiangsu	2008	Food	1738	54
4105	culture, biochemical	Beijing	2008	Human	301	32
4121	culture, AIS	Jiangsu	2010	Food	372	19
4126	culture, AIS	Jiangxi	2007-2009	Human	1126	16
4147	culture	Beijing	2008	Human	786	38
4159	culture	Guangxi	2007	Human	19307	113
4163	culture, AIS	Fujian	2007	Human	2871	10
4169	culture	Zhejiang	2001-2008	Human	47228	189
4191	culture, AIS, microscope examination	Zhejiang	2005-2007	Human	1143	47
4199	culture, biochemical, AIS	Shandong	2007-2009	Human	1100	20
4206	culture, biochemical, AIS	Henan	2006-2007	Poultry	225	95
		Henan	2006-2007	Swine	154	26
4210	culture, biochemical	Zhejiang	2004-2007	Human	73601	217
4230	NA	Hunan	2007-2008	Food	130	47
4232	culture, biochemical	Chongqing	2007	Human	835	79
4239	culture, biochemical	Zhejiang	2003-2007	Human	2056	251
4245	culture, biochemical	Guangdong	2005-2008	Swine	125	13

		Guangdong	2005-2008	Ruminants	59	7
		Guangdong	2005-2008	Poultry	122	12
		Guangdong	2005-2008	Food	329	17
4246	culture	Guangdong	2004-2009	Swine	85	5
		Guangdong	2004-2009	Ruminants	61	3
		Guangdong	2004-2009	Ruminants	50	3
		Guangdong	2004-2009	Poultry	84	9
		Guangdong	2004-2009	Food	628	10
4254	culture, biochemical	Beijing	2007	Human	313	34
4261	culture, AIS	Jiangsu	2007	Food	804	33
4262	culture	Shanxi	2002-2008	Food	522	33
4272	culture	Guangxi	2007-2008	Human	35847	165
4312	culture	Beijing	1998-2007	Human	859	89
4315	culture	Shanghai	2004-2008	Human	154097	537
4318	culture	Shandong	2003-2005	Swine	169	9
		Shandong	2003-2005	Poultry	401	24
		Shandong	2003-2005	Ruminants	334	4
		Shandong	2003-2005	Food	929	1
4347	culture	Henan	2008	Swine	136	18
		Henan	2008	Ruminants	106	10
		Henan	2008	Poultry	82	7
		Henan	2008	Food	570	11
4352	culture	Shanghai	2006-2008	Human	8374	103
4361	culture	Beijing	2007	Human	1600	76
4374	culture, biochemical	Guangdong	2007	Human	1128	71
4378	culture	Guangdong	2001-2005	Human	151490	721
4380	culture	Hebei	2005-2007	Food	2058	143
4387	culture, biochemical	Sichuan	2007	Human	338	18
4391	culture	Zhejiang	2004-2007	Human	26318	69
4402	culture, AIS	Shanghai	2006	Human	4856	29
4409	culture	Shanghai	2004-2006	Human	79319	312
4415	culture	Guangdong	2004-2007	Human	3257	60
4437	culture	Henan	2006-2007	Poultry	1128	68
4447	culture, biochemical	Guangxi	2005	Food	682	73
4449	culture	Guangxi	2006-2007	Food	464	11
4466	culture	Shanxi	2002-2006	Food	2272	208
4469	culture	Hebei	2005-2007	Food	2058	144
4492	culture	Fujian	2006	Human	51548	86
4535	culture	Shanghai	2006	Human	3603	196
4641	culture, biochemical	Hubei	2004	Human	4536	64
4655	culture	Liaoning	2003-2005	Food	1965	6
4666	culture, biochemical	Guangdong	2004	Food	205	5
		Guangdong	2004	Poultry	60	6
		Guangdong	2004	Ruminants	105	10
		Guangdong	2004	Swine	60	7
		Guangdong	2004	Food	325	8
4676	culture	Shanghai	2006	Human	4856	29
4688	culture	Zhejiang	2004-2005	Human	384	2
4690	culture	Shandong	2004-2006	Human	126307	1051
4694	culture	Shandong	2005-2006	Human	636	17
4707	culture	Liaoning	2006	Swine	333	210
4740	culture, biochemical, AIS	Shanghai	2007	Human	165142	186
4754	culture	Jiangsu	2002-2006	Food	484	50
4771	culture	Hebei	2005	Food	386	81
4787	culture, biochemical, AIS	Zhejiang	2001-2004	Food	1047	98
4797	culture	Guangxi	2002-2005	Food	1872	128
4833	culture	Hebei	2005	Swine	45	12
		Hebei	2005	Poultry	120	19
		Hebei	2005	Ruminants	90	30
		Hebei	2005	Food	132	20
4848	magnetic beads, AIS	Shanghai	2002-2003	Food	303	112
4860	culture	Guangdong	2000	Food	4671	27
		Guangdong	2000-2004	Feed	60	3
4871	culture	Guangdong	1999-2004	Human	89286	233
4875	culture, biochemical	Ningxia	2001-2006	Human	1942	30
4907	culture, AIS	Henan	2006	Food	540	57
4914	culture, biochemical	Jiangsu	2002-2004	Poultry	58	0
		Jiangsu	2002-2004	Food	899	20
4926	culture	Guangdong	1998-2003	Human	51184	239
4936	culture	Guangdong	2002－2005	Human	452	8
5125	culture	Hubei	2003-2004	Human	693	9
5151	culture	Hebei	1998-2002	Human	36000	648
5157	culture	Shanghai	2001-2003	Human	283685	282
5158	culture	Fujian	2000	Food	332	12

5184	culture, biochemical	Jiangsu	2002-2003	Poultry	40	0
		Jiangsu	2002-2003	Food	410	10
5368	agglutination	Sichuan	2002	Human	72111	45
5370	culture, biochemical	Zhejiang	2001	Human	12850	25
5375	culture	Jiangxi	2001	Human	15420	193
5376	culture, biochemical	Fujian	2000-2002	Food	978	64
5384	culture, biochemical	Guangdong	2000-2001	Human	50630	130
5394		Shaanxi	1997-2002	Human	685	8
5396	culture, biochemical, AIS	Shaanxi	2002	Swine	41	11
		Shaanxi	2002	Poultry	40	4
		Shaanxi	2002	Ruminants	80	22
		Shaanxi	2002	Food	307	7
5397	culture, biochemical	Beijing	2002	Food	927	33
5400	culture, biochemical	Jiangsu	1999-2001	Human	90728	262
5413	culture, biochemical	Guangdong	2000-2001	Human	50630	130
5414	culture, biochemical	Hubei	1997-2000	Human	45353	558
5474	culture, biochemical	Jiangsu	2001	Food	341	14
5510	culture, biochemical	Shaanxi	1998-2000	Human	2489	36
5518	culture, biochemical	Guangdong	1999-2000	Human	10083	27
5520	culture, serum agglutination	InnerMongolia	2000	Poultry	12824	1688
5623	culture, biochemical	Guangdong	1997-1999	Human	126228	1051
5625	culture, serum agglutination	Sichuan	1999	Rabbit	38	3
		Sichuan	1999	Poultry	132	2
		Sichuan	1999	Food	116	16
		Sichuan	1999	Swine	212	92
		Sichuan	1999	Food	99	0
5627	culture, biochemical	Guangdong	1997-1998	Human	67885	152
5664	culture, biochemical	Fujian	1991-1994	Human	887	73
5665	culture, biochemical	Guangdong	1989-1993	Human	249496	748
5684	culture, biochemical	Fujian	1989-1999	Human	21053	222
5705	culture, biochemical	Anhui	1997-1998	Human	13472	301
5706	culture, biochemical	Henan	1998	Swine	1820	113
5710	culture, biochemical	Sichuan	1997	Human	30364	135
5743	culture, biochemical	Fujian	1994-1995	Human	21053	222
5759	culture, serum agglutination	Shanghai	1992-1996	Human	99729	274
5827	culture, biochemical	Beijing	1991-1995	Human	8126	167
5849	culture, biochemical	Guangdong	1991-1993	Human	50808	504
5866	culture	Shanxi	1989-1993	Swine	1159	305
				Poultry	300	65
5877	culture	Zhejiang	1983-1992	Human	30684	179
5878	culture, biochemical, PCR	Tibet	1993	Human	626	11
5892	culture, biochemical	Shandong	1993	Human	402	5
5916	culture, biochemical	Jiangxi	1989-1990	animals	590	367
5937	culture, biochemical	Zhejiang	1992	Swine	159	12
			1992	Poultry	325	73
5944	culture, biochemical	Guangdong	1990-1991	Human	47580	382
5956	agglutination	Qinghai	1992	Food	339	55
6174	culture, biochemical	Qinghai	1997	Human	1192	8
6203	culture, biochemical	Anhui	1995-1996	Human	660	20
6211	culture, biochemical	Jiangsu	1995	Human	6507	94
6225	culture, biochemical	Henan	1990	Human	372	105
6247	culture, biochemical	Henan	1992	Human	703	105
6252	culture, biochemical	Hunan	1988-1990	Human	1638	76
6254	culture, biochemical	Gansu	1989-1992	Poultry	766	70
6260	culture, biochemical	Guangdong	1990-1991	Human	16611	57
6264	culture, biochemical	Zhejiang	1978-1990	Human	22914	176
6279	NA	Zhejiang	1988-1989	Human	2659	8
6308	culture, biochemical	Shandong	1986-1989	Human	511	85
6326	culture, biochemical	Fujian	1988	Swine	453	152
		Fujian	1988	Ruminants	33	10
		Fujian	1988	Poultry	61	6
6336	culture, biochemical	Henan	1987-1988	Human	1253	40
6393	culture, biochemical	Hunan	1984	Food	5200	741
6346	culture, biochemical	Anhui	1985-1987	Human	29549	256
6430	NA	Hunan	1984	Human	435	9
6598	culture	Taiwan	2005-2006	Poultry	3160	359
6609	culture	Taiwan	2005-2007	Dogs	1109	168
6669	culture, biochemical	Zhejiang	2017-2020	Human	1295	103
6673	culture, biochemical	Hunan	2015-2019	Human	7506	732
6685	culture, PCR	Hebei	2019-2020	Poultry	588	150
6712	culture, biochemical	Taiwan	2014	Poultry	622	156
6733	culture, PCR	Taiwan	2014-2016	Poultry	2040	243
6788	culture	Taiwan	2008	Poultry	1121	110
6791	culture, biochemical	Taiwan	2005	Swine	440	58

6806	culture	Taiwan	2002-2003	Poultry	1595	164
6808	culture, biochemical	Taiwan	2005-2006	Cold-blooded animals	476	147
6837	culture, biochemical	Taiwan	2003-2005	Dogs	928	40
6877	culture	Taiwan	2000-2001	Poultry	2000	91
6990	culture	Guangdong	2012-2013	Poultry	113	29
		Guangdong	2012-2013	Swine	204	53
7039	culture	Hong Kong	1976	Swine	1026	344
7043	culture	Hong Kong	1953-1966	Swine	105290	477
7063	culture, serum agglutination	Henan	2014-2015	Poultry	2139	45
7080	culture, serum agglutination, PCR	Shanghai	2018-2019	Swine	1389	239
7085	culture, WGS	Sichuan	2019	Poultry	420	74
7091	culture, PCR,	Hubei	2019	Swine	896	155
7093	culture, serum agglutination, WGS	Beijing	2017	Food	1234	341
7094	culture, serum agglutination	Jiangsu	2018-2019	Dogs and Cats	469	27
7098	culture, serum agglutination, PCR	Shandong	2019-2020	Poultry	360	155
7101	culture, serum agglutination,	Shandong	2015-2017	Poultry	1288	86
7110	culture, serum agglutination	Shandong	2013	Poultry	62	28
		Shandong	2014	Poultry	124	55
		Shandong	2015	Poultry	113	35
		Shandong	2016	Poultry	109	35
		Shandong	2017	Poultry	120	34
		Shandong	2018	Poultry	395	93
7113	culture, PCR	Chongqing	2018-2019	Swine	724	92
7125	mass spectrometry, PCR	Guangdong	2013	Food	317	82
		Guangdong	2014	Food	440	157
		Guangdong	2015	Food	754	287
		Guangdong	2016	Food	1107	445
		Guangdong	2017	Food	371	145
7127	culture, AIS	Shandong	2018	Poultry	600	67
7145	culture，PCR	Shaanxi	2013-2014	Poultry	814	61
7173	culture, AIS	Guangdong	2017-2019	Poultry	365	151
7177	culture	Hubei	2016	Swine	1440	177
7185	culture	Jiangsu	2016-2017	Swine	459	80
7194	culture	Hubei	2016-2018	Swine	4744	922
7197	culture，AIS	Shanghai	1998-2017	Human	32544	565
7201	culture	Guangdong	2017-2018	Poultry	1000	54
7205	culture, AIS, PCR	Shanghai	2016-2017	Swine	143	46
		Shanghai	2016-2017	Poultry	127	34
		Shanghai	2016-2017	Ruminants	91	21
		Shanghai	2016-2017	Food	674	46
7207	culture	Gansu	2015	Swine	93	2
		Gansu	2015	Ruminants	88	3
		Gansu	2015	Poultry	58	4
		Gansu	2015	Food	246	2
		Gansu	2016-2018	Swine	132	5
		Gansu	2016-2018	Ruminants	362	10
		Gansu	2016-2018	Poultry	250	10
		Gansu	2016-2018	Food	953	7
7210	culture	Henan	2017	Swine	1732	337

Table S3

No.	Serotyping methods	Province	Sampling time	Host	Sample size	isolates	Serotype	isolates
18	Serum agglutination	Gansu	2013-2019	Human	2230	74	Bovismorbificans	1
							Chailey	1
							Derby	1
							Enteritidis	15
							Montevideo	2
							Newlands	1
							Newport	1
							Oranienburg	1
							Others	37
							Rissen	3
							Stanley	1
							Tennessee	1
							Typhimurium	9
45	Serum agglutination	Hunan	2019-2020	Swine	302	17	Derby	9
							Typhimurium	4
							Kentucky	2
							London	2
51	Serum agglutination	Beijing	2015-2019	Human	1810	102	Agona	5
							Albany	1
							Cubana	1
							Derby	1
							Litchfield	1
							London	1
							Muenchen	1
							Oranienburg	1
							Schoeneberg	1
							Typhi	1
							Infantis	2
							Kentucky	2
							Tshiongwe	2
							Senftenberg	3
							Braenderup	8
							Typhimurium	14
							Enteritidis	57
54	NA	Zhejiang	2019	Human	5459	158	Enteritidis	56
							Typhimurium	51
							Others	51
65	Serum agglutination	Shanghai	2018-2019	Human	1810	155	Enteritidis	45
							Typhimurium	33
							London	11
							Rissen	9
							Thompson	8
							Infantis	8
							Senftenberg	8
							Litchfield	6
							Bovismorbificans	4
							Braenderup	2
							Goldcoast	2
							Cubana	2
							Livingstone	1
							Albany	1
							Wandsworth	1
							Weltevreden	4
							Derby	1
							Agona	1
							Indiana	1
							Stanley	1
							Meleagridis	1
							Give	1
							Anatum	1
							Rubislaw	1
							Others	1
							Amoutive	1
69	Serum agglutination	Guangdong	2016-2018	Human	2658	258	Typhi	4
							Typhimurium	8
							Others	246
79	Serum agglutination	Guangxi	2017-2019	Human	122340	2845	Typhimurium	300
							Corvallis	277
							Rissen	266
							Agona	227
							Derby	159
							Others	1616

98	Serum agglutination	Liaoning	2020	Poultry	390	49	Pullorum	26
							Typhimurium	17
							Paratyphi A	4
							Manhattan	2
113	Serum agglutination	Beijing	2017-2019	Human	18182	874	Enteritidis	406
							Typhimurium	160
							Thompson	34
							Senftenberg	28
							Agona	25
							London	23
							Goldcoast	19
							Stanley	14
							Braenderup	13
							Mbandaka	12
							Others	140
114	Serum agglutination	Guangdong	2019-2020	Human	617	39	Agona	1
							Braenderup	1
							Stanley	1
							Typhimurium	8
							Typhi	1
							Derby	2
							Enteritidis	9
							London	1
							Bradford	1
							Mbandaka	1
							Typhimurium	1
							Typhimurium	12
126	Serum agglutination	Shaanxi	2018-2020	Human	1435	106	Typhimurium	59
							Enteritidis	32
							Stanley	3
							Thompson	2
							Choleraesuis	1
							Others	9
129	Serum agglutination	Guangdong	2020	Human	26253	85	Derby	5
							Stanley	4
							Rissen	5
							Thompson	1
							Bovismorbificans	12
							Litchfield	3
							Enteritidis	1
							Give	1
							Stratford	2
							Calabar	1
							Others	50
135	Serum agglutination	Henan	2016-2020	Human	628	126	Enteritidis	30
							Stanley	18
							Senftenberg	3
							Teshie	3
							Saintpaul	3
							Thompson	3
							Norwich	3
							Bovismorbificans	3
							Braenderup	3
							Typhimurium	24
							Agona	6
							London	3
							Lomita	3
							Eko	3
							Singapore	3
							Kua	3
							Frintrop	3
							Others	9
142	Serum agglutination	Shandong	2020-2021	Human	1300	164	Derby	32
							Choleraesuis	33
							Typhimurium	35
							Enteritidis	64
145	Serum agglutination	Heilongjiang	2016-2019	Human	9977	303	Enteritidis	123
							Typhimurium	59
							Agona	10
							London	8
							Paratyphi B	7
							Derby	7
							Stanley	6
							Infantis	6
							Lagos	5
							Typhi	5

							Rissen	5
							Paratyphi A	4
							Others	58
150	Serum agglutination	Zhejiang	2018-2020	Human	4515	193	Typhimurium	55
							Enteritidis	23
							London	11
							Derby	10
							Tennessee	5
							Bata	4
							Sinstorf	3
							Rissen	3
							Kentucky	3
							Mbandaka	3
							Thompson	3
							Chartres	3
							Derby	2
							Give	2
							Stanley	2
							Indiana	2
							Others	59
168	Serum agglutination	Beijing	2017-2019	Human	1057	66	Enteritidis	25
							Typhimurium	16
							Thompson	4
							Agona	2
							London	2
							Muenchen	2
							Newport	2
							Senftenberg	2
							Paratyphi B	2
							Abaetetuba	1
							Derby	1
							Goldcoast	1
							Herston	1
							Rissen	1
							Saintpaul	1
							Newrochelle	1
							Sinstorf	1
							Arizonae	1
179	Serum agglutination	Beijing	2013-2018	Human	2076	113	Derby	2
							Saintpaul	2
							Thompson	2
							London	3
							Paratyphi B	3
							Agona	5
							Infantis	5
							Senftenberg	8
							Others	16
							Typhimurium	30
							Enteritidis	37
	AIS	Gansu	2020	Food	312	2	Paratyphi A	1
							Typhi	1
192	AIS	Beijing	2015-2019	Human	2104	86	Enteritidis	43
							Typhimurium	13
							Others	30
200	Serum agglutination	Beijing	2014-2019	Human	1870	84	Bovismorbificans	1
							Rissen	1
							Senftenberg	1
							Oslo	2
							Derby	7
							Typhimurium	30
							Enteritidis	42
218	NA	Zhejiang	2019	Human	529	56	Typhimurium	29
							London	6
							Enteritidis	6
							Goldcoast	4
							Rissen	4
							Meleagridis	1
							Escanaba	1
							Infantis	1
							Derby	1
							Newlands	1
							Amina	1
							Choleraesuis	1
233	NA	Beijing	2016-2019	Human	1675	68	Typhimurium	12

							Others	56
239	PFGE	Jiangsu	2018	Human	1246	43	Enteritidis	10
							Dublin	7
							Typhimurium	7
							Agona	2
							Bovismorbificans	2
							London	2
							Senftenberg	2
							Thompson	2
							Akanji	1
							Blegdam	1
							Braenderup	1
							Chester	1
							Koessen	1
							Kentucky	1
							Lexington	1
							Montevideo	1
							Infantis	1
246	Serum agglutination	Tianjin	2018-2020	Human	607	74	Enteritidis	46
							Typhimurium	11
							Albany	3
							Stanley	2
							Goldcoast	2
							Choleraesuis	1
							Litchfield	1
							Gallen	1
							Grampian	1
							Lagos	1
							Mbandaka	1
							Agona	1
							Schwarzengrund	1
							Thompson	1
							Newport	1
250	NA	Beijing	2015-2019	Human	1810	102	Enteritidis	57
							Typhimurium	14
							Braenderup	8
							Others	23
259	Serum agglutination	Yunnan	2017-2020	Human	29674	151	Others	106
							Others	25
							Paratyphi B	3
							Newport	4
							Others	13
261	AIS	Shanghai	2018-2019	Swine	612	1	Typhimurium	1
264	NA	Jiangsu	2019	Human	1036	40	Typhimurium	10
							Enteritidis	6
							Kentucky	4
							Ughelli	4
							Agona	1
							Chailey	1
							Chester	1
							Derby	1
							Rissen	1
							Limete	1
							Mkamba	1
							Senftenberg	1
							Stanley	1
							Ituri	1
							Paratyphi B	1
							Indiana	1
							Infantis	1
							Others	3
271	Serum agglutination	Hebei	2019-2020	Poultry	588	150	Gallinarum	92
							Enteritidis	36
							Paratyphi A	17
							Paratyphi B	2
							Aberdeen	1
							Others	2
292	NA	Jiangxi	2016-2018	Human	5339	170	Typhimurium	57
							Enteritidis	16
							Derby	11
							Rissen	11
							Stanley	10
							Dublin	10
							Thompson	8

							London	8
							Agona	6
							Typhi	4
							Litchfield	3
							Meleagridis	3
							Reading	3
							Paratyphi A	2
							Paratyphi B	2
							Tshiongwe	1
							Paratyphi C	1
							Bardo	1
							Choleraesuis	1
							Give	1
							Goldcoast	1
							Others	10
294	NA	Jilin	2011-2018	Food	13072	75	Enteritidis	28
							Typhimurium	10
							Derby	5
							Senftenberg	4
							Agona	2
							Albany	2
							Arechavaleta	2
							Cardoner	2
							Choleraesuis	2
							Kaapstad	2
							Kubacha	2
							Lomita	2
							London	2
							Potsdam	2
							Rissen	2
							Abaetetuba	1
							Anatum	1
							Assinie	1
							Kingston	1
							Saintpaul	2
303	Serum agglutination	Xinjiang	2019	Food	330	11	Rissen	1
							Derby	6
							Typhimurium	1
							Others	1
							Others	1
							Kiel	1
304	AIS	Jiangsu	2015-2017	Human	389	15	Typhimurium	5
							Enteritidis	4
							Others	6
309	Serum agglutination	Zhejiang	2007-2018	Human	3327	288	Agona	7
							Poona	7
							Bovismorbificans	9
							Newport	9
							Rissen	10
							London	12
							Derby	16
							Stanley	24
							Enteritidis	28
							Others	52
							Typhimurium	114
315	Serum agglutination	Shanghai	2018-2019	Food	308	122	Corvallis	18
							London	15
							Rissen	14
							Derby	13
							Agona	11
							Kentucky	8
							Indiana	7
							Enteritidis	6
							Typhimurium	5
							Meleagridis	4
							Mbandaka	4
							Typhimurium	3
							Braenderup	3
							Haifa	2
							Goldcoast	2
							Give	2
							Thompson	1
							Saintpaul	1
							Livingstone	1

							Kottbus	1
							Barranquilla	1
323	AIS	Shandong	2018-2019	Human	452	120	Enteritidis	65
							Typhimurium	9
							Others	46
329	NA	Shanghai	2015-2018	Human	2871	94	Typhimurium	1
							Enteritidis	2
							Stanley	1
							Senftenberg	1
							Typhimurium	3
							Others	86
348	Serum agglutination	Jilin	2019	Poultry	1024	148	Paratyphi A	8
							Gallinarum	28
							Typhimurium	10
							Stanley	11
							Choleraesuis	8
							London	7
							Derby	9
							Bovismorbificans	10
							Rissen	7
							Typhiavian	24
							Others	26
355	NA	Shanghai	2014-2019	Food	5667	300	Anatum	1
							Blegdam	1
							Braenderup	1
							Chester	1
							Eingedi	1
							Irumu	1
							Litchfield	1
							Livingstone	1
							Muenster	1
							Newlands	1
							Pakistan	1
							Potsdam	1
							Putten	1
							Weltevreden	1
							Agona	2
							Bovismorbificans	2
							Give	2
							Senftenberg	2
							Virchow	2
							Stanley	3
							Aberdeen	4
							Kentucky	5
							Montevideo	5
							Newport	5
							Mattenhof	6
							Corvallis	7
							Goldcoast	7
							Indiana	7
							Meleagridis	9
							Thompson	16
							Enteritidis	27
							Rissen	30
							London	44
							Derby	45
							Typhimurium	56
356	Serum agglutination	Jiangsu	2019	Human	324	85	Derby	27
							Enteritidis	17
							Typhimurium	15
							London	6
							Rissen	6
							Senftenberg	2
							Kentucky	2
							Anatum	2
							Others	8
373	NA	Zhejiang	2015-2018	Human	1933	205	Typhimurium	89
							Others	85
							Enteritidis	31
400	Serum agglutination	Sichuan	2012-2019	Human	1829	160	London	1
							Derby	2
							Farsta	2
							Goldcoast	2
							Thompson	2

							Blegdam	3
							London	3
							Enteritidis	16
							Others	21
							Typhimurium	108
411	NA	Tianjin	2017-2018	Human	1067	31	Enteritidis	12
							Typhimurium	6
							Albany	4
							Infantis	1
							Goldcoast	1
							Mbandaka	1
							Bovismorbificans	2
							Litchfield	1
							Others	3
417	Serum agglutination	Jiangsu	2016-2018	Poultry	329	49	Derby	2
							Typhimurium	3
							Indiana	11
							Agona	8
							Rissen	1
							Mbandaka	2
							Potsdam	1
							Kentucky	2
							Enteritidis	3
							Anatum	6
							Newlands	4
							Meleagridis	1
							Senftenberg	2
							Others	3
426	Serum agglutination	Tibet	2019	Swine	300	33	London	25
							Typhimurium	6
							Others	2
427	Serum agglutination	Anhui	2010-2018	Human	2998	265	Agona	67
							Enteritidis	54
							Others	91
							Typhimurium	53
442	Serum agglutination	Jiangsu	2019	Human	3582	105	Typhimurium	44
							Grampian	19
							Montevideo	5
							Dublin	4
							Kentucky	4
							Stanley	4
							Others	25
446	Serum agglutination	Jiangsu	2014-2018	Human	1943	249	Goldcoast	5
							Infantis	5
							Poona	5
							Bovismorbificans	9
							Rissen	9
							London	10
							Newport	10
							Derby	17
							Enteritidis	20
							Others	58
							Typhimurium	101
447	Serum agglutination	Guangdong	2016-2018	Poultry	435	17	Enteritidis	13
							Thompson	2
							Indiana	2
451	NA	Beijing	2014-2017	Human	1520	95	Bovismorbificans	1
							Dublin	1
							Mbandaka	1
							Newlands	1
							Newlands	1
							Paratyphi B	1
							Agona	2
							Braenderup	2
							Stanley	4
							Infantis	5
							London	5
							Senftenberg	5
							Stanley	6
							Typhimurium	8
							Typhimurium	10
							Enteritidis	42
453	NA	Shanghai	2018	Human	5580	234	Enteritidis	95
							Typhimurium	29

							Braenderup	5
							London	5
							Goldcoast	3
							Newport	2
							Senftenberg	2
							Thompson	2
							Paratyphi B	2
							Infantis	2
							Others	87
457	Serum agglutination	Xinjiang	2017-2019	Human	1200	641	Senftenberg	240
							Enteritidis	188
							Thompson	170
							Typhimurium	43
462	Serum agglutination	Zhejiang	2017-2019	Human	1363	43	Typhimurium	6
							Enteritidis	21
							Newport	2
							Derby	1
							Agona	1
							Rissen	1
							Others	11
467	Serum agglutination	Yunnan	2018-2019	Human	8558	320	Enteritidis	163
							Typhimurium	54
							Paratyphi B	38
							Typhimurium	24
							Paratyphi A	14
							Typhi	5
							Derby	4
							Kentucky	3
							London	3
							Thompson	3
							Paratyphi C	2
							Dublin	2
							Stanley	2
							Manhattan	1
							Saintpaul	1
							Choleraesuis	1
470	Serum agglutination	Henan	2019	Human	495	78	Enteritidis	34
							Typhimurium	22
							Thompson	8
							Stanley	2
							Goldcoast	2
							London	2
							Ughelli	1
							Anatum	1
							Indiana	1
							Dublin	1
							Sandiego	1
							Others	3
478	Serum agglutination	Hubei	2018	Human	640	39	Typhimurium	27
							Derby	6
							Enteritidis	1
							London	1
							Others	4
503	Serum agglutination	Guangdong	2017-2019	Human	2312	343	Typhimurium	179
							Enteritidis	41
							Stanley	37
							Rissen	18
							Derby	6
							Saintpaul	6
							Virchow	6
							Goldcoast	5
							London	5
							Weltevreden	5
							Agona	3
							Braenderup	2
							Typhi	1
							Others	29
509	Serum agglutination	Anhui	2014-2018	Food	717	11	Schwarzengrund	2
							Enteritidis	2
							Nitra	1
							Tsevie	1
							Agona	1
							Others	2
							Thompson	1

							Tshiongwe	1
515	NA	Guangxi	2018	Human	8702	286	Enteritidis	67
							Typhimurium	43
							Lagos	24
							Dublin	15
							Blegdam	15
							Thompson	10
							Agona	9
							London	7
							Kottbus	7
							Others	89
561	Serum agglutination	Shaanxi	2018	Human	2294	101	Others	9
							Typhimurium	57
							Enteritidis	19
							London	4
							Derby	4
							Thompson	3
							Stanley	3
							Kentucky	1
							Agona	1
563	Serum agglutination	Zhejiang	2015-2017	Human	932	208	Apeyeme	1
							Corvallis	1
							Goldcoast	1
							Hindmarsh	1
							Poona	1
							Rissen	1
							Saintpaul	1
							Senftenberg	1
							Agona	2
							Give	2
							Redba	2
							Virchow	2
							Wandsworth	2
							Bovismorbificans	3
							Newport	5
							London	6
							Derby	14
							Enteritidis	17
							Stanley	20
							Others	51
							Typhimurium	74
571	Serum agglutination	Shanghai	2013-2017	Human	9301	305	Enteritidis	120
							Typhimurium	103
							Others	82
576	Serum agglutination	Guangxi	2014-2017	Human	408	22	Livingstone	1
							Saintpaul	1
							Goldcoast	2
							Newport	2
							Typhimurium	2
							Weltevreden	2
							Corvallis	3
							Rissen	4
							Derby	5
577	Serum agglutination	Guangdong	2015-2017	Human	12890	532	Manhattan	1
							Senftenberg	1
							London	2
							Meleagridis	2
							Kottbus	3
							Newport	3
							Bovismorbificans	9
							Infantis	10
							Potsdam	12
							Saintpaul	14
							Stanley	16
							Derby	23
							Enteritidis	81
							Typhimurium	355
579	Serum agglutination	Guangdong	2016	Human	345	55	Typhimurium	36
							Stanley	6
							Rissen	3
							Derby	2
							Typhi	1
							Enteritidis	1
							London	1

							Weltevreden	1
							Others	4
586	Serum agglutination	Beijing	2014-2018	Human	1827	89	Infantis	2
							Kilva	2
							London	2
							Manhattan	2
							Papuana	2
							Derby	3
							Braenderup	4
							Choleraesuis	4
							Paratyphi B	4
							Senftenberg	4
							Blegdam	7
							Typhimurium	11
							Others	19
							Enteritidis	23
654	NA	Shanghai	2015-2018	Human	3845	139	Enteritidis	41
							Typhimurium	25
							Others	73
657	Serum agglutination	Guangxi	2018	Human	401	29	Typhimurium	18
							Others	11
666	Serum agglutination	Guangdong	2017-2018	Human	1219	128	Typhimurium	58
							Enteritidis	15
							Stanley	8
							Derby	7
							London	6
							Others	28
							Others	6
684	Serum agglutination	Zhejiang	2014-2018	Human	2327	299	Bovismorbificans	10
							Newport	12
							Rissen	12
							London	15
							Derby	19
							Enteritidis	27
							Stanley	30
							Others	50
							Typhimurium	124
704	Serum agglutination	Hainan	2016-2018	Human	6837	766	Typhimurium	328
							Typhimurium	98
							Enteritidis	131
							Stanley	38
							Agona	21
							Derby	29
							Rissen	20
							Hadar	4
							Goldcoast	14
							Infantis	4
							Others	79
705	Serum agglutination	Shaanxi	2016-2018	Human	1329	28	Derby	4
							Agona	1
							Typhimurium	15
							Heidelberg	1
							Sandiego	1
							Blegdam	1
							Dublin	1
							Enteritidis	4
719	Serum agglutination	Beijing	2017-2018	Human	380	19	Enteritidis	7
							Stanley	3
							Senftenberg	2
							Tshiongwe	1
							Calabar	1
							Kentucky	1
							Mbandaka	1
							Paratyphi B	1
							Infantis	1
							Agona	1
726	AIS	Beijing	2018	Human	380	23	Enteritidis	13
							Stanley	3
							London	2
							Typhimurium	2
							Irumu	1
							Agona	1
							Give	1
738	Serum agglutination	Anhui	2013-2015	Human	2618	76	Typhimurium	28
							Enteritidis	22
							Braenderup	6
							Derby	4
							Thompson	3
							Newport	2

								Stanley	2
								Agona	1
								Bovismorbificans	1
								Kottbus	1
								Singapore	1
								Fillmore	1
								Infantis	1
								Derby	1
								Rissen	1
								Javiana	1
750	Serum agglutination	Guangdong	2015-2018	Human	2619	594		Enteritidis	268
								Others	172
								Stanley	85
								Typhimurium	69
764	Serum agglutination	Yunnan	2016-2018	Human	60533	186		Farsta	1
								Sloterdijk	2
								Essen	2
								Texas	1
								Neumuenster	1
								Ball	1
								Typhimurium	34
								Bradford	1
								Remo	2
								Tafo	1
								Stanley	3
								Agona	6
								Derby	12
								Choleraesuis	3
								Rissen	8
								Eingedi	1
								Thompson	2
								Tallahassee	2
								Edmonton	2
								Corvallis	2
								Fillmore	2
								Kentucky	2
								Bellevue	1
								Enteritidis	28
								Blegdam	3
								Assinie	5
								Give	7
								Meleagridis	2
								Newlands	3
								London	17
								Coquilhatville	1
								Bonariensis	1
								Anatum	2
								Weltevreden	1
								Goelzau	2
								Krefeld	7
								Fulda	7
								Kaapstad	3
								Others	5
766	Serum agglutination	Zhejiang	2018	Poultry	596	78		Typhimurium	31
								Indiana	23
								Cerro	1
								Ferruch	1
								Kentucky	1
								Kottbus	1
								Ngor	1
								Others	19
772	Serum agglutination	Hunan	2017-2018	Swine	300	37		Typhimurium	30
								Derby	1
								Rissen	1
								Others	5
		Hunan	2017-2018	Poultry	170	32		Enteritidis	1
								Typhimurium	18
								Gallinarum	10
								Others	3
778	Serum agglutination	Jiangsu	2015-2017	Food	339	15		Infantis	1
								Agona	1
								Braenderup	1
								Chailey	1
								Infantis	1
								Kedougou	1
								Kisangani	1
								Others	6
								Stanleyville	1
								Teshie	1
		Jiangsu	2015-2017	Human	1606	79		Derby	1
								London	1
								Muenchen	1
								Stanley	1
								Stanleyville	1

							Others	3
							Enteritidis	3
							Typhimurium	3
							Rissen	4
							Saintpaul	4
							Kottbus	5
							Infantis	7
							Lagos	27
							Dublin	18
784	Serum agglutination	Chongqing	1991-2017	Human	33957	319	Dublin	21
							Others	52
							Paratyphi B	12
							Typhi	13
							Typhimurium	221
794	Serum agglutination	Zhejiang	2013	Human	517	31	Enteritidis	7
							Typhimurium	6
							Bovismorbificans	3
							Anatum	2
							Rissen	2
							Dublin	2
							Litchfield	1
							Schwarzengrund	1
							Paratyphi B	1
							Albany	1
							Agama	1
							Blegdam	1
							Braenderup	1
							Chailey	1
							Derby	1
798	Serum agglutination	Sichuan	2012-2017	Human	5934	340	Choleraesuis	6
							Dublin	22
							Enteritidis	8
							London	2
							Meleagridis	3
							Newport	12
							Taksony	5
							Thompson	5
							Typhimurium	277
800	Serum agglutination	Guangdong	2016-2017	Human	905	120	Typhimurium	92
							Enteritidis	15
							Saintpaul	2
							Agona	1
							Blegdam	1
							Kentucky	1
							Paratyphi B	1
							London	1
							Rissen	1
							Javiana	1
							Stanley	1
							Sandiego	1
							Singapore	1
							Others	1
816	Serum agglutination	Henan	2018	Human	2083	12	Enteritidis	3
							London	2
							Senftenberg	1
							Eingedi	1
							Kentucky	2
							Derby	1
							Douala	1
							Svedvi	1
833	Serum agglutination	Guangdong	2012-2017	Poultry	129	25	Indiana	1
							Infantis	1
							Meleagridis	1
							Agona	2
							Chester	2
							Corvallis	2
							Derby	2
							Emek	2
							Kentucky	2
							Stanley	3
							Typhimurium	7
		Guangdong	2012-2017	Food	501	58	Typhimurium	12
							Derby	11
							London	12
							Rissen	9
							Stanley	2
							Meleagridis	2
							Agona	1
							Enteritidis	2
							Litchfield	2
							Chester	2
							Newlands	1
							Senftenberg	1
							Others	1

834	PCR, Serum agglutination	Jiangsu	2015-2018	Poultry	5889	56	Choleraesuis	14
							Enteritidis	7
							Potsdam	5
							Typhimurium	30
863	Serum agglutination	Henan	2018	Swine	840	45	Derby	17
							Reading	5
							Typhimurium	4
							Rissen	5
							Kentucky	11
							London	1
							Others	2
874	Serum agglutination	Beijing	2016-2018	Human	1269	58	Enteritidis	18
							Typhimurium	6
							Typhimurium	4
							Agona	4
							Mbandaka	3
							Altendorf	2
							Braenderup	2
							Senftenberg	2
							Others	17
875	Serum agglutination	Jiangsu	2015-2018	Human	1000	100	Chester	1
							Cremieu	1
							Eppendorf	1
							Isangi	1
							Lomita	1
							Meleagridis	1
							Naestved	1
							Newlands	1
							Nitra	1
							Others	1
							Regent	1
							Rissen	1
							Singapore	1
							Eko	2
							Gallinarum	2
							Infantis	2
							Kottbus	2
							Muenster	2
							Potsdam	2
							Sandiego	2
							London	3
							Senftenberg	3
							Manhattan	5
							Reading	5
							Agona	6
							Enteritidis	6
							Paratyphi A	6
							Thompson	7
							Derby	9
							Newport	10
							Anatum	13
882	Serum agglutination	Guangxi	2015-2017	Poultry	1554	55	Paratyphi A	6
							Typhimurium	23
							Newport	1
							Gallinarum	25
884	Serum agglutination	Liaoning	2017-2018	Swine	412	39	Choleraesuis	16
							Enteritidis	15
							Typhimurium	2
							Others	6
885	Serum agglutination	Henan	2015-2018	Poultry	43	24	Typhimurium	4
							Derby	2
							Indiana	1
							Escanaba	1
							Others	1
							Corvallis	9
							Dabou	2
							Kentucky	1
							Enteritidis	2
							Others	1
		Henan	2015-2018	Food	21	5	Chester	3
							Blegdam	1
							Others	1
		Henan	2015-2018	Cold-blooded animals	124	12	Alamo	3
							Escanaba	2
							Muenchen	1
							Corvallis	1
							Enteritidis	1
							Kallo	1
							Madelia	1
							Havana	1
							London	1

887	Serum agglutination	Zhejiang	2017-2018	Food	2039	37	Typhimurium	10
							Derby	6
							Enteritidis	4
							Dabou	3
							London	2
							Kentucky	2
							Dublin	1
							Meleagridis	1
							Saintpaul	1
							Give	1
							Agona	1
							Braenderup	1
							Ruzizi	1
							Kottbus	1
							Newport	1
							Stanley	1
896	Serum agglutination	Beijing	2013-2015	Human	1672	58	Derby	3
							London	3
							Paratyphi B	4
							Senftenberg	4
							Others	10
							Typhimurium	13
							Enteritidis	21
908	Serum agglutination	Hebei	2015-2017	Food	680	24	Kentucky	1
							Meleagridis	1
							Thompson	1
							Typhi	1
							Typhimurium	1
							Agona	2
							Bovismorbificans	2
							Mbandaka	2
							Stanley	2
							Enteritidis	3
							London	3
							Rissen	5
923	Serum agglutination	Beijing	2016-2017	Human	1123	26	Enteritidis	6
							Stanley	4
							Paratyphi B	4
							Others	12
924	Serum agglutination	Guangxi	2018	Human	1238	63	Enteritidis	22
							Typhimurium	16
							Agona	14
							Derby	6
							Choleraesuis	3
							Braenderup	2
946	Serum agglutination	Jiangsu	2016-2017	Human	982	32	Typhimurium	11
							Enteritidis	4
							Derby	3
							Give	2
							Newlands	1
							Anatum	1
							London	1
							Meleagridis	1
							Paratyphi A	1
							Southampton	1
							Braenderup	1
							Stanley	1
							Brikama	1
							Rissen	1
							Gloucester	1
							Sinstorf	1
961	Serum agglutination	Beijing	2017	Human	515	18	Enteritidis	3
							Paratyphi B	3
							Typhimurium	2
							Essen	1
							Bovismorbificans	1
							Anatum	1
							Litchfield	1
							Thompson	1
							Senftenberg	1
							London	1
							Agona	1
							Others	1
							Wien	1
963	Serum agglutination	Shanghai	2015-2018	Human	333	18	Agona	1
							Gaminara	1
							Kentucky	1
							Thompson	1
							Rissen	2
							Enteritidis	6
							Typhimurium	6
964	Serum agglutination	Guangdong	2014-2017	Human	2685	180	Agona	5
							Enteritidis	34
							Others	60

							Typhimurium	81
984	Serum agglutination	Ningxia	2010-2016	Human	1175	51	Agona	1
							Bovismorbificans	1
							Enteritidis	27
							Lomita	1
							London	1
							Others	1
							Rissen	1
							Stanley	1
							Typhimurium	15
							Virchow	2
991	Serum agglutination	Shanghai	2018	Poultry	615	70	Gallinarum	13
							Enteritidis	57
997	NA	Shanxi	2015-2017	Human	752	17	Enteritidis	6
							Typhimurium	4
							Thompson	6
							Poona	1
1001	Serum agglutination	Shaanxi	2018	Human	594	58	Derby	2
							Typhimurium	29
							Stanleyville	3
							Thompson	2
							Newport	1
							Potsdam	1
							Others	1
							Enteritidis	13
							Panama	1
							London	2
							Others	2
							Wandsworth	1
1002	Serum agglutination	Henan	2017	Human	504	100	Typhimurium	38
							Enteritidis	29
							Thompson	8
							Kentucky	4
							London	3
							Muenster	2
							Afula	1
							Agama	1
							Agona	1
							Tsevie	1
							Farsta	1
							Fillmore	1
							Goldcoast	1
							Rissen	1
							Poona	1
							Mikawashima	1
							Senftenberg	1
							Stanley	1
							Infantis	1
							Others	3
1009	Serum agglutination	Zhejiang	2016	Human	49774	1102	Typhimurium	396
							Enteritidis	134
							Others	572
1013	Serum agglutination	Zhejiang	2013-2017	Human	1664	78	Derby	1
							Newlands	1
							Paratyphi B	1
							Senftenberg	1
							Thompson	1
							London	2
							Manhattan	2
							Newport	2
							Potsdam	2
							Sinchew	2
							Typhi	2
							Choleraesuis	3
							Others	8
							Dublin	12
							Typhimurium	38
1014	Serum agglutination	Guangdong	2015-2017	Human	4019	108	Derby	1
							Essen	1
							Kisangani	1
							Paratyphi B	1
							Paratyphi C	1
							Sendai	1
							Typhi	1
							Agona	2
							Blegdam	2
							London	2
							Gallinarum	3
							Thompson	3
							Tsevie	4
							Dublin	10
							Enteritidis	12
							Stanley	17
							Typhimurium	46

1024	Serum agglutination	Shanxi	2015-2016	Human	2505	51	Assinie	1
							Give	1
							Weston	1
							Derby	2
							Paratyphi A	2
							Paratyphi B	2
							Rissen	2
							Senftenberg	3
							Newport	4
							Typhimurium	15
							Enteritidis	18
1030	Serum agglutination	Shanghai	2013-2017	Human	16860	222	Enteritidis	90
							Others	75
							Typhimurium	57
1032	Serum agglutination	Guangdong	2013-2015	Human	3080	402	Typhimurium	402
1040	Serum agglutination	Beijing	2017	Human	352	10	Enteritidis	4
							Stanley	1
							Typhimurium	1
							Senftenberg	1
							Others	3
1043	Serum agglutination	Liaoning	2017	Poultry	632	131	Enteritidis	104
							Duisburg	27
1046	Serum agglutination	Guangdong	2015-2017	Human	3757	271	Manhattan	1
							Paratyphi C	1
							Potsdam	1
							Gallinarum	2
							Kottbus	2
							London	2
							Choleraesuis	3
							Others	3
							Derby	7
							Saintpaul	8
							Agona	9
							Infantis	9
							Stanley	11
							Enteritidis	31
							Typhimurium	181
1056	AIS	Qinghai	2015-2016	Human	817	17	Typhi	2
							Typhimurium	12
							Others	3
1074	Serum agglutination	Sichuan	2016-2017	Human	36451	43	Typhimurium	2
							Senftenberg	3
							Infantis	7
							Agona	2
							Derby	8
							Thompson	12
							Manhattan	9
1079	Serum agglutination	Henan	2011-2013	Poultry	1699	149	Aberdeen	2
							Agona	4
							Bata	2
							Corvallis	3
							Derby	10
							Enteritidis	73
							Essen	1
							Hadar	9
							Indiana	20
							Infantis	1
							Istoria	3
							London	1
							Senftenberg	2
							Shubra	2
							Stanley	2
							Typhimurium	11
							Zanzibar	2
							Others	1
		Henan	2011-2013	Swine	1382	138	Enteritidis	5
							Derby	52
							Typhimurium	33
							Indiana	3
							Agona	10
							Senftenberg	7
							Meleagridis	7
							London	5
							Corvallis	1
							Thompson	1
							Beta	1
							Shubra	1
							Others	12
		Henan	2011-2013	Ruminants	690	24	Enteritidis	1
							Derby	5
							Typhimurium	5
							Agona	1
							Senftenberg	4

							London	3
							Corvallis	1
							Infantis	1
							Essen	1
							Others	2
		Henan	2011-2013	Food	532	20	Derby	3
							Indiana	1
							Agona	1
							Hadar	1
							Aberdeen	2
							Thompson	3
							Others	9
1081	Serum agglutination	Henan	2017	Poultry	351	62	Pullorum	40
							Tennessee	9
							Thompson	4
							Kimpese	4
							Cerro	4
							Entebbe	1
1094	Serum agglutination	Inner Mongolia	2017	Ruminants	317	30	Paratyphi A	1
							Typhimurium	10
							Choleraesuis	3
							Paratyphi B	10
							Paratyphi C	2
							Thompson	1
							Derby	3
1095	Serum agglutination	Shandong	2015	Swine	1480	298	Derby	78
							Typhimurium	59
							Thompson	45
							Agona	40
							Enteritidis	25
							Lomita	18
							Tsevie	15
							Lagos	12
							Oritamerin	6
1100	Serum agglutination	Shandong	2016-2017	Human	2400	27	Enteritidis	27
1103	Serum agglutination	Sichuan	2015-2017	Food	376	32	Sandiego	2
							Warnow	1
							Kentucky	1
							Bangui	2
							Others	1
							Senftenberg	23
							Cerro	2
1127	Serum agglutination	Beijing	2015	Human	371	25	Enteritidis	6
							Agona	1
							Typhimurium	1
							Blegdam	1
							Braenderup	1
							Kilva	1
							California	1
							Enteritidis	7
							Agona	2
							Typhimurium	1
							Muenchen	1
							Thompson	1
							Wandsworth	1
1140	NA	Zhejiang	2015-2017	Human	1258	25	Bovismorbificans	1
							Poona	1
							Rissen	1
							Enteritidis	3
							Oranienburg	3
							London	6
							Typhimurium	10
1148	Serum agglutination	Hebei	2017	Food	570	7	Choleraesuis	1
							Agona	2
							Derby	2
							Bovismorbificans	1
							Stanley	1
1150	Serum agglutination	Henan	2017	Poultry	452	97	Enteritidis	2
							Hadar	1
							Enteritidis	62
							Infantis	6
							Hadar	11
							Indiana	10
							Senftenberg	3
							Others	2
1159	AIS	Zhejiang	2014-2016	Human	883	17	Typhimurium	11
							Others	6
1175	Serum agglutination	Jiangsu	2015-2016	Poultry	189	24	Indiana	2
							Senftenberg	2
							Braenderup	3
							Typhimurium	5
							Enteritidis	12
		Jiangsu	2015-2016	Swine	116	12	Enteritidis	4

							Typhimurium	3
							Anatum	1
							Indiana	1
							London	2
							Thompson	1
		Jiangsu	2015	Ruminants	63	1	Typhimurium	1
1180	Serum agglutination	Sichuan	2018	Poultry	343	99	Indiana	13
							Newlands	10
							Anatum	9
							London	2
							Typhi	2
							Typhimurium	3
							Indiana	17
							Newlands	18
							Anatum	5
							London	7
							Typhi	3
							Agona	2
							Others	8
1186	Serum agglutination	Hebei	2016	Human	36452	6	Agona	1
							Typhimurium	2
							Rissen	1
							Bovismorbificans	1
							Typhi	1
1189	Serum agglutination	Shandong	2009-2016	Food	115	26	Agona	1
							Bonn	1
							Braenderup	1
							Elizabethville	1
							Infantis	1
							Mbandaka	1
							Muenster	1
							Senftenberg	1
							Virchow	1
							Choleraesuis	2
							London	2
							Others	2
							Derby	11
		Shandong	2009-2016	Poultry	227	83	Aba	1
							Bonn	1
							Derby	1
							Kottbus	1
							Lindenberg	1
							Mbandaka	1
							Nigeria	1
							Redba	1
							Regent	1
							Southampton	1
							Stanley	1
							Aba	2
							Chailey	2
							Typhimurium	3
							Others	7
							Indiana	25
							Enteritidis	33
1190	Serum agglutination	Guangdong	2014-2016	Human	90887	24	Agona	1
							Enteritidis	1
							Israel	1
							Reading	1
							Senftenberg	1
							Chester	2
							Clerkenwell	2
							London	2
							Newlands	2
							Rissen	2
							Typhimurium	2
							Stanley	3
							Derby	4
1194	Serum agglutination	Guizhou	2013-2014	Human	432	35	Enteritidis	11
							Typhimurium	9
							Clerkenwell	6
							Stanley	3
							Derby	2
							Agona	1
							Infantis	1
							Bovismorbificans	1
							Others	1
1213	Serum agglutination	Beijing	2014-2017	Human	1453	76	Chester	1
							Derby	1
							Kottbus	1
							Mississippi	1
							Newrochelle	1
							Rissen	1
							Saintpaul	1

							Sinstorf	1
							Stanley	1
							Stanleyville	1
							Tennessee	1
							Thompson	1
							Uganda	1
							London	2
							Senftenberg	3
							Agona	4
							Indiana	4
							Enteritidis	13
							Paratyphi B	13
							Typhimurium	24
1225	Serum agglutination	Shanghai	2016-2017	Human	400	22	Typhimurium	8
							Enteritidis	6
							London	4
							Derby	1
							Thompson	1
							Newport	1
							Give	1
1235	Serum agglutination	Henan	2015	Poultry	554	77	Indiana	32
							Enteritidis	37
							Hadar	6
							Remo	1
							Shubra	1
1248	Serum agglutination	Jiangsu	2016	Human	417	5	Typhimurium	1
							Enteritidis	2
							Oranienburg	1
							Others	1
1262	Serum agglutination	Jiangsu	2010-2016	Food	719	53	Bovismorbificans	1
							Thompson	1
							London	2
							Others	2
							Anatum	3
							Indiana	3
							Senftenberg	3
							Braenderup	4
							Typhimurium	12
							Enteritidis	22
1271	Serum agglutination	Beijing	2016	Human	340	21	Kentucky	8
							Enteritidis	3
							Typhimurium	3
							Blegdam	2
							Papuana	1
							Thompson	1
							Montevideo	1
							Litchfield	1
							Albany	1
1281	Serum agglutination	Beijing	2013-2017	Human	1455	73	Blegdam	1
							Braenderup	1
							Infantis	1
							Lagos	1
							Paratyphi B	1
							Saintpaul	1
							Stanleyville	1
							Tennessee	1
							Thompson	1
							Uganda	1
							Others	2
							Indiana	3
							Rissen	3
							Agona	4
							Derby	4
							Senftenberg	6
							Typhimurium	20
							Enteritidis	21
1288	Serum agglutination	Guangxi	2016	Food	12120	70	Typhimurium	10
							Kentucky	5
							Derby	5
							Agona	4
							Others	46
		Guangxi	2016	Human	5400	234	Typhimurium	100
							Enteritidis	33
							Stanley	21
							Agona	8
							Derby	5
							Others	67
1294	Serum agglutination	Xinjiang	2013-2016	Human	1552	29	Typhimurium	3
							Enteritidis	20
							Others	6
1295	Serum agglutination	Yunnan	2015	Human	10342	56	Farsta	3
							Sloterdijk	2
							Essen	1
							Texas	1

							Neumuenster	1
							Ball	1
							Typhimurium	13
							Bradford	1
							Remo	1
							Tafo	1
							Stanley	1
							Others	3
							Choleraesuis	2
							Rissen	1
							Tallahassee	2
							Edmonton	1
							Corvallis	1
							Fillmore	1
							Enteritidis	5
							Assinie	4
							Give	1
							Meleagridis	1
							Newlands	2
							Krefeld	5
							Others	1
1308	Serum agglutination	Guangdong	2014-2017	Human	4847	192	Ruzizi	3
							Senftenberg	7
							Stanley	9
							Enteritidis	29
							Others	34
							Typhimurium	110
1310	Serum agglutination	Guangdong	2016	Human	7673	15	Derby	8
							Enteritidis	3
							Typhimurium	2
							Paratyphi A	1
							Anatum	1
1311	Serum agglutination	Beijing	2012-2017	Human	16349	677	Newport	10
							London	12
							Braenderup	13
							Derby	13
							Infantis	28
							Agona	30
							Senftenberg	61
							Others	114
							Typhimurium	156
							Enteritidis	240
1314	Serum agglutination	Shanghai	2011-2014	Food	600	154	Indiana	24
							Derby	22
							Enteritidis	48
							Typhimurium	28
							Thompson	6
							Tshiongwe	3
							Paratyphi B	1
							Aberdeen	1
							Kottbus	1
							Potsdam	1
							Bredeney	2
							Agona	1
							Newport	3
							Rissen	1
							Senftenberg	1
							Gallinarum	2
							Remo	3
							Manchester	2
							London	3
							Infantis	1
1333	Serum agglutination	Guangdong	2015-2017	Human	1136	108	Stanley	4
							Typhi	5
							Gallinarum	6
							Others	11
							Typhimurium	82
1339	Serum agglutination	Henan	2014-2015	Poultry	695	263	Cerro	13
							Edinburg	17
							Thompson	14
							Tennessee	9
							Kimpese	4
							Entebbe	1
							Gatuni	1
							Tamilnadu	1
							Pullorum	203
1345	Serum agglutination	Guangdong	2015	Human	4620	61	Stanley	8
							Typhimurium	6
							Derby	7
							Agona	4
							Paratyphi B	1
							Others	5
							Thompson	4
							Infantis	2

							Potsdam	1
							Others	5
							Newport	4
							Bovismorbificans	1
							Manhattan	1
							Others	1
							London	3
							Newlands	1
							Others	3
							Senftenberg	4
1354	Serum agglutination	Guangdong	2016	Poultry	316	243	Agona	48
							Corvallis	34
							Mbandaka	29
							Kentucky	25
							Enteritidis	19
							Braenderup	18
							Others	70
1359	Serum agglutination	Jiangsu	2017	Swine	1727	184	Derby	101
							Typhimurium	30
							Agona	20
							Rissen	13
							Anatum	5
							Infantis	2
							Newlands	4
							Sinchew	2
							Fillmore	1
							Redba	1
							Eingedi	1
							Others	4
1362	AIS	Beijing	2015-2017	Human	1335	126	Braenderup	3
							Infantis	3
							Senftenberg	3
							Derby	4
							Agona	6
							Dublin	15
							Typhimurium	24
							Others	30
							Enteritidis	38
1371	Serum agglutination	Shandong	2011-2017	Food	1681	122	Others	1
							Dublin	2
							Thompson	8
							Gallinarum	9
							Rissen	15
							Enteritidis	31
							Indiana	56
1389	Serum agglutination	Beijing	2010-2015	Human	3428	156	Infantis	6
							Agona	13
							Others	39
							Typhimurium	44
							Enteritidis	54
1407	Serum agglutination	Shandong	2010-2015	Human	513	31	Farsta	1
							Galiema	1
							Loanda	1
							Senftenberg	1
							Dublin	2
							Derby	3
							Typhimurium	5
							Infantis	6
							Enteritidis	11
1413	Serum agglutination	Henan	2016	Swine	840	67	Derby	32
							Typhimurium	31
							Give	2
							Reading	1
							Tumodi	1
1419	Serum agglutination	Guangdong	2014-2015	Human	412	50	Enteritidis	12
							Senftenberg	5
							Stanley	5
							Typhimurium	4
							Litchfield	4
							Others	1
							Essen	1
							Papuana	1
							Derby	1
							Fillmore	1
							Ruzizi	1
							London	1
							Manchester	1
							Uganda	1
							Others	11
1428	Serum agglutination	Beijing	2015-2016	Human	3068	33	Agona	1
							Derby	1
							Menden	1
							Rissen	1
							Colorado	2

							Infantis	2
							Thompson	2
							Wien	3
							Enteritidis	10
							Typhimurium	10
1440	Serum agglutination	Sichuan	2017	Human	15613	6	Thompson	1
							Manhattan	2
							Derby	1
							Agona	1
							Braenderup	1
1458	Serum agglutination	Anhui	2012-2014	Dogs	746	17	Enteritidis	7
							Others	4
							Derby	3
							Thompson	1
							Blegdam	1
							Indiana	1
		Anhui	2012-2014	Feed	404	10	Enteritidis	1
							Derby	5
							Braenderup	1
							Others	2
							Others	1
1474	AIS	Henan	2015	Human	2824	79	Enteritidis	36
							Typhimurium	12
							Thompson	5
							Agona	1
							Indiana	1
							Indiana	24
		Henan	2016	Human	2896	142	Enteritidis	66
							Typhimurium	22
							Thompson	8
							Agona	1
							Indiana	2
							Indiana	43
1482	NA	Hunan	2016	Food	3647	121	Heidelberg	29
							Typhimurium	25
							Kentucky	26
							Pakistan	5
							Infantis	6
							Enteritidis	19
							Anatum	2
							Thompson	2
							Mbandaka	1
							Montevideo	1
							Others	2
							Schwarzengrund	1
							Derby	1
							Hadar	1
1507	Serum agglutination	Jiangsu	2014-2016	Food	4186	112	Derby	24
							Aberdeen	1
							Bardo	1
							Braenderup	1
							Chester	1
							Pakistan	1
							Thompson	1
							Dublin	2
							Irumu	2
							Newport	2
							Paratyphi A	2
							Reading	2
							London	3
							Anatum	4
							Choleraesuis	4
							Meleagridis	7
							Typhimurium	7
							Derby	12
							Enteritidis	14
							Agona	21
1533	AIS	Shaanxi	2008-2014	Human	2113	34	Senftenberg	3
							Gallinarum	4
							Thompson	12
							Typhimurium	12
							Others	3
1554	PCR	Beijing	2013-2015	Human	947	52	Typhimurium	10
							Derby	6
							Senftenberg	6
							Infantis	3
							Rissen	2
							Others	7
							Enteritidis	18
1567	Serum agglutination	Jiangxi	2015	Human	300	10	Enteritidis	6
							Typhimurium	3
							Bovismorbificans	1
		Jiangxi	2016	Human	300	9	Enteritidis	5
							Typhimurium	2

							Bovismorbificans	2
1579	Serum agglutination	Heilongjiang	2016	Poultry	880	86	Agona	2
							Blegdam	3
							Enteritidis	46
							Others	2
							Indiana	33
1587	Serum agglutination	Shanghai	2016	Poultry	500	28	Enteritidis	16
							Indiana	5
							Typhimurium	3
							Others	4
1598	NA	Zhejiang	2014	Human	317	44	Typhimurium	5
							Enteritidis	4
							Derby	2
							London	2
							Paratyphi B	2
							Aberdeen	1
							Albany	1
							Panama	1
							Others	26
1620	Serum agglutination	Sichuan	2012-2015	Human	86971	43	Typhimurium	5
							Derby	8
							Bredeney	1
							Bonn	1
							Braenderup	1
							Infantis	1
							Bareilly	4
							Montevideo	2
							Rissen	2
							Newport	1
							Kentucky	1
							Bardo	1
							Enteritidis	3
							Sinstorf	2
							London	2
							Muenster	1
							Meleagridis	1
							Senftenberg	5
							Aberdeen	1
1628	Serum agglutination	Guangdong	2015	Human	822	25	Typhimurium	7
							Enteritidis	4
							Stanley	3
							Typhimurium	7
							Enteritidis	3
							Stanley	1
1634	Serum agglutination	Zhejiang	2014-2016	Human	6262	54	Enteritidis	1
							Saintpaul	2
							Typhi	2
							London	3
							Thompson	3
							Derby	5
							Others	17
							Typhimurium	21
1639	Serum agglutination	Jiangsu	2016	Human	71694	118	Newport	2
							Litchfield	2
							Kottbus	1
							Others	24
							Agona	11
							Typhimurium	4
							Derby	3
							Others	7
							Rissen	2
							Montevideo	3
							Lomita	4
							Thompson	1
							Papuana	1
							Others	10
							Senftenberg	12
							Others	8
							Enteritidis	7
							Dublin	6
							Anatum	1
							London	2
							Okefoko	1
							Others	6
1648	Serum agglutination	Shanghai	2010-2014	Human	2119	374	Enteritidis	106
							Typhimurium	110
							Others	158
1664	Serum agglutination	Jilin	2016-2019	Swine	450	17	Typhimurium	8
							Choleraesuis	7
							Derby	2
1671	NA	Beijing	2015	Human	403	25	Enteritidis	10
							Stanley	6
							Typhimurium	5
							London	1

							Infantis	1
							Braenderup	1
							Litchfield	1
1691	NA	Fujian	2000-2013	Swine	614	87	Enteritidis	2
							Okefoko	2
							Senftenberg	2
							Newport	3
							London	4
							Anatum	5
							Agona	8
							Typhimurium	10
							Others	21
							Derby	30
		Fujian	2000-2013	Ruminants	800	90	Enteritidis	1
							Senftenberg	1
							Okefoko	2
							Anatum	3
							Agona	4
							Typhimurium	4
							Newport	8
							Thompson	8
							Weltevreden	11
							Others	20
							Derby	28
		Fujian	2000-2013	Poultry	663	79	Derby	9
							Typhimurium	17
							Agona	19
							Enteritidis	22
							Weltevreden	2
							Newport	1
							Anatum	1
							Litchfield	4
							London	2
							Senftenberg	1
							Okefoko	0
							Others	1
		Fujian	2000-2013	Food	6443	46	Derby	9
							Typhimurium	3
							Agona	1
							Enteritidis	4
							Weltevreden	2
							Newport	2
							Anatum	1
							Litchfield	2
							London	1
							Senftenberg	2
							Okefoko	0
							Thompson	1
							Others	18
1696	Serum agglutination	Beijing	2012-2016	Human	1948	122	Derby	8
							Agona	3
							Braenderup	3
							London	3
							Stanley	8
							Others	3
							Senftenberg	18
							Typhimurium	18
							Enteritidis	58
1704	AIS	Guangdong	2014-2015	Human	555	61	Typhimurium	35
							Typhi	8
							Paratyphi A	6
							Choleraesuis	3
							Others	9
1706	NA	Zhejiang	2015-2016	Human	802	17	Typhimurium	3
							Typhimurium	8
							Enteritidis	6
1709	Serum agglutination	Zhejiang	2014	Human	4095	46	Typhimurium	23
							Derby	4
							Enteritidis	3
							Gallinarum	2
							Muenster	2
							Others	12
1761	Serum agglutination	Henan	2016	Swine	840	67	Derby	32
							Typhimurium	31
							Give	2
							Reading	1
							Tumodi	1
1765	Serum agglutination	Jiangsu	2015	Human	756	32	Enteritidis	9
							Typhimurium	8
							London	4
							Agona	3
							Thompson	2
							Others	6

1767	Serum agglutination	Guangdong	2014-2015	Human	412	50	Enteritidis	12
							Senftenberg	5
							Stanley	5
							Typhimurium	4
							Litchfield	4
							Essen	1
							Papuana	1
							Derby	1
							Fillmore	1
							Ruzizi	1
							London	1
							Manchester	1
							Uganda	1
							Others	12
1777	Serum agglutination	Henan	2016	Poultry	2915	1031	Enteritidis	739
							Indiana	214
							Southampton	21
							Typhimurium	19
							Corvallis	8
							Kentucky	7
							Thompson	7
							Others	4
							Hadar	2
							Schwarzengrund	2
							Infantis	2
							Others	2
							Panama	1
							Bovismorbificans	1
							Blockley	1
							Gallen	1
1791	Serum agglutination	Sichuan	2016	Human	15613	6	Infantis	1
							Thompson	1
							Manhattan	1
							Derby	1
							Agona	1
							Others	1
1803	Serum agglutination	Beijing	2014-2016	Human	1400	56	Enteritidis	1
							Derby	7
							Dublin	18
							Typhimurium	30
1826	Serum agglutination	Henan	2015-2016	Human	5720	221	Agona	2
							Indiana	3
							Thompson	13
							Enteritidis	63
							Others	67
							Typhimurium	73
1849	Serum agglutination	Sichuan	2015-2016	Human	494	59	Bovismorbificans	1
							Duesseldorf	1
							Escanaba	1
							Infantis	1
							Kottbus	1
							Oranienburg	1
							Rissen	1
							Blegdam	2
							Enteritidis	2
							London	2
							Thompson	2
							Agona	3
							Derby	4
							Typhimurium	37
1920	Serum agglutination	Liaoning	2013-2015	Human	1288	439	Stanley	103
							Typhimurium	92
							Derby	17
							Agama	8
							Agona	7
							Indiana	3
							Cremieu	63
							Allerton	5
							Albany	1
							Litchfield	7
							Newport	1
							Fillmore	10
							London	1
							Meleagridis	5
							Senftenberg	4
							Rissen	2
							Thompson	5
							Potsdam	6
							Virchow	2
							Galiema	11
							Choleraesuis	9
							Isangi	1
							Enteritidis	59
							Typhi	5

							Blegdam	3
							Javiana	2
							Gallen	1
							Poona	1
							Pomona	1
							Kingabwa	4
1938	Serum agglutination	Jiangsu	2013-2014	Human	609	14	Typhimurium	6
							Derby	2
							Others	1
							Lomita	1
							Thompson	1
							Enteritidis	1
							Others	1
							Others	1
1946	NA	Xinjiang	2013-2015	Human	1363	15	Paratyphi A	1
							Paratyphi C	1
							Others	3
							Others	10
1956	NA	Shanghai	2015	Food	1201	11	Derby	4
							Corvallis	2
							Typhimurium	1
							Stanley	1
							Agona	1
							London	1
							Muenster	1
1986	Serum agglutination	Guangdong	2014-2015	Human	2456	283	Typhimurium	193
							Enteritidis	33
							Stanley	11
							Derby	10
							Corvallis	6
							Bovismorbificans	3
							Agona	3
							Goldcoast	2
							Paratyphi B	2
							Derby	2
							Typhi	2
							Others	2
							Javiana	2
							Singapore	2
							London	1
							Saintpaul	1
							Weltevreden	1
							Wandsworth	1
							Indiana	1
							Rissen	1
							Duesseldorf	1
							Poona	1
							Infantis	1
							Essen	1
1987	Serum agglutination	Zhejiang	2014	Human	3927	46	Typhimurium	23
							Derby	4
							Enteritidis	3
							Gallinarum	2
							Muenster	2
							Others	12
1997	Serum agglutination	Guangdong	2014-2015	Human	1319	76	Typhimurium	21
							Enteritidis	16
							Agona	3
							Chester	1
							Derby	1
							Paratyphi A	1
							London	1
							Senftenberg	1
							Typhi	1
							Stanley	1
							Thompson	3
							Infantis	1
							Others	25
2000	Serum agglutination	Jiangsu	2015	Poultry	88	18	Typhimurium	4
							Indiana	4
							Enteritidis	8
							Senftenberg	2
		Jiangsu	2015	Ruminants	80	9	Typhimurium	3
							Indiana	2
							Enteritidis	2
							Anatum	2
		Jiangsu	2015	Swine	65	4	Typhimurium	1
							Thompson	1
							Anatum	1
							London	1
		Jiangsu	2015	Cold-blooded animals	152	1	Others	1

2002	Serum agglutination	Shanghai	2010-2014	Human	8353	285	Meleagridis	5
							Stanley	5
							London	7
							Derby	8
							Agona	10
							Typhimurium	62
							Others	80
							Enteritidis	108
2005	Serum agglutination	Henan	2010-2014	Human	1762	82	Derby	2
							Saintpaul	2
							Paratyphi B	3
							Stanley	7
							Enteritidis	12
							Others	21
							Typhimurium	35
2013	NA	Guangdong	2013-2014	Human	1719	76	Enteritidis	25
							Typhimurium	17
							Senftenberg	7
							Weltevreden	6
							London	5
							Agona	5
							Derby	3
							Stanley	3
							Ruzizi	1
							Regent	1
							Others	3
2014	NA	Zhejiang	2011-2014	Human	14658	447	Paratyphi A	152
							Enteritidis	50
							Derby	40
							Anatum	26
							Agona	22
							Choleraesuis	17
							Potsdam	15
							Typhimurium	15
							Typhi	14
							Senftenberg	10
							Stanley	9
							Thompson	9
							Saintpaul	8
							Blegdam	7
							London	6
							Newport	5
							Zongo	5
							Sendai	3
							Newlands	2
							Paratyphi B	2
							Others	30
2021	Serum agglutination	Guangdong	2013-2015	Human	3373	271	Typhimurium	86
							Enteritidis	50
							London	18
							Stanley	10
							Weltevreden	10
							Derby	7
							Agona	5
							Infantis	3
							Braenderup	3
							Chester	2
							Fillmore	2
							Paratyphi B	1
							Others	74
2029	Serum agglutination	Jiangsu	2014	Human	915	55	Enteritidis	11
							Typhimurium	11
							London	4
							Lagos	4
							Newport	4
							Rissen	3
							Others	18
2061	Serum agglutination	Heilongjiang	2012	Poultry	1590	339	Agona	5
							Blegdam	5
							Enteritidis	188
							Gallinarum	1
							Paratyphi A	1
							Rissen	2
							Others	5
							Senftenberg	1
							Indiana	131
2065	Serum agglutination	Jiangsu	2014-2015	Human	860	108	Typhimurium	46
							Stanley	8
							Derby	7
							Newport	7
							Enteritidis	6
							London	4
							Virchow	4
							Poona	4

							Paratyphi B	3
							Wandsworth	2
							Redba	2
							Saintpaul	2
							Senftenberg	1
							Agona	1
							Virchow	1
							Bovismorbificans	1
							Hindmarsh	1
							Rissen	1
							Corvallis	1
							Goldcoast	1
							Tshiongwe	1
							Kottbus	1
							Give	1
							Panama	1
							Others	1
2086	Serum agglutination	Shanghai	2014	Human	380	10	Enteritidis	4
							Typhimurium	3
							Others	3
2091	Serum agglutination	Jiangsu	2012-2014	Human	2678	87	Blegdam	6
							Enteritidis	6
							Typhimurium	5
							Arizonae	3
							Oranienburg	2
							Tsevie	2
							Newlands	1
							Derby	1
							Agona	1
							Braenderup	1
							Eko	1
							Others	58
2097	Serum agglutination	Beijing	2014	Human	308	19	Choleraesuis	4
							Enteritidis	2
							Typhimurium	4
							Derby	1
							Blegdam	4
							Paratyphi B	1
							Thompson	1
							Essen	1
							Kiel	1
2115	Serum agglutination	Zhejiang	2014	Human	499	5	Agona	4
							Typhimurium	1
2133	Serum agglutination	Shanghai	2011-2013	Human	697	147	Enteritidis	64
							Typhimurium	29
							Derby	2
							Agona	8
							Senftenberg	4
							Lomita	1
							Wandsworth	3
							Others	36
2134	Serum agglutination	Heilongjiang	2010-2015	Human	1754	26	Typhimurium	26
2138	Serum agglutination	Guangdong	2011-2013	Human	2893	227	Typhimurium	122
							Enteritidis	29
							Stanley	13
							Others	63
2149	Serum agglutination	Zhejiang	2013-2014	Human	426	26	Typhimurium	9
							Infantis	4
							Rissen	1
							Bardo	2
							Pakistan	1
							Bovismorbificans	1
							Enteritidis	3
							Meleagridis	1
							London	4
2165	NA	Guangdong	2010-2014	Human	6707	290	Typhimurium	81
							Enteritidis	72
							Others	137
2173	Serum agglutination	Shanghai	2011	Human	1951	99	Enteritidis	27
							Typhimurium	26
							Senftenberg	9
							London	7
							Derby	6
							Irumu	3
							Thompson	3
							Infantis	2
							Agona	2
							Braenderup	2
							Virchow	2
							Rissen	2
							Istanbul	1
							Escanaba	1
							Remo	1
							Aberdeen	1

							Panama	1
							Others	1
							Muenchen	1
							Sandiego	1
		Shanghai	2012	Human	2537	120	Enteritidis	39
							Typhimurium	31
							Senftenberg	8
							London	6
							Aberdeen	6
							Derby	5
							Wandsworth	4
							Irumu	3
							Agona	3
							Stanley	2
							Braenderup	2
							Putten	2
							Thompson	2
							Sandiego	1
							Infantis	1
							Weltevreden	1
							Litchfield	1
							Galiema	1
							Sinstorf	1
							Anatum	1
		Shanghai	2013	Human	1658	80	Enteritidis	25
							Typhimurium	21
							Irumu	6
							London	4
							Others	9
							Aberdeen	2
							Rissen	2
							Senftenberg	2
							Derby	1
							Galiema	1
							Ruzizi	1
							Loanda	1
							Mbandaka	1
							Wandsworth	1
							Southampton	1
							Idikan	1
							Infantis	1
		Shanghai	2014	Human	1480	61	Typhimurium	20
							Enteritidis	17
							Derby	3
							London	3
							Newport	3
							Give	2
							Litchfield	2
							Mbandaka	2
							Senftenberg	2
							Panama	1
							Bovismorbificans	1
							Corvallis	1
							Infantis	1
							Others	1
							Stanley	1
							Thompson	1
2183	PCR	Guangxi	2016	Swine	300	98	Derby	33
							Typhimurium	20
							Thompson	12
							Agona	12
							Enteritidis	10
							Tsevie	5
							Lagos	4
							Lomita	2
2190	Serum agglutination	Gansu	2013-2015	Human	1617	9	Teshie	2
							Enteritidis	2
							Montevideo	1
							Oranienburg	1
							Stanleyville	1
							Thompson	1
							Lagos	1
2191	Serum agglutination	Sichuan	2012-2014	Human	982	35	Typhimurium	12
							Paratyphi B	2
							Derby	2
							Agona	1
							Lagos	1
							Others	2
							Escanaba	1
							Virchow	1
							Infantis	1
							Enteritidis	3
							Blegdam	2
							Anatum	2

							London	1
							Paratyphi A	1
							Muenchen	1
							Bovismorbificans	1
							Kouka	1
2201	Serum agglutination	Fujian	2009--2014	Human	3287	156	Bovismorbificans	1
							Chester	1
							Dublin	1
							Fufu	1
							Meleagridis	1
							Montevideo	1
							Muenchen	1
							Newlands	1
							Newport	1
							Paratyphi A	1
							Saintpaul	1
							Thompson	1
							Choleraesuis	2
							London	2
							Gallinarum	3
							Litchfield	3
							Derby	4
							Agona	5
							Paratyphi C	5
							Paratyphi B	7
							Stanley	10
							Enteritidis	19
							Typhimurium	84
2203	Serum agglutination	Inner Mongolia	2014	Human	771	43	Typhimurium	26
							Others	17
2224	Serum agglutination	Shanghai	2015	Food	780	11	Derby	4
							Corvallis	2
							Typhimurium	1
							Stanley	1
							Agona	1
							London	1
							Muenster	1
2254	Serum agglutination	Guangdong	2014-2015	Human	2465	283	Typhimurium	194
							Enteritidis	33
							Stanley	11
							Derby	10
							Corvallis	6
							Bovismorbificans	3
							Agona	3
							Goldcoast	2
							Paratyphi B	2
							Derby	2
							Typhi	2
							Javiana	2
							Singapore	1
							London	1
							Saintpaul	1
							Weltevreden	1
							Wandsworth	1
							Indiana	1
							Rissen	1
							Duesseldorf	1
							Pomona	1
							Infantis	1
							Essen	1
							Others	2
2265	Serum agglutination	Guangdong	2014-2015	Human	1319	76	Typhimurium	21
							Enteritidis	16
							Agona	3
							Chester	1
							Derby	1
							Paratyphi A	1
							London	1
							Senftenberg	1
							Typhi	1
							Stanley	3
							Thompson	1
							Infantis	2
							Others	24
2268	Serum agglutination	Jiangsu	2015	Poultry	88	18	Typhimurium	4
							Indiana	4
							Enteritidis	8
							Senftenberg	2
		Jiangsu	2015	Ruminants	80	9	Typhimurium	3
							Indiana	2
							Enteritidis	2
							Anatum	2
		Jiangsu	2015	Swine	65	4	Typhimurium	1

							Thompson	1
							Anatum	1
							London	1
		Jiangsu	2015	Cold-bloo	152	1	Enteritidis	1
2270	Serum agglutination	Shanghai	2010-2014	Human	8535	285	Meleagridis	5
							Stanley	5
							London	7
							Agona	8
							Derby	8
							Agona	10
							Senftenberg	11
							Typhimurium	62
							Others	61
							Enteritidis	108
2281	Serum agglutination	Guangdong	2013-2014	Human	1719	76	Enteritidis	25
							Typhimurium	17
							Senftenberg	7
							Weltevreden	6
							London	5
							Derby	5
							Stanley	3
							Others	3
							Ruzizi	1
							Regent	1
							Others	3
2282	Serum agglutination	Zhejiang	2011-2014	Human	14658	447	Paratyphi A	152
							Enteritidis	50
							Derby	40
							Anatum	26
							Agona	22
							Choleraesuis	17
							Potsdam	15
							Typhimurium	15
							Typhi	14
							Senftenberg	10
							Stanley	9
							Thompson	9
							Saintpaul	8
							Blegdam	7
							London	6
							Newport	5
							Zongo	5
							Sendai	3
							Newlands	2
							Paratyphi B	2
							Others	30
2289	Serum agglutination	Guangdong	2013-2015	Human	3373	271	Typhimurium	86
							Enteritidis	50
							London	18
							Stanley	10
							Weltevreden	10
							Derby	7
							Agona	5
							Infantis	3
							Braenderup	3
							Chester	2
							Fillmore	2
							Paratyphi B	1
							Others	74
2329	Serum agglutination	Heilongjiang	2012	Poultry	3766	339	Agona	5
							Blegdam	5
							Enteritidis	188
							Gallinarum	1
							Paratyphi A	1
							Rissen	2
							Others	5
							Senftenberg	1
							Indiana	131
2333	Serum agglutination	Zhejiang	2014-2015	Human	860	108	Typhimurium	46
							Stanley	8
							Derby	7
							Newport	7
							Enteritidis	6
							London	4
							Virchow	4
							Poona	4
							Paratyphi B	3
							Wandsworth	2
							Redba	2
							Saintpaul	2
							Senftenberg	2
							Agona	1
							Bovismorbificans	1
							Hindmarsh	1

							Rissen	1
							Corvallis	1
							Goldcoast	1
							Tshiongwe	1
							Kottbus	1
							Give	1
							Panama	1
							Others	1
2354	Serum agglutination	Shanghai	2014	Human	380	10	Enteritidis	4
							Typhimurium	3
							Others	3
2359	Serum agglutination	Jiangsu	2012-2014	Human	2678	87	Blegdam	6
							Enteritidis	6
							Typhimurium	5
							Arizonae	3
							Oranienburg	2
							Tsevie	2
							Newlands	1
							Derby	1
							Agona	1
							Braenderup	1
							Eko	1
							Others	58
2365	Serum agglutination	Beijing	2014	Human	308	19	Choleraesuis	4
							Enteritidis	2
							Typhimurium	4
							Derby	1
							Blegdam	4
							Paratyphi B	1
							Thompson	1
							Essen	1
							Kilva	1
2402	Serum agglutination	Heilongjiang	2010-2015	Human	1754	26	Typhimurium	26
2406	Serum agglutination	Guangdong	2011-2013	Human	2893	227	Typhimurium	122
							Enteritidis	29
							Stanley	13
							Others	63
2408	Serum agglutination	Shanghai	2014	Human	1218	47	Typhimurium	12
							Enteritidis	11
							Derby	4
							London	4
							Others	16
2417	Serum agglutination	Zhejiang	2013-2014	Human	426	26	Typhimurium	9
							Infantis	4
							Rissen	1
							Bardo	2
							Pakistan	1
							Bovismorbificans	1
							Enteritidis	3
							Meleagridis	1
							London	4
2423	Serum agglutination	Guangdong	2012-2015	Human	7047	459	Typhimurium	176
							Paratyphi B	3
							Enteritidis	60
							Typhi	1
							Others	219
2441	Serum agglutination	Shanghai	2011-2014	Human	7626	360	Typhimurium	98
							Derby	15
							Agona	5
							Irumu	12
							Thompson	6
							Infantis	5
							Newport	4
							Litchfield	3
							Corvallis	1
							Enteritidis	108
							Panama	2
							London	20
							Give	2
							Senftenberg	21
							Aberdeen	9
							Havana	2
							Putten	2
							Putten	1
							Wandsworth	5
							Others	39
2445	Serum agglutination	Fujian	1993-2015	Human	15074	474	Paratyphi B	7
							Stanley	20
							Saintpaul	3
							Reading	17
							Derby	83
							Agona	15
							Typhimurium	38
							Shubra	1

							Isangi	1
							Livingstone	1
							Braenderup	10
							Rissen	5
							Montevideo	2
							Oranienburg	1
							Thompson	12
							Singapore	11
							Escanaba	5
							Potsdam	2
							Virchow	5
							Infantis	4
							Bareilly	3
							Businga	1
							Mbandaka	12
							Newport	14
							Kottbus	6
							Blockley	15
							Litchfield	2
							Goldcoast	2
							Bovismorbificans	5
							Chailey	2
							Corvallis	3
							Albany	2
							Hadar	1
							Enteritidis	49
							Muenster	5
							Anatum	17
							Meleagridis	7
							London	27
							Weltevreden	32
							Senftenberg	7
							Taksony	2
							Poona	1
							Ramatgan	1
							Others	15
2454	Serum agglutination	Jiangsu	2015	Human	464	25	Lagos	11
							Dublin	6
							Saintpaul	2
							Stanley	1
							Infantis	1
							Muenchen	1
							Kottbus	3
2469	Serum agglutination	Fujian	2009-2014	Human	3287	156	Typhimurium	84
							London	22
							Enteritidis	19
							Stanley	10
							Agona	5
							Derby	4
							Litchfield	3
							Choleraesuis	2
							Dublin	1
							Meleagridis	1
							Montevideo	1
							Newlands	1
							Newport	1
							Saintpaul	1
							Thompson	1
2490	Serum agglutination	Zhejiang	2013-2014	Human	1698	5	Dublin	2
							Typhimurium	1
							Paratyphi B	1
							Others	1
2496	Serum agglutination	Xinjiang	2013-2014	Ruminant	535	30	Schwarzengrund	2
							Paratyphi B	1
							Typhimurium	1
							Mbandaka	3
							Braenderup	1
							Thompson	1
							Tennessee	1
							Hadar	9
							Enteritidis	3
							London	4
							Assinie	1
							Havana	3
2499	Serum agglutination	Shandong	2014	Swine	1000	188	Derby	80
							Typhimurium	56
							Thompson	34
							Agona	3
							Enteritidis	1
							Tsevie	2
							Lagos	1
							Oritamerin	1
							Lomita	2
							Others	8

2500	Serum agglutination	Guangdong	2010-2013	Human	3604	142	Typhimurium	36
							Enteritidis	34
							Others	72
2504	Serum agglutination	Shandong	2012-2013	Poultry	720	128	Enteritidis	1
							Kodek	2
							Others	1
							Dublin	3
							Thompson	9
							Gallinarum	16
							Enteritidis	23
							Indiana	73
2526	Serum agglutination	Guangdong	2013-2014	Human	582	24	Indiana	1
							Typhimurium	6
							Stanley	3
							Derby	1
							Braenderup	1
							London	1
							Virchow	1
							Enteritidis	9
							Dublin	1
2544	Serum agglutination	Shanghai	2012	Human	2423	69	Typhimurium	26
							Enteritidis	22
							Gallinarum	1
							Anatum	1
							Bovismorbificans	4
							Kottbus	1
							Senftenberg	1
							Aberdeen	2
							London	3
							Derby	5
							Thompson	1
							Larochelle	1
							Infantis	1
2560	Serum agglutination	Zhejiang	2010-2013	Human	10917	614	Enteritidis	334
							Typhimurium	280
2575	Serum agglutination	Guangdong	2010-2013	Human	1922	92	Brezany	1
							Derby	1
							Lagos	1
							Newlands	1
							Norwich	1
							Othmarschen	1
							Papuana	1
							Paratyphi A	1
							Saintpaul	1
							Thompson	1
							Virchow	1
							Others	1
							Fillmore	2
							London	2
							Rissen	2
							Sarajane	3
							Stanley	7
							Enteritidis	29
							Typhimurium	35
2578	Serum agglutination	Beijing	2013-2014	Human	605	35	Typhimurium	14
							Derby	7
							Enteritidis	3
							Dublin	11
2595	Serum agglutination	Beijing	2014	Human	330	20	Derby	3
							Dublin	9
							Typhimurium	8
2596	Serum agglutination	Beijing	2013-2014	Human	542	34	Enteritidis	19
							Agona	4
							Braenderup	2
							Others	9
2603	Serum agglutination	Sichuan	2009-2014	Poultry	5445	154	Paratyphi B	3
							Typhimurium	5
							Derby	9
							Others	19
							Gallinarum	21
							Enteritidis	97
2605	Serum agglutination	Zhejiang	2014	Food	6812	208	Enteritidis	26
							Derby	30
							Anatum	6
							Agona	18
							Choleraesuis	22
							Potsdam	12
							Typhimurium	15
							Senftenberg	9
							Stanley	10
							Thompson	3
							Saintpaul	12
							Blegdam	8
							London	13

							Newport	8
							Others	16
2627	Serum agglutination	Guangxi	2012-2014	Ruminants	192	26	Derby	12
							London	2
							Typhimurium	2
							Indiana	2
							Infantis	2
							Agona	2
							Anatum	2
							Enteritidis	2
2632	Serum agglutination	Guangdong	2013	Human	9847	329	Stanley	39
							Saintpaul	1
							Derby	16
							Agona	7
							Typhimurium	72
							Agama	9
							Indiana	1
							Indiana	2
							Choleraesuis	1
							Infantis	1
							Thompson	4
							Singapore	2
							Potsdam	5
							Nigeria	1
							Virchow	2
							Braenderup	2
							Kastrup	1
							Rissen	13
							Othmarschen	1
							Galiema	1
							Bareilly	2
							Isangi	1
							Larose	1
							Newport	1
							Kottbus	2
							Cremieu	1
							Allerton	1
							Corvallis	2
							Albany	3
							Litchfield	3
							Fillmore	1
							Typhi	1
							Enteritidis	87
							Blegdam	1
							Istanbul	1
							Tarshyne	1
							Javiana	1
							Javiana	1
							Galil	1
							Meleagridis	2
							Weltevreden	7
							London	7
							Senftenberg	1
							Poona	2
							Amoutive	1
							Pomona	2
							Kingabwa	1
							Others	13
2652	Serum agglutination	Shandong	2012	Poultry	1267	298	Indiana	143
							Enteritidis	138
							Thompson	5
							Aba	2
							Nigeria	1
							Chailey	1
							Agona	1
							Southampton	1
							Others	6
2653	Serum agglutination	Beijing	2011-2013	Human	1179	69	Senftenberg	14
							Enteritidis	14
							Typhimurium	11
							Agona	5
							Indiana	5
							Others	20
2665	Serum agglutination	Guangxi	2013-2015	Poultry	310	34	Paratyphi A	1
							Typhimurium	14
							Reading	1
							Newport	1
							Gallinarum	12
							Enteritidis	1
							Typhi	1
							Others	3
2684	Serum agglutination	Shandong	2014	Poultry	995	239	Enteritidis	42
							Indiana	33

							Agona	11
							Typhimurium	10
							Thompson	85
							Albany	1
							Derby	35
							Rissen	2
							Others	20
2689	Serum agglutination	Henan	2012	Poultry	802	234	Typhimurium	17
							Indiana	55
							Thompson	5
							Enteritidis	76
							Indiana	53
							Thompson	1
							Albany	10
							Agona	1
							Africana	1
							Mbandaka	7
							Infantis	7
							Hadar	1
2706	Serum agglutination	Beijing	2013-2014	Human	366	18	Enteritidis	6
							Senftenberg	6
							Derby	3
							Typhimurium	1
							Braenderup	1
							Saintpaul	1
2723	Serum agglutination	Guangdong	2012-2013	Human	863	80	Typhimurium	27
							Enteritidis	16
							Others	37
2749	Serum agglutination	Shanghai	2010	Human	824	75	Enteritidis	16
							Typhimurium	12
							Derby	4
							Meleagridis	4
							Oranienburg	3
							Others	36
		Shanghai	2011	Human	1204	82	Enteritidis	35
							Typhimurium	21
							Derby	4
							London	3
							Stanley	3
							Others	16
		Shanghai	2012	Human	551	28	Enteritidis	14
							Typhimurium	5
							London	1
							Corvallis	1
							Rissen	1
							Others	6
2753	Serum agglutination	Shanxi	2013-2014	Human	789	18	Enteritidis	8
							Typhimurium	5
							Others	5
2756	Serum agglutination	Zhejiang	2008-2015	Human	9256	369	Newport	2
							Paratyphi B	2
							London	3
							Blegdam	5
							Saintpaul	6
							Thompson	7
							Stanley	8
							Senftenberg	10
							Typhimurium	11
							Choleraesuis	12
							Potsdam	12
							Typhi	14
							Agona	16
							Anatum	20
							Others	26
							Derby	28
							Enteritidis	35
							Paratyphi A	152
2773	Serum agglutination	Guangdong	2009-2014	Human	4395	546	Javiana	2
							Kottbus	2
							Virchow	2
							Adelaide	3
							Corvallis	3
							Albany	4
							Litchfield	4
							Thompson	5
							Agona	6
							Infantis	7
							Weltevreden	7
							Saintpaul	9
							Rissen	13
							London	14
							Derby	17
							Newport	19
							Others	21

							Stanley	56
							Enteritidis	74
							Typhimurium	278
2788	Serum agglutination	Guangdong	2012-2013	Food	576	105	Derby	27
							Typhimurium	13
							Stanley	5
							Agona	4
							Tafo	1
							Shubra	1
							Ituri	1
							Heidelberg	2
							Rissen	5
							Infantis	2
							Fillmore	1
							Corvallis	1
							Virchow	2
							Rissen	1
							Litchfield	2
							Newport	1
							Hartford	1
							Chailey	1
							Enteritidis	2
							Weltevreden	9
							London	3
							Newlands	3
							Senftenberg	1
							Vejile	1
							Anatum	1
							Give	1
							Others	13
2795	Serum agglutination	Hunan	2010	Poultry	152	36	Agona	4
							Albany	4
							Albert	1
							Bluka	2
							Bousso	2
							Brancaster	1
							Corvallis	2
							Derby	3
							Kalina	1
							Kentucky	3
							Menden	1
							Newport	1
							Paratyphi A	1
							Rissen	1
							Shubra	1
							Thompson	1
							Typhimurium	3
							Vejile	3
							Others	1
		Hunan	2010	Food	540	58	agona	1
							alamo	1
							albany	1
							albert	0
							Augustenborg	2
							Blukwa	0
							bonn	4
							bousso	1
							brancaster	0
							corvallis	0
							derby	30
							fillmore	1
							kalina	1
							kentucky	0
							menden	0
							Menston	1
							montevideo	1
							newport	0
							paratyphi A	0
							Rechovot	2
							rissen	0
							shubra	0
							stanley	2
							thompson	3
							typhimurium	2
							Typhisuis	1
							Vejile	2
							Wilmington	1
							Others	1
2803	Serum agglutination	Sichuan	2007-2012	Food	1081	19	Shubra	1
							Enteritidis	1
							Indiana	1
							Others	1
							Stanley	1
							Virchow	1

							Agona	2
							Newport	2
							Typhimurium	4
							Derby	5
2815	Serum agglutination	Hunan	2009-2011	Human	767	35	Dabou	1
							Infantis	1
							London	1
							Moualine	1
							Weltevreden	1
							Derby	2
							Senftenberg	2
							Stanley	3
							Enteritidis	10
							Typhimurium	13
2822	Serum agglutination	Inner Mongolia	2006-2011	Ruminants	460	38	Agona	1
							Choleraesuis	1
							Derby	1
							Paratyphi A	1
							Potsdam	1
							Senftenberg	1
							Thompson	1
							Aberdeen	2
							Paratyphi B	2
							Typhi	2
							Anatum	3
							Manhattan	3
							Newport	4
							Typhimurium	15
2834	Serum agglutination	Jiangxi	2009-2014	Food	3450	136	Aberdeen	1
							Bardo	1
							Braenderup	1
							Choleraesuis	1
							Irumu	1
							Pakistan	1
							Stanley	1
							Anatum	2
							Chester	2
							Reading	2
							London	3
							Newport	3
							Paratyphi A	4
							Agona	11
							Enteritidis	8
							Typhimurium	11
							Meleagridis	16
							Derby	67
2837	Serum agglutination	Shandong	2006-2007	Poultry	992	178	Enteritidis	178
2863	Serum agglutination	Guangdong	2013	Human	1150	72	Typhimurium	22
							Enteritidis	15
							Stanley	6
							Agama	4
							Rissen	4
							Pomona	3
							Derby	2
							Agona	2
							Weltevreden	2
							Saintpaul	1
							Newport	1
							Bareilly	1
							Typhisuis	1
							Infantis	1
							Bristol	1
							Nigeria	1
							Colindale	1
							Thompson	1
							Dublin	1
							Albany	1
							Agona	1
2889	Serum agglutination	Heilongjiang	2012	Swine	600	60	Choleraesuis	25
							Others	33
							Typhimurium	2
2900	Serum agglutination	Guangxi	2009-2012	Feed	468	7	Essen	1
							Choleraesuis	1
							Gallinarum	1
							Nagoya	1
							Others	1
							Give	1
							Agona	1
2906	Serum agglutination	Shanghai	2012	Poultry	320	70	Enteritidis	33
							Indiana	12
							Typhimurium	6
							Derby	6
							Corvallis	5

							Hadar	2
							Thompson	2
							Havana	1
							Meleagridis	1
							Rissen	1
							Braenderup	1
2912	Serum agglutination	Shanghai	2013	Human	583	19	Enteritidis	10
							Typhimurium	6
							Derby	1
							Agona	1
							Singapore	1
2924	Serum agglutination	Guangdong	2010-2012	Human	1095	45	Stanley	3
							Enteritidis	6
							Others	15
							Typhimurium	21
2946	Serum agglutination	Shandong	2012	Poultry	2496	497	Enteritidis	170
							Indiana	210
							Thompson	47
							Agona	2
							Others	19
							Aba	3
							Chailey	1
							Nigeria	1
							Southampton	1
							Malmore	1
							Clerkenwell	7
							Assinie	2
							Daula	2
							Clerkenwell	1
							Lamberhurst	1
							London	1
							Lindenberg	2
							Blegdam	1
							Rostork	1
							Konstanz	1
							Cocody	7
							Tounouma	4
							Augustenborg	3
							Akanji	1
							Irumu	1
							Sarajane	1
							Marmande	1
							Norton	1
							Papuana	1
							Bonariensis	1
							Neftenbach	1
							Preston	1
2964	Serum agglutination	Guangdong	2010-2013	Human	6417	460	Albany	5
							Agona	7
							London	8
							Newport	8
							Derby	9
							Thompson	10
							Rissen	16
							Enteritidis	40
							Stanley	62
							Others	83
							Typhimurium	212
2965	Serum agglutination	Guangdong	2007-2009	Human	760	53	Typhimurium	21
							Enteritidis	11
							Stanley	5
							Thompson	2
							Agona	2
							Paratyphi B	2
							London	2
							Stanleyville	2
							Hadar	1
							Indiana	1
							Anatum	1
							London	1
							Typhimurium	1
							Others	1
2993	Serum agglutination	Hebei	2013	Poultry	331	89	Enteritidis	35
							Apeyeme	1
							Enteritidis	10
							Fillmore	2
							Gallinarum	1
							Indiana	15
							Kentucky	5
							Paratyphi A	1
							Redba	2
							Rissen	3
							Senftenberg	14
2994	Serum agglutination	Guangdong	2009-2013	Human	6920	344	Typhimurium	1

							Corvallis	1
							Lezennes	1
							Litchfield	1
							Seegenfeld	1
							Weltevreden	2
							Newport	3
							Others	4
							Singapore	4
							Chester	5
							Infantis	5
							London	5
							Muenchen	6
							Virchow	6
							Dublin	8
							Derby	9
							Rissen	12
							Thompson	13
							Enteritidis	29
							Stanley	43
							Typhimurium	185
3006	Serum agglutination	Fujian	2010-2012	Food	477	12	Enteritidis	1
							Newport	1
							Typhimurium	1
							Ughelli	1
							Derby	2
							Others	6
3022	Serum agglutination	Guangxi	2009-2012	Food	701	31	Derby	9
							Tshiongwe	3
							Agona	1
							Newlands	1
							Eastglam	1
							Give	1
							Lindenberg	1
							Others	14
3023	Serum agglutination	Sichuan	2007-2009	Human	1008	52	Enteritidis	15
							Typhimurium	12
							Typhi	1
							Derby	8
							Blegdam	7
							Ughelli	4
							Kentucky	2
							Agona	3
3037	Serum agglutination	Henan	2012	Food	344	6	Enteritidis	2
							Agona	2
							Indiana	1
							Derby	1
3488	Serum agglutination	Guangdong	2010	Human	1027	20	Enteritidis	8
							Typhimurium	6
							Typhi	1
							Bonn	1
							Derby	1
							Derby	1
							Stanley	1
							Saintpaul	1
3538	Serum agglutination	Jiangsu	2010	Poultry	210	23	Indiana	22
							Others	1
		Jiangsu	2010	Poultry	204	71	Indiana	35
							Oranienburg	22
							Others	14
3542	Serum agglutination	Ningxia	2009-2011	Food	441	11	Enteritidis	10
							Senftenberg	1
3567	Serum agglutination	Henan	2011	Mouse	386	137	Typhimurium	137
3604	Serum agglutination	Sichuan	2010	Human	433	25	Typhimurium	16
							Others	9
3618	Serum agglutination	Jiangsu	2009-2010	Poultry	212	58	Paratyphi A	1
							Derby	16
							Agona	6
							Typhimurium	5
							Chester	2
							Irumu	1
							Braenderup	1
							Newport	2
							Pakistan	1
							Bardo	1
							Enteritidis	8
							London	2
							Meleagridis	11
							Anatum	1
		Jiangsu	2009-2010	Food	3225	97	Derby	1
							Paratyphi A	3
							Derby	58
							Agona	6
							Typhimurium	9
							Stanley	1

							Reading	2
							Chester	0
							Choleraesuis	1
							Irumu	1
							Braenderup	0
							Newport	1
							Pakistan	0
							Bardo	0
							Enteritidis	1
							London	1
							Meleagridis	6
							Anatum	1
							Aberdeen	1
							Typhi	3
							Thompson	1
3633	Serum agglutination	Hebei	2010	Human	117288	93	Dublin	1
							Paratyphi B	3
							Kingston	9
							Limete	3
							Derby	6
							Budapest	1
							Reading	1
							Montevideo	2
							Namibia	6
							Braenderup	9
							Edmonton	3
							Others	4
							London	2
							Meleagridis	1
							Senftenberg	10
							Assinie	3
							Rideau	11
							Coquilhatville	3
							Give	3
							Missouri	1
							Others	11
3649	Serum agglutination	Shanghai	2006-2010	Human	4105	181	Enteritidis	60
							Typhimurium	52
							London	19
							Choleraesuis	12
							Aberdeen	8
							Others	30
3651	Serum agglutination	Jiangsu	2011	Food	891	67	Indiana	38
							Enteritidis	17
							Anatum	6
							Typhimurium	2
							Albany	1
							Others	3
3664	Serum agglutination	Shanghai	2009-2010	Human	4170	206	Anatum	1
							Muenster	1
							Stanley	1
							Mendoza	2
							Potsdam	2
							Wandsworth	2
							Agona	3
							Derby	3
							Aberdeen	4
							Senftenberg	4
							Saintpaul	5
							Newport	6
							London	7
							Infantis	8
							Kottbus	9
							Choleraesuis	14
							Thompson	14
							Typhimurium	59
							Enteritidis	61
		Shanghai	2009-2010	Food	632	69	Enteritidis	13
							Typhimurium	14
							London	4
							Thompson	2
							Derby	8
							Agona	3
							Infantis	1
							Anatum	3
							Potsdam	1
							Saintpaul	2
							Newport	1
							Wandsworth	1
							Stanley	1
							Aberdeen	6
							Indiana	2
							Azteca	1
							Dublin	1

							Pomona	2
							Senftenberg	3
3673	Serum agglutination	Yunnan	2009	Human	588	2	Typhimurium	2
3675	NA	Henan	2009-2010	Human	1509	123	Typhimurium	47
							Enteritidis	22
							Agona	17
							Others	37
3679	Serum agglutination	Hunan	2009-2010	Human	10072	82	Typhimurium	48
							Others	34
3688	Serum agglutination	Shanghai	2010	Food	840	36	Typhimurium	22
							Dublin	5
							Reading	5
							Bardo	4
3727	Serum agglutination	Guangdong	2008-2009	Human	2472	44	Typhimurium	26
							Stanley	1
							Agona	1
							Paratyphi B	1
							Others	15
3732	Serum agglutination	Jiangsu	2006-2010	Food	533	33	Derby	10
							Reading	5
							Typhimurium	5
							Senftenberg	3
							Anatum	2
							Aberdeen	2
							Others	6
3734	Serum agglutination	Gansu	2007-2010	Human	739	9	Typhimurium	9
3744	Serum agglutination	Shanghai	2010	Human	1045	160	Typhimurium	65
							Enteritidis	40
							Agona	8
							Saintpaul	5
							Meleagridis	5
							Senftenberg	4
							Paratyphi B	4
							Infantis	4
							Irumu	3
							Aberdeen	3
							Thompson	2
							Stanley	2
							Derby	2
							Braenderup	2
							Anatum	1
							Potsdam	1
							Albany	1
							Bareilly	1
							Rissen	1
							Mbandaka	1
							Ordonez	1
							Others	4
3763	Serum agglutination	Henan	2006-2010	Human	2327	85	Agona	24
							Senftenberg	13
							Enteritidis	9
							Typhimurium	9
							Derby	7
							Schwarzengrund	5
							Tshiongwe	3
							Albany	2
							Bovismorbificans	2
							Blegdam	2
							Butantan	2
							Meleagridis	1
							Wandsworth	1
							Others	5
3773	Serum agglutination	Sichuan	2007-2010	Human	1130	14	Typhi	2
							Typhimurium	6
							Enteritidis	4
							Derby	1
							Agona	1
3774	Serum agglutination	Beijing	2010	Human	308	10	Bareilly	3
							Enteritidis	2
							Typhimurium	2
							Braenderup	2
							Others	1
3778	Serum agglutination	Guangdong	2008-2009	Human	65613	162	Typhimurium	74
							Enteritidis	42
							Agona	7
							Paratyphi B	11
							Others	2
							Derby	16
							Stanley	10
3787	Serum agglutination	Guangdong	2009-2010	Human	1665	72	Typhimurium	33
							Stanley	3
							Thompson	2
							Typhisuis	5
							Dublin	2

							Muenchen	2
							Newport	1
							Montevideo	1
							Saintpaul	1
							Agona	1
							Potsdam	1
							Others	20
3827	Serum agglutination	Hunan	2010	Human	11068	118	Typhi	31
							Derby	21
							Paratyphi A	18
							Senftenberg	17
							Enteritidis	15
							Papuana	6
							Bardo	5
							Lagos	4
							Paratyphi B	1
3837	Serum agglutination	Hubei	2006	Human	3746	221	Derby	60
							Typhimurium	49
							Agona	25
							Nessziona	15
							Saintpaul	11
							Assinie	10
							Enteritidis	8
							London	8
							Albany	4
							Sandiego	4
							Rissen	3
							Anatum	2
							Concord	2
							Escanaba	2
							Schleissheim	2
							Schwarzengrund	2
							Aberdeen	1
							Bovismorbificans	1
							Cannstatt	1
							Gabon	1
							Indiana	1
							Infantis	1
							Muenchen	1
							Oesterbro	1
							Senftenberg	1
							Singapore	1
							Stanley	1
							Tennessee	1
							Wandsworth	1
							Others	1
3842	Serum agglutination	Anhui	2010	Poultry	500	21	Saintpaul	1
							Tshiongwe	1
							Gallinarum	14
							Newlands	3
							Muenster	2
3856	Serum agglutination	Shaanxi	2007-2008	Poultry	515	260	Enteritidis	102
							Shubra	33
							Indiana	31
							Typhimurium	25
							Djugu	19
							Derby	13
							Virchow	7
							Othmarschen	6
							Others	7
							Tennessee	3
							Agona	2
							Infantis	2
							Kallo	2
							Galiema	2
							Thompson	1
							Braenderup	1
							Pakistan	1
							Litchfield	1
							Saintpaul	1
							Brancaster	1
3867	Serum agglutination	Jiangsu	2008-2010	Human	58382	258	Derby	52
							Agona	31
							Typhimurium	17
							Saintpaul	7
							Indiana	7
							Choleraesuis	24
							Tennessee	19
							Thompson	35
							Potsdam	5
							Kottbus	13
							Enteritidis	10
							Dublin	2
							London	5

							Anatum	8
							Senftenberg	12
							Others	11
3870	Serum agglutination	Guangdong	2008-2011	Food	2321	164	Others	39
							Derby	35
							Kentucky	11
							Weltevreden	10
							Indiana	10
							Typhimurium	8
							Senftenberg	8
							Chester	6
							Schwarzengrund	6
							Newlands	6
							Agona	5
							Saintpaul	5
							Anatum	4
							Tshiongwe	3
							Give	3
							Enteritidis	3
							Dublin	2
3878	Serum agglutination	Guangdong	2008-2010	Human	1260	52	Derby	6
							Agona	7
							Dublin	4
							Blegdam	3
							Enteritidis	32
3913	Serum agglutination	Anhui	2008	Human	480	13	Typhi	9
							Typhimurium	4
3919	Serum agglutination	Jiangxi	2008-2009	Human	1630	9	Derby	2
							Agona	1
							Typhimurium	2
							Newport	1
							Others	3
3935	Serum agglutination	Shanghai	2004-2009	Human	189068	134	Kottbus	1
							London	1
							Newport	1
							Paratyphi	1
							Indiana	2
							Lomita	2
							Reading	2
							Anatum	3
							Infantis	3
							Muenster	3
							Saintpaul	4
							Potsdam	5
							Thompson	6
							Aberdeen	8
							Agona	8
							Senftenberg	12
							Derby	21
							Typhimurium	25
							Enteritidis	26
3937	Serum agglutination	Heilongjiang	2005-2008	Food	1070	94	Lindenberg	1
							Newport	1
							Rissen	1
							Israel	2
							Others	2
							Dublin	3
							Saintpaul	3
							Gallinarum	4
							Typhimurium	5
							Choleraesuis	6
							Derby	19
							Agona	21
							Enteritidis	26
3942	Serum agglutination	Zhejiang	2008-2009	Swine	157	16	Typhimurium	7
							Derby	6
							Agona	1
							Choleraesuis	1
							Anatum	1
		Zhejiang	2008-2009	Poultry	135	12	Typhimurium	2
							Indiana	1
							Albany	1
							Shubra	1
							Tshiongwe	1
							Saintpaul	3
							Indiana	2
							Albany	1
		Zhejiang	2008-2009	Food	19	2	Typhimurium	1
							Derby	1
		Zhejiang	2008-2009	Ruminants	46	1	Agona	1
3956	Serum agglutination	Shandong	2003-2008	Food	1685	49	Massenya	1
							Newport	1
							Thompson	1

							Others	1
							Senftenberg	6
							Enteritidis	39
3964	Serum agglutination	Guangxi	2009	Food	1181	61	Saintpaul	1
							Enteritidis	3
							Derby	9
							Agona	2
							Kentucky	6
							Tshiongwe	4
							Bonn	1
							Senftenberg	3
							Virchow	1
							Newlands	3
							Typhimurium	6
							Indiana	1
							Zongo	1
							Brancaster	1
							Litchfield	1
							Glostrup	2
							Magherafelt	2
							Rissen	1
							Lagos	1
							Chester	1
							Paratyphi C	1
							Give	2
							Lindenberg	1
							Others	7
3972	Serum agglutination	Henan	2009-2010	Poultry	617	98	Enteritidis	61
							Pullorum	18
							Dublin	2
							Blegdam	1
							Others	16
3982	Serum agglutination	Sichuan	2009	Food	827	51	Typhimurium	15
							Derby	4
							Agona	2
							Stanley	1
							Choleraesuis	12
							Dublin	6
							Gallinarum	4
							Blegdam	3
							Enteritidis	2
							Anatum	2
3996	Serum agglutination	Jiangsu	2008	Food	757	44	Paratyphi C	4
							Derby	3
							Anatum	3
							Senftenberg	3
							Aberdeen	2
							Lomita	1
							Reading	1
							Others	27
4010	Serum agglutination	Henan	2008-2009	Poultry	362	96	Indiana	22
							Enteritidis	15
							Agona	6
							Albany	7
							Typhimurium	6
							Infantis	3
							Derby	1
							Essen	1
							Hadar	1
							Djugu	1
							Kentucky	17
							Shubra	15
							Haardt	1
4011	Serum agglutination	Gansu	2007-2009	Human	683	7	Typhimurium	7
4023	Serum agglutination	Guangdong	2006-2008	Food	369	18	Stanley	3
							Saintpaul	3
							Derby	2
							Agona	1
							Typhimurium	1
							Infantis	1
							Tshiongwe	1
							Others	1
							Corvallis	1
							Enteritidis	1
							Anatum	3
4035	Serum agglutination	Shanxi	2008-2009	Poultry	961	177	Gallinarum	177
4038	Serum agglutination	Shanxi	2002-2008	Food	1175	204	Aberdeen	1
							Bonariensis	1
							Coquilhatville	1
							Jerusalem	1
							Kentucky	1
							Kortrijk	1
							Kottbus	1
							Nchanga	1

								Pakistan	1
								Putten	1
								Regent	1
								Taksony	1
								Thompson	1
								Virchow	1
								Wernigerode	1
								Choleraesuis	2
								Meleagridis	2
								Muenster	2
								Tennessee	3
								Isangi	4
								London	6
								Lomita	7
								Anatum	10
								Enteritidis	21
								Senftenberg	51
								Others	81
4047	Serum agglutination	Zhejiang	2007-2009	Human	719	18		Typhimurium	9
								Agona	1
								Enteritidis	4
								Anatum	1
								Aberdeen	1
								Others	2
4057	Serum agglutination	Jiangxi	2008	Mouse	358	134		Typhimurium	134
4067	Serum agglutination	Jiangsu	2005-2007	Human	47132	67		Aberdeen	1
								Anatum	1
								Bury	1
								Choleraesuis	2
								Rissen	2
								Sendai	2
								Senftenberg	2
								Enteritidis	3
								Newport	4
								Essen	5
								Dublin	7
								Typhimurium	8
								Agona	11
								Derby	18
4088	Serum agglutination	Guangxi	2007-2008	Human	1400	43		Typhimurium	14
								Derby	3
								Enteritidis	1
								Kottbus	1
								Others	24
4094	Serum agglutination	Jiangsu	2008-2009	Food	1831	70		Reading	15
								Anatum	9
								Derby	8
								Typhimurium	6
								Senftenberg	4
								Choleraesuis	1
								Aberdeen	1
								Lomita	1
								Others	25
4121	Serum agglutination	Jiangsu	2010	Food	372	19		Derby	8
								Agona	4
								Typhimurium	3
								Dublin	3
								Essen	1
4126	Serum agglutination	Jiangxi	2007-2009	Human	1126	16		Typhimurium	6
								Paratyphi B	5
								Paratyphi A	3
								Enteritidis	2
4147	Serum agglutination	Beijing	2008	Human	786	38		Enteritidis	22
								Typhimurium	5
								Derby	3
								Choleraesuis	1
								Kouka	1
								Braenderup	1
								Agona	1
								Newport	1
								Istanbul	1
								Infantis	1
								Hadar	1
4159	Serum agglutination	Guangxi	2007	Human	19307	113		Derby	47
								Agona	11
								Stanley	7
								Typhimurium	3
								Saintpaul	1
								Indiana	1
								Paratyphi B	1
								Choleraesuis	7
								Thompson	2
								Kottbus	5
								Newport	1

							Enteritidis	3
							Newlands	1
							Anatum	2
							Others	21
4163	Serum agglutination	Fujian	2007	Human	2871	10	Enteritidis	2
							Typhimurium	4
							Choleraesuis	2
							Agona	1
							Derby	1
4169	Serum agglutination	Zhejiang	2001-2008	Human	47228	189	Give	16
							Others	24
							Infantis	27
							Agona	28
							Newlands	29
							Derby	65
4191	Serum agglutination	Zhejiang	2005-2007	Human	1143	47	Newport	1
							Others	4
							Paratyphi A	4
							Enteritidis	10
							Typhimurium	28
4199	NA	Shandong	2007-2009	Human	1100	20	Enteritidis	20
4206	Serum agglutination	Henan	2006-2007	Poultry	225	95	Enteritidis	55
							Typhimurium	8
							Indiana	16
							Derby	2
							Agona	2
							Hadar	3
							Thompson	2
							Aberdeen	1
							Albany	1
							Blegdam	1
							Eko	1
							Others	2
							Schwarzengrund	1
		Henan	2006-2007	Swine	154	26	Enteritidis	2
							Typhimurium	10
							Derby	7
							Agona	2
							Wandsworth	2
							Aberdeen	1
							Norwich	1
							Eko	1
4210	Serum agglutination	Zhejiang	2004-2007	Human	73601	217	Infantis	1
							Irumu	1
							Kaapstad	1
							Kottbus	1
							Litchfield	1
							Lomita	1
							Newlands	1
							Oritamerin	1
							Paratyphi C	1
							Sinstorf	1
							Bonn	2
							Chester	2
							Dublin	2
							Give	3
							Potsdam	3
							Stanley	3
							Newport	5
							Thompson	6
							Enteritidis	8
							Choleraesuis	10
							Saintpaul	11
							Muenster	12
							Anatum	14
							Typhimurium	16
							Agona	20
							London	20
							Derby	22
							Senftenberg	48
4230	Serum agglutination	Hunan	2007-2008	Food	130	47	Typhimurium	25
							Heidelberg	3
							Choleraesuis	7
							Thompson	2
							Enteritidis	9
							Dublin	1
4232	Serum agglutination, AIS	Chongqing	2007	Human	835	79	Reading	2
							Infantis	5
							Dublin	2
							Typhimurium	16
							Enteritidis	1
							Aberdeen	1
							Gothenburg	1

							Thompson	35
							Typhi	9
							Arizonae	1
							Paratyphi C	1
							Others	5
4239	Serum agglutination	Zhejiang	2003-2007	Human	2056	251	Agona	1
							Choleraesuis	1
							Newport	1
							Derby	3
							Enteritidis	6
							Paratyphi C	10
							Dublin	13
							Typhimurium	14
							Stanley	25
							Paratyphi A	69
							Typhi	108
4245	Serum agglutination	Guangdong	2005-2008	Food	635	49	Dumfries	1
							Newport	1
							Saintpaul	1
							Others	2
							Senftenberg	2
							Typhimurium	13
							Enteritidis	29
4246	Serum agglutination	Guangdong	2004-2009	Food	908	30	Lomita	1
							Meleagridis	1
							Choleraesuis	2
							Newport	2
							Senftenberg	2
							Typhimurium	2
							Agona	3
							Enteritidis	7
							Derby	10
4254	Serum agglutination	Beijing	2007	Human	313	34	Dublin	20
							Typhimurium	7
							Agona	1
							Derby	1
							Others	5
4261	Serum agglutination	Jiangsu	2007	Food	804	33	Derby	9
							Lomita	3
							Anatum	3
							Agona	2
							Enteritidis	2
							Saintpaul	1
							Typhimurium	1
							Newport	1
							London	1
							Others	10
4262	Serum agglutination	Shanxi	2002-2008	Food	522	33	Anatum	1
							Enteritidis	1
							Isangi	1
							Tennessee	1
							Virchow	1
							Lomita	2
							Others	2
							Senftenberg	5
							Agona	9
							Derby	10
4272	Serum agglutination	Guangxi	2007-2008	Human	35847	165	Derby	61
							Agona	21
							Stanley	7
							Typhimurium	7
							Paratyphi B	1
							Saintpaul	1
							Indiana	1
							Choleraesuis	14
							Thompson	4
							Montevideo	1
							Mbandaka	1
							Kottbus	5
							Newport	1
							Litchfield	3
							Enteritidis	7
							Anatum	3
							Newlands	2
							Give	1
							Others	24
4315	Serum agglutination	Shanghai	2004	Human	24286	116	London	41
							Senftenberg	21
							Agona	4
							Derby	9
							Typhimurium	2
							Choleraesuis	1
							Enteritidis	4
							Others	34

		Shanghai	2005	Human	25782	106	London	32
							Senftenberg	14
							Agona	10
							Derby	17
							Typhimurium	3
							Choleraesuis	3
							Enteritidis	2
							Others	25
		Shanghai	2006	Human	29251	90	London	13
							Senftenberg	9
							Agona	22
							Derby	6
							Typhimurium	2
							Choleraesuis	5
							Enteritidis	5
							Others	28
		Shanghai	2007	Human	36091	109	London	10
							Senftenberg	19
							Agona	9
							Derby	13
							Typhimurium	9
							Choleraesuis	8
							Enteritidis	7
							Others	34
4318	Serum agglutination	Shandong	2003-2008	Food	1833	38	Massenya	1
							Newport	1
							Others	1
							Thompson	1
							Senftenberg	4
							Enteritidis	30
4347	Serum agglutination	Henan	2008	Food	894	46	Typhimurium	12
							Choleraesuis	18
							Dublin	8
							Enteritidis	5
							Blegdam	3
4352	Serum agglutination	Shanghai	2006	Human	2564	40	Enteritidis	11
							Agona	6
							Derby	1
							Senftenberg	5
							Typhimurium	2
							Lomita	3
							Others	12
		Shanghai	2008	Human	2849	29	Enteritidis	6
							Agona	7
							Derby	7
							Typhimurium	2
							Others	7
4361	Serum agglutination	Beijing	2007	Human	1600	76	Dublin	50
							Derby	1
							Paratyphi B	1
							Bovismorbificans	1
							Potsdam	1
							Newport	1
							Anatum	1
							London	1
							Others	19
4374	Serum agglutination	Guangdong	2007	Human	1128	71	Enteritidis	17
							Typhimurium	10
							Stanley	5
							Lagos	3
							Thompson	3
							Derby	2
							Indiana	2
							London	2
							Sinchew	2
							Hittingfoss	1
							Others	24
4378	Serum agglutination	Guangdong	2001-2005	Human	151490	721	Derby	131
							Agona	59
							Thompson	21
							Infantis	20
							Kentucky	66
							Enteritidis	7
							Anatum	25
							Senftenberg	24
							Others	368
4380	NA	Hebei	2005	Food	386	81	Agona	11
							Senftenberg	8
							Derby	7
							Meleagridis	7
							Irumu	6
							Others	42
		Hebei	2006	Food	1135	50	Anatum	13
							Senftenberg	9

							Derby	7
							Choleraesuis	7
							Irumu	3
							Sinstorf	2
							Lomita	2
							London	2
							Others	5
		Hebei	2007	Food	537	12	Enteritidis	4
							Senftenberg	1
							London	1
							Choleraesuis	1
							Anatum	1
							Saintpaul	1
							Bata	1
							Derby	1
							California	1
4387	Serum agglutination	Sichuan	2007	Human	338	18	Typhimurium	8
							Derby	5
							Enteritidis	4
							Stanley	1
4391	Serum agglutination	Zhejiang	2004-2007	Human	26318	69	Canada	1
							Irumu	1
							Muenchen	1
							Paratyphi A	1
							Stanley	1
							Hissar	2
							Sendai	3
							Senftenberg	3
							Edinburg	4
							Newlands	5
							Others	4
							Give	6
							Derby	37
4402	Serum agglutination	Shanghai	2006	Human	4856	29	Typhimurium	7
							Senftenberg	6
							Agona	5
							Lomita	3
							Others	8
4409	Serum agglutination	Shanghai	2004	Human	24286	116	London	41
							Senftenberg	21
							Agona	4
							Derby	8
							Senftenberg	13
							Agona	10
							Derby	17
							Enteritidis	2
		Shanghai	2006	Human	29251	90	London	13
							Senftenberg	9
							Agona	22
							Derby	6
							Enteritidis	5
							Others	35
4415	Serum agglutination	Guangdong	2004-2007	Human	3257	60	Typhimurium	60
4447	Serum agglutination	Guangxi	2005	Food	682	73	Derby	32
							Agona	5
							Typhimurium	1
							Indiana	2
							Dublin	1
							Senftenberg	4
							Chester	2
							Give	1
							Anatum	1
							Newlands	1
							Others	23
4449	Serum agglutination	Guangxi	2006-2007	Food	464	11	Derby	3
							Agona	4
							Paratyphi C	1
							Senftenberg	1
							Others	2
4466	Serum agglutination	Shanxi	2002-2006	Food	2272	208	Senftenberg	39
							Derby	27
							Agona	19
							Others	67
							Senftenberg	14
							Derby	10
							Agona	7
							Others	25
4469	Serum agglutination	Hebei	2005-2007	Food	2058	144	Manhattan	4
							Sinstorf	4
							London	7
							Meleagridis	7
							Enteritidis	8
							Irumu	8
							Choleraesuis	9

							Agona	11
							Anatum	15
							Derby	15
							Senftenberg	18
							Others	38
4492	Serum agglutination	Fujian	2006	Human	51548	86	Agona	24
							Enteritidis	20
							Derby	18
							Typhimurium	13
							Anatum	5
							Senftenberg	3
							Saintpaul	1
							Stanley	1
							Chester	1
4535	Serum agglutination	Shanghai	2006	Human	3603	196	Enteritidis	44
							Typhimurium	35
							Senftenberg	34
							Aberdeen	10
							Derby	9
							Lomita	7
							Agona	6
							Infantis	6
							Anatum	4
							Saintpaul	3
							Muenster	3
							Stanley	2
							Hadar	3
							Singapore	2
							Pomona	2
							Others	26
4655	Serum agglutination	Liaoning	2003-2005	Food	1965	6	Paris	1
							Agona	1
							Bonariensis	2
							Newport	2
4666	Serum agglutination	Guangdong	2004	Food	550	31	Derby	7
							Agona	5
							Typhimurium	2
							Newport	2
							Lomita	1
							Enteritidis	11
							Meleagridis	2
							Senftenberg	1
4676	Serum agglutination	Shanghai	2006	Human	4856	29	Agona	5
							Newport	1
							Senftenberg	6
							Calabar	2
							Typhimurium	7
							Lomita	3
							Mbandaka	1
							Aberdeen	2
							Anatum	1
							Derby	1
4688	Serum agglutination	Zhejiang	2004-2005	Human	384	2	Thompson	1
							Typhimurium	1
4690	Serum agglutination	Shandong	2004	Human	38030	187	Derby	83
							Typhimurium	2
							Choleraesuis	17
							Newport	22
							Enteritidis	23
							Anatum	29
							Senftenberg	11
		Shandong	2005	Human	40271	486	Derby	252
							Typhimurium	2
							Choleraesuis	48
							Newport	64
							Enteritidis	46
							Anatum	60
							Senftenberg	4
							Aberdeen	10
		Shandong	2006	Human	48006	378	Derby	149
							Typhimurium	1
							Choleraesuis	52
							Newport	37
							Enteritidis	51
							Anatum	32
							Senftenberg	6
							Aberdeen	42
							Others	8
4694	Serum agglutination	Shandong	2005-2006	Human	636	17	Agona	1
							Anatum	1
							Thompson	1
							Derby	2
							Paratyphi B	5
							Typhimurium	7

4707	Serum agglutination	Liaoning	2006	Swine	333	210	Derby	61
							Typhimurium	13
							Saintpaul	1
							Kingston	1
							Paratyphi B	2
							Infantis	9
							Thompson	1
							Newport	1
							Manhattan	2
							Anatum	68
							Meleagridis	8
							London	4
							Eschberg	1
							Windsheim	1
							Taksony	1
							Senftenberg	1
							Newlands	1
							Others	34
4740	Serum agglutination	Shanghai	2007	Human	165142	186	Derby	35
							Agona	18
							Saintpaul	6
							Typhimurium	6
							Paratyphi B	2
							Reading	1
							Kingston	1
							Thompson	8
							Infantis	3
							Mbandaka	2
							Oranienburg	1
							Montevideo	1
							Newport	8
							Enteritidis	8
							London	9
							Anatum	7
							Muenster	3
							Meleagridis	1
							Senftenberg	39
							Reading	1
							Krefeld	1
							Aberdeen	5
							Teko	1
							Others	19
4754	Serum agglutination	Jiangsu	2002-2006	Food	484	50	Derby	50
4771	Serum agglutination	Hebei	2005	Food	386	81	Agona	11
							Senftenberg	8
							Derby	7
							Meleagridis	7
							Irumu	6
							Choleraesuis	5
							Manhattan	4
							London	4
							Enteritidis	4
							Saintpaul	3
							Indiana	3
							Bredeney	1
							Calabar	1
							Muenster	2
							Newlands	2
							Sinstorf	2
							Poona	2
							Albany	2
							Montevideo	2
							Kingston	1
							Thompson	1
							Anatum	1
							Others	2
4797	Serum agglutination	Guangxi	2002-2005	Food	1872	128	Derby	43
							Weltevreden	9
							Senftenberg	6
							Kentucky	6
							Agona	4
							Chester	3
							Indiana	4
							Typhimurium	3
							Saintpaul	2
							Schwarzengrund	1
							Newlands	2
							Enteritidis	2
							Tshiongwe	1
							Dublin	1
							Give	1
							Anatum	1
							Others	39
4848	Serum agglutination	Shanghai	2002-2003	Food	303	112	Derby	33

							Enteritidis	27
							Typhimurium	8
							Anatum	8
							Reading	8
							Senftenberg	7
							Agona	4
							Saintpaul	4
							London	3
							Kentucky	3
							Wandsworth	1
							Others	6
4860	Serum agglutination	Guangdong	2000-2004	Cold-bloo	4671	27	Agona	1
							Stanley	1
							Typhimurium	1
							Choleraesuis	2
							Newport	10
							Others	11
							Choleraesuis	1
		Guangdong	2000-2004	Feed	60	3	Others	3
4871	Serum agglutination	Guangdong	1999-2004	Human	89286	233	Chester	13
							Tshiongwe	20
							Agona	31
							Derby	37
							Others	132
4875	Serum agglutination	Ningxia	2001-2006	Human	1942	30	Typhimurium	30
4907	Serum agglutination	Henan	2006	Food	540	57	Indiana	30
							Enteritidis	7
							Agona	7
							Typhimurium	7
							Haardt	1
							Derby	1
							London	1
							Edinburg	1
							Others	2
4914	Serum agglutination	Jiangsu	2002-2004	Food	957	20	Derby	10
							Agona	4
							Senftenberg	2
							Enteritidis	1
							Newport	2
							Others	1
4926	Serum agglutination	Guangdong	1998-2003	Human	511184	239	Bareilly	1
							Blegdam	1
							Bovismorbificans	1
							Farsta	1
							Istanbul	1
							Lagos	1
							Nchanga	1
							Sanjuan	1
							Weltevreden	1
							Infantis	2
							Paratyphi A	2
							Others	2
							Meleagridis	3
							Paratyphi B	3
							Albany	4
							Dublin	4
							Haardt	4
							Manhattan	4
							Virchow	4
							Thompson	7
							Chester	10
							Newlands	11
							Stanley	11
							Bonn	13
							Tshiongwe	14
							Enteritidis	29
							Agona	47
							Derby	56
4936	Serum agglutination	Guangdong	2002-2005	Human	452	8	Typhimurium	8
5125	Serum agglutination	Hubei	2003-2004	Human	693	9	Derby	3
							Agona	1
							Typhimurium	2
							Enteritidis	3
5151	Serum agglutination	Hebei	1998-2002	Human	36000	648	Newlands	38
							Muenster	39
							Derby	66
							Agona	139
							Others	366
5157	Serum agglutination	Shanghai	2001-2003	Human	283685	282	Agona	65
							Derby	34
							Gaillac	20
							Senftenberg	16
							Enteritidis	11
							Others	136

5158	Serum agglutination	Fujian	2000	Food	332	12	Derby	3
							Enteritidis	7
							Agona	1
							Typhimurium	1
5184	Serum agglutination	Jiangsu	2002-2003	Food	450	10	Derby	4
							Agona	1
							Senftenberg	2
							Enteritidis	1
							Newport	2
5368	Serum agglutination	Sichuan	2002	Human	72111	45	Paratyphi A	11
							Derby	8
							Agona	5
							Anatum	4
							Enteritidis	3
							Braenderup	3
							Others	3
							Virchow	3
							Indiana	2
							Abortusequi	3
5370	Serum agglutination	Zhejiang	2001	Human	12850	25	Stanley	1
							Reading	1
							Virchow	1
							Thompson	1
							Cayar	1
							Bardo	2
							Newport	3
							Kottbus	1
							Loanda	1
							Newlands	1
							Sinstorf	1
							Senftenberg	6
							Others	5
5375	Serum agglutination	Jiangxi	2001	Human	15420	193	Stanley	3
							Eppendorf	1
							Schwarzengrund	2
							Saintpaul	2
							Reading	1
							Derby	101
							Agona	12
							Indiana	1
							Larochelle	1
							Lomita	4
							Nola	1
							Thompson	1
							Massakory	3
							Mbandaka	3
							Redba	1
							Newport	4
							Kottbus	6
							Tshiongwe	4
							Others	2
							Kentucky	1
							Hadar	1
							Wohlen	1
							Enteritidis	5
							Muenster	2
							Newlands	1
							Meleagridis	15
							Calabar	1
							Rideau	11
							Maiduguri	1
							Senftenberg	1
5376	Serum agglutination	Fujian	2000-2002	Food	978	64	Anatum	1
							Heidelberg	1
							Kottbus	1
							Reading	1
							Litchfield	2
							Senftenberg	2
							London	3
							Newport	3
							Enteritidis	7
							Typhimurium	9
							Agona	10
							Weltevreden	11
							Derby	13
5394	Serum agglutination	Shaanxi	1997-2002	Human	685	8	Typhimurium	8
5396	Serum agglutination	Shaanxi	2002	Food	468	44	Senftenberg	16
							Agona	8
							Derby	6
							Haardt	2
							Meleagridis	1
							Rissen	1
							London	1
							Others	9

5397	Serum agglutination	Beijing	2002	Food	927	32	Enteritidis	8
							Gallinarum	3
							Haardt	4
							Infantis	2
							Agona	1
							Muenster	1
							Senftenberg	1
							Stanley	1
							Derby	2
							Braenderup	1
							Reading	1
							Mbandaka	1
							Liverpool	1
							Skiwerin	1
							Chartres	1
							Naestved	1
							Others	2
5400	Serum agglutination	Jiangsu	1999-2001	Human	90728	262	Kottbus	1
							Newport	1
							Suberu	1
							Thompson	1
							Anatum	2
							Regent	2
							Typhimurium	2
							Enteritidis	3
							Lomita	3
							Chester	4
							Reading	6
							Give	7
							Newlands	8
							Agona	66
							Derby	69
							Others	86
5413	Serum agglutination	Guangdong	2000-2001	Human	50630	130	Anatum	1
							Bonariensis	1
							Chester	1
							Gatineau	1
							Kouka	1
							Meleagridis	1
							Thompson	1
							Blegdam	2
							Chailey	2
							Glostrup	2
							Infantis	2
							Lomita	2
							Muenster	2
							Senftenberg	2
							Sinstorf	2
							Typhimurium	2
							Blegdam	3
							Dublin	3
							Haardt	3
							Rissen	3
							Newlands	4
							Weltevreden	4
							Ferruch	5
							Stanley	5
							Others	8
							Derby	11
							Agona	12
							Enteritidis	12
							Rideau	14
							Tshiongwe	18
5414	Serum agglutination	Hubei	1997-2000	Human	45353	558	Paratyphi A	7
							Nitra	1
							Derby	131
							Agona	94
							Typhimurium	3
							Sandiego	12
							Eko	6
							Chester	5
							Schwarzengrund	2
							Eppendorf	1
							Reading	34
							Thompson	20
							Potsdam	3
							Infantis	4
							Isangi	2
							Singapore	7
							Lomita	4
							Rissen	6
							Manhattan	12
							Newport	23
							Cremieu	1

							Kottbus	18
							Pullorum	3
							Enteritidis	22
							Naestved	1
							Butantan	4
							Muenster	13
							Anatum	92
							Newlands	6
							Regent	1
							London	3
							Meleagridis	12
							Senftenberg	5
5474	Serum agglutination	Jiangsu	2001	Food	341	14	Derby	8
							Agona	1
							Bovismorbificans	1
							Thompson	1
							Choleraesuis	1
							Bardo	1
							Aberdeen	1
5510	Serum agglutination	Shaanxi	1998-2000	Human	2489	36	Typhimurium	33
							Others	3
5518	Serum agglutination	Guangdong	1999-2000	Human	10083	27	Derby	12
	Serum agglutination						Others	15
5520	Serum agglutination	Inner Mongolia	2000	Poultry	12497	1688	Gallinarum	1688
5623	Serum agglutination	Guangdong	1997-1999	Human	126228	1051	Derby	265
	Serum agglutination						Others	786
5625	Serum agglutination	Sichuan	1999	Rabbit	38	3	Derby	2
							Manhattan	1
		Sichuan	1999	Poultry	132	2	Derby	2
		Sichuan	1999	Cold-blooded animals	116	16	Derby	14
							Anatum	2
		Sichuan	1999	Swine	212	92	Derby	51
							Typhimurium	23
							Anatum	16
							Kottbus	1
							Others	1
5627	Serum agglutination	Guangdong	1997-1998	Human	67885	152	Ferruch	5
							Chester	9
							Agona	24
							Wien	6
							Bareilly	6
							Newlands	5
							Derby	37
							Mbandaka	1
							Vayle	1
							Anatum	4
							London	6
							Weltevreden	2
							Blegdam	7
							Stanley	4
							Kisangani	3
							Senftenberg	5
							Rissen	1
							Kouka	1
							Daytona	1
							Indiana	1
							Pakistan	1
							Manhattan	1
							Tshiongwe	3
							Portsmouth	1
							Moscow	3
							Westhampton	2
							Others	12
5664	Serum agglutination	Fujian	1991-1994	Human	887	73	Typhimurium	73
5665	Serum agglutination	Guangdong	1989-1993	Human	249496	748	Choleraesuis	1
							Eastbourne	1
							Manhattan	1
							Muenchen	1
							Papuana	1
							Paratyphi A	1
							Reading	1
							Saintpaul	1
							Tshiongwe	1
							Infantis	2
							Litchfield	2
							Heidelberg	4
							Others	8
							Miami	5
							Mbandaka	6
							Enteritidis	7
							Tennessee	8
							London	9

							Thompson	11
							Sendai	12
							Meleagridis	16
							Agona	20
							Newlands	21
							Newport	27
							Typhi	28
							Weltevreden	35
							Potsdam	37
							Bovismorbificans	48
							Stanley	52
							Typhimurium	81
							Anatum	98
							Derby	202
5684	Serum agglutination	Fujian	1989-1999	Human	21053	222	Bardo	1
							Chailey	1
							Livingstone	1
							Saintpaul	1
							Bovismorbificans	2
							Escanaba	2
							Montevideo	2
							Muenster	2
							Senftenberg	2
							Taksony	2
							Bareilly	3
							Others	6
							Paratyphi B	3
							Thompson	5
							Reading	6
							Meleagridis	7
							Stanley	7
							Agona	8
							Mbandaka	8
							Singapore	8
							Newport	9
							Typhimurium	11
							Weltevreden	12
							Enteritidis	13
							Blockley	14
							Anatum	17
							London	23
							Derby	46
5705	Serum agglutination	Anhui	1997-1998	Human	13472	301	Agona	41
							Anatum	34
							Thompson	29
							Kottbus	27
							Pottos	19
							Potsdam	18
							Aberdeen	17
							Typhimurium	15
							Meleagridis	14
							Bonn	8
							London	6
							Others	20
							Derby	53
5706	Serum agglutination	Henan	1998	Swine	1820	113	London	38
							Agona	34
							Choleraesuis	15
							Typhimurium	8
							Derby	7
							Djakarta	7
							Manhattan	2
							Meleagridis	1
							Anatum	1
5710	Serum agglutination	Sichuan	1997	Human	30364	135	Derby	52
							Typhimurium	42
							Anatum	12
							Enteritidis	10
							Agona	10
							Paratyphi A	2
							Others	7
5743	Serum agglutination	Fujian	1994-1995	Human	21053	222	Derby	46
							Typhimurium	11
							Agona	8
							Stanley	7
							Reading	6
							Paratyphi B	3
							Saintpaul	1
							Mbandaka	8
							Singapore	8
							Thompson	5
							Bareilly	3
							Escanaba	2
							Montevideo	2

							Livingstone	1
							Blockley	14
							Newport	9
							Bovismorbificans	2
							Chailey	1
							Bardo	1
							Enteritidis	13
							London	23
							Anatum	17
							Weltevreden	12
							Meleagridis	7
							Muenster	2
							Senftenberg	2
							Taksony	2
							Others	6
5759	Serum agglutination	Shanghai	1992-1996	Human	99729	274	Choleraesuis	1
							Potsdam	1
							Saintpaul	1
							Stanley	1
							Typhi	1
							Cambridge	2
							Paratyphi C	3
							Senftenberg	3
							Enteritidis	4
							Paratyphi B	5
							Thompson	5
							Paratyphi A	6
							Anatum	8
							Meleagridis	8
							Typhimurium	8
							London	10
							Manhattan	12
							Newport	24
							Derby	41
							Agona	130
5827	Serum agglutination	Beijing	1991-1995	Human	8126	167	Enteritidis	69
							Typhimurium	24
							Agona	14
							Paratyphi B	7
							Newport	6
							Derby	5
							Braenderup	5
							Cambridge	5
							Larochelle	3
							Lomita	3
							Sonjuan	2
							Thompson	2
							Kottbus	2
							Typhi	2
							London	2
							Senftenberg	2
							Horsham	2
							Paratyphi A	1
							Albany	1
							Kunduchi	1
							Montevideo	1
							Norwich	1
							Infantis	1
							Narashino	1
							Blockley	1
							Muenster	1
							Juba	1
							Rideau	1
							Others	1
5849	Serum agglutination	Guangdong	1991-1993	Human	50808	504	Paratyphi A	1
							Paratyphi B	1
							Stanley	46
							Saintpaul	12
							Reading	1
							Chester	1
							Derby	77
							Agona	131
							Typhimurium	9
							Oslo	1
							Larochelle	1
							Braenderup	5
							Rissen	1
							Thompson	3
							Daytona	1
							Concord	2
							Potsdam	3
							Virchow	2
							Infantis	3
							Mbandaka	4

							Tennessee	18
							Leer	1
							Muenchen	2
							Manhattan	6
							Bardo	3
							Newport	31
							Kottbus	4
							Tshiongwe	1
							Haardt	3
							Blockley	26
							Pakistan	1
							Litchfield	3
							Bovismorbificans	5
							Chailey	2
							Hadar	2
							Glostrup	1
							Sendai	1
							Enteritidis	4
							Dublin	1
							Javiana	2
							Vayle	1
							Muenster	2
							Anatum	21
							Newlands	3
							Meleagridis	4
							Zanzibar	1
							Newrochelle	1
							London	2
							Weltevreden	17
							Senftenberg	27
							Taksony	1
							Krefeld	1
							Others	1
5866	Serum agglutination	Shanxi	1989-1993	Swine	1159	305	Senftenberg	1
							Kentucky	2
							Newport	3
							Stanley	5
							Agona	6
							Meleagridis	8
							Choleraesuis	11
							London	12
							Dublin	18
							Thompson	27
							Enteritidis	35
							Typhimurium	37
							Anatum	52
							Derby	88
		Shanxi	1989-1993	Poultry	300	65	Enteritidis	1
							Meleagridis	1
							Ohio	1
							Thompson	1
							London	3
							Typhimurium	13
							Anatum	20
							Derby	25
5877	Serum agglutination	Zhejiang	1983-1992	Human	30684	179	Agona	1
							Bonn	1
							Concord	1
							Enteritidis	1
							Javiana	1
							Lomita	1
							Newport	1
							Paratyphi A	1
							Paratyphi B	1
							Ruzizi	1
							Tshiongwe	3
							Typhimurium	3
							London	4
							Potsdam	5
							Stanley	7
							Derby	12
							Typhi	12
							Anatum	35
							Senftenberg	35
							Newlands	53
5878	Serum agglutination	Tibet	1993	Human	626	11	Typhimurium	3
							Bovismorbificans	8
5892	Serum agglutination	Shandong	1993	Human	402	5	Newport	1
							Derby	1
							Sendai	1
							Agona	2
5916	Serum agglutination	Jiangxi	1989-1990	Cold-blooded animals	590	367	Stanley	10

							Derby	2
							Others	224
							Thompson	28
							Potsdam	14
							Newport	18
							Senftenberg	1
							Surat	5
							Albany	1
							Weston	1
							Zongo	1
							Ramatgan	61
							Wandsworth	1
5937	Serum agglutination	Zhejiang	1992	Food	484	85	Paratyphi A	3
							Typhimurium	8
							Agona	7
							Shubra	9
							Bonn	3
							Potsdam	2
							Cremieu	2
							Istanbul	22
							Bardo	8
							Anatum	11
							Others	10
5944	Serum agglutination	Guangdong	1990-1991	Human	47580	382	Bonn	1
							Braenderup	1
							Glostrup	1
							Limete	1
							Meleagridis	1
							Muenchen	1
							Potsdam	1
							Rissen	1
							Southampton	1
							Texas	1
							Thompson	1
							Litchfield	2
							Papuana	2
							Reading	2
							Bovismorbificans	3
							Cairo	3
							Infantis	3
							Mbandaka	3
							Newlands	3
							Typhimurium	3
							Saintpaul	6
							Weltevreden	14
							Stanley	15
							Kottbus	21
							Others	40
							Newport	25
							Tennessee	26
							Anatum	51
							Agona	56
							Derby	93
5956	Serum agglutination	Qinghai	1992	Swine	339	55	Paratyphi A	4
							Kisangani	7
							Typhimurium	4
							Paratyphi B	3
							Agona	2
							Choleraesuis	6
							Oslo	3
							Bonn	1
							Muenster	12
							London	4
							Newlands	5
							Weltevreden	2
							Ughelli	1
							Newrochelle	1
6174	Serum agglutination	Qinghai	1997	Human	1192	8	Saintpaul	3
							Enteritidis	1
							Anatum	2
							Others	2
6203	Serum agglutination	Anhui	1995-1996	Human	660	20	London	1
							Aberdeen	2
							Agona	2
							Anatum	2
							Enteritidis	2
							Typhimurium	2
							Bovismorbificans	4
							Derby	5
6211	Serum agglutination	Jiangsu	1995	Human	6507	94	Derby	21
							Agona	9
							Typhimurium	2
							Typhi	1
							Anatum	2

							London	2
							Senftenberg	56
							Vienna	1
6225	Serum agglutination	Henan	1990	Human	372	105	Typhimurium	105
6247	NA	Henan	1992	Human	703	105	Typhimurium	105
6252	Serum agglutination	Hunan	1988-1990	Human	1638	76	Enteritidis	32
							Typhimurium	31
							Choleraesuis	8
							Paratyphi B	3
							Typhi	1
							Gallinarum	1
6254	Serum agglutination	Gansu	1989-1992	Poultry	766	70	Gallinarum	63
							Others	7
6260	Serum agglutination	Guangdong	1990-1991	Human	16611	57	Bredeney	8
							Derby	13
							Others	17
							Agona	19
6264	Serum agglutination	Zhejiang	1978-1990	Human	22914	176	Aberdeen	1
							Bonn	1
							Gallinarum	1
							Manhattan	1
							Others	1
							Taksony	1
							Chennai	2
							Choleraesuis	2
							Senftenberg	2
							Stanley	2
							Paratyphi A	3
							Typhimurium	3
							Thompson	4
							Montevideo	5
							Meleagridis	7
							Newport	7
							Paratyphi B	8
							Enteritidis	11
							Agona	23
							Potsdam	25
							Derby	31
							Anatum	35
6279	Serum agglutination	Zhejiang	1988-1989	Human	2659	8	Agona	3
							Typhimurium	2
							Derby	1
							Manhattan	1
							Arizonae	1
6308	Serum agglutination	Shandong	1986-1989	Human	511	5	Typhimurium	2
							Typhi	1
							Agona	1
							London	1
6326	Serum agglutination	Fujian	1988	Swine	453	152	Agona	6
							Stanley	2
							Derby	84
							Newport	4
							Anatum	49
							London	6
							Taksony	1
		Fujian	1988	Ruminants	33	10	Agona	1
							Derby	5
							Anatum	2
							Agona	1
							Anatum	1
		Fujian	1988	Poultry	61	6	Typhimurium	1
							Derby	2
							Newport	2
							Anatum	1
6336	Serum agglutination	Henan	1987-1988	Human	1253	40	Typhimurium	28
							Paratyphi B	5
							Others	7
6346	Serum agglutination	Anhui	1985-1987	Human	29549	256	Derby	40
							Typhimurium	40
							Agona	37
							Lagos	1
							Copenhagen	8
							Gloucester	1
							Others	16
							Thompson	5
							Concord	5
							Newport	17
							Muenchen	2
							Kottbus	1
							Labadi	1
							Curacao	1
							Enteritidis	1
							Anatum	34

							Newlands	9
							London	2
							London	2
							Cambridge	1
							Greiz	2
							Taksony	1
							Senftenberg	1
							Stratford	4
							Aberdeen	17
							Abaetetuba	4
							Rubislaw	3
6393	Serum agglutination	Hunan	1984	Food	5200	741	Paratyphi A	1
							Paratyphi B	2
							Derby	116
							Agona	22
							Saintpaul	1
							Typhimurium	82
							Stanley	35
							Thompson	10
							Potsdam	2
							Newport	98
							Bovismorbificans	9
							Typhi	20
							Enteritidis	19
							Anatum	232
							Newlands	28
							Meleagridis	35
							London	7
							Cambridge	1
							Senftenberg	19
							Others	2
6430	Serum agglutination	Hunan	1984	Human	435	9	Typhimurium	5
							Derby	1
							Paratyphi B	1
							Others	1
							Eko	1
6609	Serum agglutination	Taiwan	2005-2007	Dogs	1109	168	Amsterdam	1
							Kouka	1
							Orion	1
							Saintpaul	1
							Schwarzengrund	1
							Taksony	1
							Tananarive	1
							Weltevreden	1
							Itami	2
							Makumira	2
							Stanley	2
							Albany	4
							Anatum	4
							Assinie	4
							Duesseldorf	4
							Derby	5
							Javiana	5
							Typhimurium	5
							Agona	6
							Lanka	6
							Senftenberg	7
							Enteritidis	19
							Newport	28
							Others	57
6669	Serum agglutination	Zhejiang	2017-2020	Human	1295	103	Typhimurium	40
							Enteritidis	26
							London	4
							Bovismorbificans	3
							Others	30
6673	Serum agglutination	Hunan	2015-2019	Human	7506	732	Agona	2
							Albany	2
							Anatum	2
							Virchow	2
							Wandsworth	2
							Bovismorbificans	3
							Kentucky	3
							Singapore	4
							Infantis	6
							Litchfield	6
							Newport	8
							Rissen	8
							Meleagridis	9
							London	12
							Stanley	13
							Thompson	18
							Goldcoast	19
							Others	25
							Enteritidis	41

							Derby	48
							Typhimurium	499
6685	Serum agglutination	Hebei	2019-2020	Poultry	588	150	Pullorum	92
							Enteritidis	36
							Paratyphi A	17
							Paratyphi B	2
							Aberdeen	1
							Others	2
6712	AIS	Taiwan	2014	Poultry	622	156	Albany	65
							Schwarzengrund	32
							Kentucky	20
							Tennessee	8
							Livingstone	5
							Muenster	5
							Newport	5
							Haardt	3
							Lindenberg	3
							Enteritidis	2
							Montevideo	2
							Typhimurium	2
							Livingstone	2
							Hadar	1
							Havana	1
6733	PFGE	Taiwan	2014-2016	Poultry	2040	243	Hardar	47
							Typhimurium	35
							Livingstone	11
							Albany	85
							Tennessee	1
							Schwarzengrund	56
							Stanley	3
							Newport	3
							Weltevreden	2
6788	PCR	Taiwan	2008	Poultry	1121	110	Montevideo	2
							Potsdam	107
							Albany	1
6791	Serum agglutination	Taiwan	2005	Swine	440	58	Anatum	25
							Derby	6
							Typhimurium	2
							Schwarzengrund	4
							Choleraesuis	7
							Weltevreden	3
							Enteritidis	2
							Newport	1
							Dusseldorf	0
							Bardo	0
							Bonn	0
							Senftenberg	1
							Gloucester	1
							Agona	1
							Others	5
6806	Serum agglutination	Taiwan	2002-2003	Poultry	1595	164	Derby	1
							Kubacha	4
							Mons	30
							Typhimurium	5
							Choleraesuis	61
							Grampian	10
							Hissar	1
							Others	15
							Redba	1
							Blockley	1
							Albany	10
							Enteritidis	18
							Anatum	4
							Havana	3
6837	Serum agglutination	Taiwan	2003-2005	Dogs	928	40	Branderburg	1
							Derby	4
							Eppendorf	1
							Essen	2
							Fyris	1
							Lagos	1
							Schwarzengrund	1
							Stanley	1
							Typhimurium	1
							Bardo	3
							Bellevue	1
							Dusseldorf	9
							Newport	1
							Enteritidis	5
							Itami	1
							Panama	1
							Goelzau	1
							Weltevreden	1
							Others	4
6877	Serum agglutination	Taiwan	2000-2001	Poultry	2000	91	Potsdam	29

							Others	7
							Montevideo	2
							Dusseldorf	17
							Hadar	5
							Newport	4
							Indiana	13
							Typhimurium	7
							Derby	4
							Schwarzengrund	2
							Assinie	1
6990	Serum agglutination	Guangdong	2012-2013	Poultry	113	29	Derby	11
							London	2
							Heidelberg	6
							Rosenthal	3
							Typhimurium	2
							Indiana	2
							Enteritidis	1
							Bovismorbificans	1
							London	1
		Guangdong	2012-2013	Swine	204	53	Derby	19
							Typhimurium	11
							Rosenthal	3
							Senftenberg	2
							London	2
							Bovismorbificans	1
							Virchow	1
							Indiana	1
							Others	13
7039	Serum agglutination	Hong Kong	1976	Swine	1026	344	Anatum	255
							Derby	58
							Typhimurium	12
							London	7
							Choleraesuis	5
							Newport	5
							Meleagridis	2
7043	Serum agglutination	Hong Kong	1953-1966	Human	105290	477	Choleraesuis	49
							Others	428
7063	Serum agglutination	Henan	2014-2015	Poultry	2139	45	Enteritidis	15
							Cerro	9
							Thompson	14
							Tennessee	3
							Indiana	3
							Kottbus	1
7080	PCR	Shanghai	2018-2019	Swine	1389	239	Typhimurium	75
							Derby	52
							Rissen	39
							Mbandaka	30
							London	16
							Kentucky	14
							Others	13
7085	WGS	Sichuan	2019	Poultry	420	74	Enteritidis	12
							Derby	2
							Mbandaka	26
							Indiana	13
							Kentucky	13
							Hadar	2
							Thompson	1
							Others	5
7094	Serum agglutination	Jiangsu	2018-2019	Dogs and	469	27	Kentucky	11
							Typhimurium	5
							Indiana	5
							Derby	3
							Sandiego	1
							London	1
							Rissen	1
7098	Serum agglutination	Shandong	2019-2020	Poultry	360	155	Gallinarum	128
							Enteritidis	27
7101	Serum agglutination	Shandong	2015-2017	Poultry	1288	86	Thompson	32
							Infantis	28
							Enteritidis	25
							Manhattan	1
7110	Serum agglutination	Shandong	2013-2018	Poultry	923	280	Enteritidis	128
							Kentucky	42
							Typhimurium	27
							Amager	19
							Gallinae	14
							Abortusequi	2
							Concord	2
							Blegdam	1
							Dublin	1
							Hissar	1
							Tsevie	1
							Orion	1
							Okerara	1

							London	1
							Derby	1
							Sinstorf	1
							Others	37
7125	Serum agglutination	Guangdong	2013	Food	317	82	Derby	32
							Typhimurium	17
							Rissen	5
							Agona	1
							London	4
							Mbandaka	1
							Enteritidis	16
							Others	6
		Guangdong	2014	Food	440	157	Derby	52
							Typhimurium	13
							Rissen	20
							Agona	3
							London	20
							Stanley	5
							Meleagridis	9
							Others	35
		Guangdong	2015	Food	754	287	Derby	74
							Typhimurium	38
							Rissen	41
							Corvallis	19
							Agona	22
							London	10
							Mbandaka	12
							Stanley	12
							Kentucky	6
							Enteritidis	2
							Meleagridis	2
							Others	49
		Guangdong	2016	Food	1107	445	Derby	81
							Typhimurium	89
							Rissen	24
							Corvallis	39
							Agona	37
							London	22
							Mbandaka	21
							Stanley	7
							Kentucky	17
							Enteritidis	2
							Meleagridis	4
							Give	5
							Others	97
		Guangdong	2017	Food	371	145	Derby	30
							Typhimurium	32
							Rissen	14
							Corvallis	9
							Agona	6
							London	9
							Stanley	1
							Kentucky	15
							Enteritidis	4
							Meleagridis	3
							Others	22
7127	Serum agglutination	Shandong	2018	Poultry	600	67	Enteritidis	37
							Typhimurium	18
							Kentucky	4
							Indiana	3
							Gallinarum	3
							Senftenberg	2
7145	Serum agglutination	Shaanxi	2013-2014	Poultry	814	61	Typhimurium	15
							Indiana	14
							Thompson	8
							Enteritidis	7
							Norwich	3
							Virchow	3
							Derby	2
							Senftenberg	2
							Infantis	2
							Albany	2
							Blockley	1
							Mbandaka	1
							Braenderup	1
7173	Serum agglutination	Guangdong	2017-2019	Poultry	365	151	Molade	1
							Bareilly	1
							Goldcoast	1
							Bovismorbificans	1
							Anatum	1
							London	1
							Senftenberg	1
							Stanley	1
							Tennessee	1

							Albany	2
							Infantis	2
							Hadar	3
							Weltevreden	3
							Enteritidis	4
							Rissen	4
							Cerro	4
							Derby	5
							Braenderup	6
							Mbandaka	6
							Typhimurium	6
							Saintpaul	8
							Thompson	8
							Indiana	14
							Agona	20
							Kentucky	22
							Corvallis	25
7177	Serum agglutination	Hubei	2016	Swine	1440	177	Derby	79
							Rissen	55
							Typhimurium	24
							Chester	5
							Mantis	2
							Virchow	1
							Stanley	1
							London	1
							Anatum	1
							Abony	1
							Tumodi	1
							Eastbourne	1
							Newlands	1
							Typhimurium	1
							Typhimurium	1
							Others	1
							Rissen	1
7185	Serum agglutination	Jiangsu	2016-2017	Swine	459	80	Derby	35
							Typhimurium	9
							Chester	2
							Rissen	16
							Newlands	11
							Sinstorf	3
							Nchanga	3
							Others	1
7197	Serum agglutination	Shanghai	1998-2017	Human	32544	565	Typhimurium	122
							Enteritidis	95
							London	37
							Thompson	8
							Saintpaul	6
							Others	297
7201	Serum agglutination	Guangdong	2017-2018	Poultry	1000	54	Agona	3
							Derby	5
							Enteritidis	14
							Give	4
							Heidelberg	2
							Meleagridis	3
							Gallinarum	12
							Rissen	5
							Senftenberg	3
							Typhimurium	2
							Weltevreden	1
7205	Serum agglutination	Shanghai	2016-2017	Swine	1035	147	Enteritidis	68
							Typhimurium	48
							Derby	10
							Newport	6
							Agona	4
							Choleraesuis	4
							Senftenberg	3
							Aberdeen	2
							Thompson	2
7207	Serum agglutination	Gansu	2015-2018	Swine	225	7	Typhimurium	1
							Enteritidis	1
							Derby	2
							Agona	1
							Senftenberg	2
		Gansu	2015-2018	Ruminant	450	12	Typhimurium	1
							Enteritidis	3
							Derby	4
							Anatum	2
							London	2
		Gansu	2015-2018	Poultry	308	15	Typhimurium	2
							Enteritidis	3
							Derby	2
							Anatum	3
							Kentucky	2
							Senftenberg	1

							Heidelberg	2
		Gansu	2015-2018	Food	1199	9	Enteritidis	1
							Derby	4
							Anatum	2
							Senftenberg	2
7210	Serum agglutination	Henan	2017	Swine	1732	337	Agama	1
							Agona	2
							Derby	131
							Newlands	14
							Rissen	51
							Typhimurium	79
							Bovismorbificans	11
							Emek	3
							London	33
							Chester	2
							Others	10

Table S4

No.	AMR test methods	Province	Sampling time	Host	Sample size	Numb	Number of resistant isolates																				
							Aminoglycoside			Cephem							Penicillin		Quinolone		β-lactam	Phenicol	Tetracycline	Macrolide	Carbapenem	Polymyxin	Sulfonamides
							GEN	KAN	STR	FOX	CRO	CZ	CTX	CEP	CAZ	FEP	AMP	PEN	CIP	NAL	AMC	CHL	TET	AZM	IPM	COL	SXT
40	KB	Xinjiang	2017	Cats	307	99	0	0									0		3		4		8				
45	KB	Hunan	2019-2020	Swine	302	17	13		0			0			0				1		8						
65	dilution	Shanghai	2018-2019	Human	1810	155			131				10		7	7	91		100	69	68	43	70	14	1		41
98	KB	Liaoning	2020	Poultry	390	49	45	13										42			18		44				
110	Microbroth dilution	Henan	2018-2019	Swine	825	241	120		0		114		116		117		205		4				175			114	
111	KB	Shanxi	2014-2019	Human	9209	517							231		138		433		53	119							
114	KB, AIS	Guangdong	2019-2020	Human	617	39				39	6		2		2	2					12				0		4
126	Microbroth dilution	Shaanxi	2018-2020	Human	1435	106					23		32		41	26	94		96						1		42
165	Microbroth dilution	Jiangsu	2019	Human	1246	35	2			1	2	34	2				22		0	23		6	13	2			6
179	dilution	Beijing	2013-2018	Human	2076	113			39								45			63	29		39				
200	AIS	Beijing	2014-2019	Human	1870	84	10			7		15	15		7		42		30	50		18	37		6		30
202	KB	Chongqing	2020	Dogs	686	26	2	3	16	2	0	6	4		2	0	22		1	8		10	17		2		14
		Chongqing	2020	Cats	326	9	0	2	1	1	2	3	2		1	0	7		1	3		4	4		4		4
		Chongqing	2020	Cold-blooded	6	6	1	2	1	0	1	0	2		3	1	2		1	0		3	3		2		2
246	KB	Tianjin	2018-2020	Human	607	74	4			1		29	3		0		53		5	47		9	32	2	0		9
259	KB	Yunnan	2017-2020	Human	29674	151					63				55	60	132		42		34				0		56
271	KB	Hebei	2019-2020	Poultry	588	150	60	64	143		20						141	146	55				66			36	53
292	NA	Jiangxi	2016-2018	Human	5339	170											125						110				
303	AIS	Xinjiang	2019	Food	330	11	11					11					4								0		3
304	Microbroth dilution	Jiangsu	2015-2017	Human	389	15	2			1		8	3		2		9		10	8		5	7				
306	KB	Jiangsu	2018-2019	Poultry	360	110							0					110							0	0	
317	NA	Guizhou	2019	Human	347	36	15				15				11	10	25		12						1		
348	KB	Jilin	2019	Poultry	1024	148	78				18						123		26		142					83	
356	KB	Jiangsu	2019	Human	324	85	18				3	4	3	13			68		13	37	15		57		0		
381	NA	Liaoning	2019	Food	1786	54	9			6		18	6		6		21		48	18		9	21	3	3		
		Liaoning	2019	Human	600	55	9			3		25	9		2		29		35	26		13	26	1	6		
397	KB	Hubei	2015-2017	Human	1216	71	31				13	58			14	14	43		13								39
400	Microbroth dilution	Sichuan	2012-2019	Human	1829	160	40			14		124	43		33		134		104	77		63	123	14	6		58
417	KB	Jiangsu	2016-2018	Poultry	329	49		15			0		31				0		20		7						
423	KB	Shanghai	2016-2018	Human	414	48							21										21				14
426	KB	Tibet	2019	Swine	300	33	11		11	11		16	0					27				32	25	26			25
427	KB	Anhui	2010-2018	Human	2998	265	31	27	238			184	19		15	23	215		173	158		50	138	27	4		50
434	KB	Henan	2018-2019	Human	744	21	3					0	10			1	21		12						0		
442	dilution	Jiangsu	2019	Human	3582	105	15			10		73	13		11		72		105	56		36	54	8	3		35
446	KB	Jiangsu	2014-2018	Human	1943	249	24				18	25	24				125		3		4				0		74
447	KB	Guangdong	2016-2018	Poultry	435	17	0		2				2		2		16			17		2	6		0	17	2
457	KB	Xinjiang	2017-2019	Human	1200	641	320			0		19	37			14	347		0		0	16	5		9		11
474	KB	Xinjiang	2016	Poultry	400	110											19		13		17					0	
		Xinjiang	2016	Ruminants	97	63											32				16		29			0	
		Xinjiang	2016	Swine	400	40											20						22			0	
		Xinjiang	2016	Ruminants	250	17																				0	
477	KB	Jiangsu	2012-2019	Human	9096	563					129						445		20						1		230
478	dilution	Hubei	2018	Swine	640	39		21									37		15			23					
503	KB	Guangdong	2017-2019	Human	2312	343							89		34		241		209						2		137
515	dilution	Guangxi	2018	Human	8702	286	28			27		176	49		46		197		25	150		74	152	41	2		63
563	KB	Zhejiang	2015-2017	Human	932	208	23				17	18		19			106		2		3				0		48
577	KB	Guangdong	2015-2017	Human	12890	532					99				40		420		86						0		420
600	NA	Xinjiang	2018-2019	Ruminants	841	44			6	5	4	12	5	16	4	3	17										
657	dilution	Guangxi	2018	Human	401	29	2			0		5	6		4		25		0			20	22	0			8
684	KB	Zhejiang	2014-2018	Human	2327	299	259		219		251	294	251	242			139		290	242	277	185	157		291		
704	AIS	Hainan	2016-2018	Human	6837	766					132				80	83	455		175			328			3		321
705	NA	Shaanxi	2016-2018	Human	1329	28	6		21				4		2	4	11		2	12		7	4				
726	Microbroth dilution	Beijing	2018	Human	380	23	3		10	1		4	2		1	2	10		1	17		5	6	3	1	5	6
764	Microbroth dilution	Yunnan	2016-2018	Human	60533	186	34						18		0		113		14	66	37	18	99	4	0		108
766	Microbroth dilution	Zhejiang	2018	Poultry	596	78	13							35	17		64						65			2	35
794	KB	Zhejiang	2013	Human	517	31	2		21					3			20				0	4	15			2	5
798	KB	Sichuan	2012-2017	Human	5934	340							41				285		41								138
800	NA	Guangdong	2016-2017	Human	905	120	27			4		78	34		19		98		87	72		40	101		0		38
834	KB	Jiangsu	2015-2018	Poultry	5889	56	6			2		48	9				18	51				0	46				56
854	KB	Chongqing	2018	Poultry	350	8	5		8			8	2				8		1			8	8				8
863	Microbroth dilution	Henan	2018	Swine	840	45	26								3		33						37			1	18
882	KB	Guangxi	2015-2017	Poultry	1554	55	4		41				55					55			55	6	26			54	53
887	dilution	Zhejiang	2017-2018	Food	2039	37	4	8				27	0		0	0	17		22	18	10	25	22	0	0		9
909	KB	Beijing	2015-2018	Human	1847	37					4						26		14								2
924	KB	Guangxi	2018	Human	1238	63	48				24	16			20	17	31		3		4	2	16		0		1
931	AIS	Guangdong	2017	Human	10487	422					149				82	67	347								0		169
946	dilution	Jiangsu	2016-2017	Human	982	32	4			3		8	5		3		22		14	9		16	21	3			
961	dilution	Beijing	2017	Human	515	18														14				0			
997	dilution	Shanxi	2015-2017	Human	752	17	0			1		1	0		0		7		0	7		0	2	1	0		2
1013	AIS	Zhejiang	2013-2017	Human	1664	78					60				62	62	61		59						78		49
1014	KB, AIS	Guangdong	2015-2017	Human	4019	108					27				20	9	66		14						0		22
1024	dilution	Shanxi	2015-2016	Human	2505	51	6			1		31	7		3		36		6	29		9	22	7	0		23
1032	AIS	Guangdong	2013	Human	995	98					17				12												
		Guangdong	2014	Human	999	122					32				21												
		Guangdong	2015	Human	1086	182					73				57												
1046	KB	Guangdong	2015-2017	Human	3757	271					51				48	35	219		48			110			0		110

1062	NA	Liaoning	2017	Poultry	2000	73	66										73				66		71			70	70
1074	KB	Sichuan	2016-2017	Human	36451	43	6						25		23		21		3	21	30	5	34		9		16
1079	KB	Henan	2011-2013	Food	4303	331	58		116				25		43	5	141		29	184		116	191				91
1081	KB	Henan	2017	Poultry	351	62	5	5			10	10			5		13		5		5	0	16		0	0	11
1098	KB	Inner Mongoli	2018	Ruminants	317	30				17			3			0	0										
1100	KB	Shandong	2016-2017	Human	2400	27	9					7	1				18		5								9
1106	KB	Guangdong	2013-2016	Human	5820	480	139						43		30	33	308		173						0		
1127	dilution	Beijing	2015	Human	371	14	2			0		3	2		0		5		3	13		2	3	2	0		0
		Beijing	2016	Human	361	17	3			1		7	4		0		8		2	9		2	6	2	1		4
1148	dilution	Hebei	2017	Food	570	7	0			1		4			0		3		4	1		4	4	0	2		3
1175	AIS	Jiangsu	2015-2016	Food	368	37	13			2			9		5		24		8	27		8	14				
1180	KB	Sichuan	2018	Poultry	343	99	24	23			27						42		29	91	6		43				34
1189	dilution	Shandong	2009-2016	Food	115	26	6	5			5		5			2		9	3		1	10	16				5
		Shandong	2009-2016	Poultry	227	83	32	26			47					21		63	38		9	43	47				39
1225	E-test	Shanghai	2016-2017	Human	400	22	4		22	1		3	6		3	1	18		18						0		6
1235	dilution	Henan	2015	Poultry	554	77	28	27	50				36			11		47	34	77	31	40	62				25
1237	dilution	Shanghai	2016	Swine	254	82	22		73	3							48		28	32		50	65	4			35
		Shanghai	2016	Poultry	306	75	7		38	4							35		15	50		7	16	5			11
1248	dilution	Jiangsu	2016	Human	417	5	0			0			0						0					0			
1262	dilution	Jiangsu	2010-2016	Food	719	53	18			8		31	11		10		35		24	34		11	19	8	1		12
1271	NA	Beijing	2016	Human	340	21	8					19	10		12		13		18			3	9	2	0		0
1287	KB	Shandong	2017	Human	665	21	21				3				2		11								0		0
1288	dilution	Guangxi	2016	Food	12120	70	5						4		2		21		7	8		23	32		0		12
		Guangxi	2016	Human	5400	234	27						36		20		166		10	67		96	164		0		62
1295	KB	Yunnan	2015	Human	10342	56	7						6	53			50			50	20						
1305	AIS	Sichuan	2017	Swine	600	49	47				8	14			6	0	49		17						1		48
1308	AIS	Guangdong	2014-2017	Human	4847	192					5		5		4	3	26		13								9
1311	dilution	Beijing	2012-2017	Human	16349	677	55		369	10	46					28	288		43	452	276	101	168	111	0		87
1324	Microbroth dilution	Jiangxi	2015-2016	Human	1260	30	1			5		5	1		2		14		1	6		8	16	1	2		2
1325	Microbroth dilution	Xinjiang	2017	Ruminants	753	63			34										9			59	47				
1333	KB	Guangdong	2015-2017	Human	1136	108	95			90			83	15	82	81			105		84				108		89
1339	KB	Henan	2014-2015	Poultry	695	263	37	26			30	34			9		107		10		6	4	94		0	0	90
1345	E-test	Guangdong	2015	Human	4620	61				4					0	0			6		13				0		17
1359	KB	Jiangsu	2017	Swine	1727	184	92	113	121			64	60				103		114		118	108	156			22	63
1387	KB	Xinjiang	2015	Poultry	950	80	42										54		67				45				
1405	AIS	Zhejiang	2014-2015	Human	417	16	16			7			0		0	16			1						0		8
1407	dilution	Shandong	2010-2015	Human	513	31	1		22	0	10					3	20		17	20	11	16	12	28	0		7
1428	AIS	Beijing	2015-2016	Human	3068	33					1					1	11		4						0		6
1456	KB	Chongqing	2013-2015	Human	2538	155	46		71						17	2	73			132		36					
1458	KB	Anhui	2012-2014	Dogs	746	17	3		4	0	2					2	6										5
		Anhui	2012-2014	Feed	404	10	2		3	0	2					2	2										3
1474	Microbroth dilution	Henan	2015-2016	Human	5720	221	44			60			90		87		177		67	157		93	133				
1507	dilution	Jiangsu	2014-2016	Food	4186	112	14			6		54	7		7		62		51	37		31	66	12	0		31
1533	NA	Shaanxi	2008-2014	Human	2113	34	16					13	18			2	11		17			2	3		13		17
1567	KB	Jiangxi	2015-2016	Human	600	19					6		5		3	2	14		1								10
1579	dilution	Heilongjiang	2016	Poultry	880	86	24			3							54		24	82		25	31	3	0		26
1587	dilution	Shanghai	2016	Poultry	500	28	15						14		8		10		10	9			12				
1648	KB	Shanghai	2010-2014	Human	2119	374					78						265		303			111					122
1664	KB	Jilin	2016-2019	Swine	450	17	11	10	16		4		10				13					17	17				12
1671	KB	Beijing	2015	Human	403	25							1		0	0	8		3	18					0		2
1704	NA	Guangdong	2014-2015	Human	555	61					12						55		4					46	0		43
1734	NA	Xinjiang	2015	Poultry	950	80	42										54		67				45				
1754	NA	Zhejiang	2014-2015	Human	417	16	0			0			0		0	0			1						0		7
1765	Microbroth dilution	Jiangsu	2015	Human	756	32									2		18		2	16							
1826	dilution	Henan	2015	Human	2824	79	19			19			28		23		49		23	58		29	40				26
		Henan	2016	Human	2896	142	25			41			62		64		128		44	99		64	93				60
		Chongqing	2015	Poultry	94	11	0	2	4		0		0	2			6		0			4	6		0		
		Chongqing	2015	Swine	168	35	10	0	10		0		0	2			25		0			10	25		0		
1920	KB	Liaoning	2013-2015	Human	1288	439	9			11		10	20		10		38	37	18		9				1		38
1986	KB	Guangdong	2014-2015	Human	2456	283	274				47	133			57	47	244		96			133			11		
2000	dilution	Jiangsu	2015	Food	385	32	11			3			7		7		25		5	26		5	9				8
2014	KB	Zhejiang	2011-2014	Human	14658	447	159				0	12	9		0		245		126	80		95	166		9		177
2065	KB	Jiangsu	2014-2015	Human	860	108	8			4	5	6		9			49		4		20				1		7
2078	KB	Hunan	2013-2015	Food	545	14	2			2			1				6		2	4		3	8				3
2091	KB	Jiangsu	2012-2014	Human	2678	87	76			73		82	25		20	7	60				14				1		30
2097	dilution	Beijing	2014	Human	308	19	2			0			1						12	12		9	8				3
2134	KB	Heilongjiang	2010-2015	Human	1754	26	6					25	20		8				7						2		26
2138	KB	Guangdong	2011-2013	Human	2893	227					28		26		30		165		75			95					203
2140	KB	Shanghai	2014	Human	1218	47	5			1		10	4			1	30		28		30		19		0		10
2149	KB	Zhejiang	2013-2014	Human	426	26	5	3				1	1		1		12	25	3			4	13				13
2161	Microbroth dilution	Sichuan	2014	Swine	600	27	7	4				25		23			24						1			26	1
2173	KB	Shanghai	2011-2014	Human	7626	360	22		284						23		136			209	30	51	101				52
2191	KB	Sichuan	2012-2014	Human	982	35							6		7	6	20		7	20		13	18				
2201	AIS	Fujian	2009-2014	Human	3287	156							26		20		96		44		32	45					28
2268	Microbroth dilution	Jiangsu	2015	Food	385	32	11			3			7		7		25		5	26		5	9				
2282	KB	Zhejiang	2011-2014	Human	14658	447	159				0	12	9		0		245		88	80		95	166		8		177
2333	KB	Zhejiang	2014-2015	Human	860	108	8			4	5	6	9				49		4		20				1		7
2346	AIS	Hunan	2013-2015	Food	545	14	2			2			1				6		2	4		3	8				1
2359	KB	Jiangsu	2012-2014	Human	2678	87	11			14		5	62		67	80	27				73				86		57

2365	dilution	Beijing	2014	Human	308	19	2			0			1						12	12		9	8				3
2402	KB	Heilongjiang	2010-2015	Human	1754	26	6					25	20		8		26		7						2		26
2406	AIS	Guangdong	2011-2013	Human	2893	227					28		26		30		165		75			95			18		
2408	KB	Shanghai	2014	Human	1218	47	26			1		10	22			47	30	30	24				34		47		34
2417	KB	Zhejiang	2013-2014	Human	426	26	5	3				1	1		1		12	25	3			4	13				13
2429	Microbroth dilution	Sichuan	2014-2015	Swine	600	27	20	23				2					24				24	25	26			1	26
2441	KB	Shanghai	2011-2014	Human	7626	360											136										
2445	KB	Fujian	1993-2015	Human	15074	474					2		3				72		7	110	42	64	144				63
2448	AIS	Guangdong	2014	Human	3745	79	17				52	18			52	52	52		52						52		52
2469	KB	Fujian	2009-2014	Human	3287	156							26		20		96		44		32	45					28
2508	KB	Shanghai	2011-2012	Human	544	20	1								1	1					1	3					2
2531	AIS	Zhejiang	2011-2013	Cold-blooded	800	47	47				1	47			1	1	18		2						0		8
2544	KB	Shanghai	2012	Human	2423	69					1		6		1	1	28		6								14
2560	NA	Zhejiang	2010-2013	Human	10917	614	488			465	230				181	180	537		18						0		227
2578	dilution	Beijing	2013-2014	Human	605	35	7						3						5	23		11	16				12
2603	dilution	Sichuan	2009-2010	Poultry	1815	39	9										36				37		19			0	20
		Sichuan	2011-2014	Poultry	3630	115	61										93				86		84			19	86
2605	KB	Zhejiang	2014	Food	6812	208	189				208	208	208		208		88		208	208		208	208		208		152
2623	KB	Zhejiang	2014	Swine	300	104	43	64		30							72		29				79				77
2627	KB	Guangxi	2012-2014	Food	384	92		15	20		0		0		0		39		1	13	35	41	65				39
2632	KB	Guangdong	2013	Human	9847	329	239		126				291		301	296	121		300	203		248	116				125
2665	KB	Guangxi	2013-2015	Poultry	310	34	2	4	29					16				34				1	21		0		8
2725	Microbroth dilution	Guangdong	2007-2010	Human	63687	386	34			2			66						6	329		38	119				22
2738	dilution	Sichuan	2009-2014	Swine	2660	151	106										143				140		144			142	136
2753	KB	Shanxi	2013-2014	Human	789	18				2			4						0	7			8				2
2756	KB	Zhejiang	2008-2015	Human	9256	369	59				0	0	1		0		205		6	0		65	66		8		56
2761	KB	Jilin	2013	Swine	540	18	12		5								12			14			5				10
2768	KB	Xinjiang	2014	Swine	550	58	29	52									44		56		53		58				
2795	dilution	Hunan	2010	Food	692	94	12	21			1		4			4	46		14	30	58	79	59				35
2815	KB	Hunan	2009-2011	Human	767	35	12		18				5		2	5	22		0	20		11	18				20
2817	KB	Zhejiang	2011-2013	Human	361	7		0		0	0	0	0	0	0	0	2										
2837	dilution	Shandong	2006-2007	Poultry	992	178	1	1									122			178		10	119			2	
2851	Microbroth dilution	Sichuan	2012	Poultry	588	57	5				8	34					27		25	26	28	28					57
		Sichuan	2012	Poultry	127	16	0				1	1					4		2	4	4	4					16
2863	dilution	Guangdong	2013	Human	1150	72	15			11			11		7	7			5		8				0		19
2893	NA	Heilongjiang	2012	Swine	600	35				7			28		31	27	35							12	0		
2906	KB	Shanghai	2012	Poultry	320	70	13		42				13			7	36		12	53		18	27		0		16
2912	NA	Shanghai	2013	Human	583	19	12										12		12	12							
2946	dilution	Shandong	2012	Poultry	2496	497	279	267			254		255			234	398	399	248	425	371	274	315				269
2965	KB	Guangdong	2007-2009	Human	760	53					9						30		6								21
2994	AIS	Guangdong	2009-2013	Human	6920	344					38				28	38	209		98						0		130
3001	AIS	Zhejiang	2004-2010	Human	1258	32	22								3		25		7						0		9
3033	Microbroth dilution	Henan	2013	Swine	1820	125	52										89						119			13	76
3037	AIS	Henan	2012	Food	344	6	6				2	6			0	0	3		1						0		2
3317	Microbroth dilution	Hunan	2012	Food	991	12	10			12			12		2				12	9		9	5				9
3462	KB	Hubei	2009	Human	1022	15					2						8		1						1		2
3534	KB	Shanghai	2010-2011	Human	11035	64	64						64	64			62		64	5			60				58
3593	KB	Jiangsu	2010-2011	Human	456	8	3						4				1		3	3							3
3679	KB	Hunan	2009-2010	Human	10072	82	82				37	82			29	33	82		62						0		
3688	KB	Shanghai	2010	Food	840	36	4		9	3	1						21		16			9	11				
3716	Microbroth dilution	Sichuan	2006-2008	Food	2559	76	7	9	48		9						40		6			16	35			46	17
3732	KB	Jiangsu	2006-2010	Food	533	33	2						0	3		1	14		2	16		11	15				13
3744	KB	Shanghai	2010	Human	1045	160					160		152				160		160		148	156		160			156
3763	KB	Henan	2006-2010	Human	2327	85							0		0					36		10	21				13
3774	AIS	Beijing	2010	Human	308	10				1			3		1	1	3		0	5		1					1
3787	AIS	Guangdong	2009-2010	Human	1665	72	70				26	72	21		16	17	60		34		58						44
3837	dilution	Hubei	2006	Human	3746	221	49	33							0	7	81		27	73		60	110				63
3867	KB	Jiangsu	2008-2010	Human	58382	258	26	43		1			1	38		2	147		30	156		31	155				146
3887	KB	Xinjiang	2010	Human	392	15	1			4		6	4	9	2	1	10		2		7	3			0		3
3918	Microbroth dilution	Guangxi	2006-2009	Human	1930	32							7		10		25										25
3938	KB	Anhui	2008-2009	Swine	360	22	19	20			0							20	19			21	21				18
3942	KB	Zhejiang	2008-2009	Food	357	31	2	1		0			0	5			19		1	13			17				13
3972	KB	Henan	2009-2010	Poultry	617	98					14	17	34				60	98	16	27						0	45
3975	KB	Shanghai	2009	Human	2936	32	4						0	5			6		0	32			30				29
4044	Microbroth dilution	Shanxi	2007-2008	Food	764	359	93	133	104	32	57						118		75	126	115	93	201				208
4047	KB	Zhejiang	2007-2009	Human	719	18	1			0		1	1		0		6	6	1		6				0		2
4067	KB	Zhejiang	2005-2007	Human	47132	67	9			5	1		1		0	0	17										
4091	KB	Shanghai	2008	Human	83032	175	12		35								33		8	74		19	133				25
4105	KB	Beijing	2008	Human	301	32					5						20		3			5					5
4121	AIS	Jiangsu	2010	Food	372	19	3			2	1		1		0	0	4		3								
4147	NA	Beijing	2008	Human	786	38	5		16				0		0	0	14		2	26		4	12				
4191	dilution	Zhejiang	2005-2007	Human	1143	47	47				8		13		8	12	37					26	31				17
4199	AIS	Shandong	2007-2009	Human	1100	20	2							2			4	20	2			2			0		4
4206	KB	Henan	2006-2007	Food	379	121	24	27	32				11		11		41		21	99		28	42				26
4210	KB	Zhejiang	2004-2007	Human	73601	217	44						2				38		8		11						37
4232	KB	Chongqing	2007	Human	835	79			37				5							23			43				5
4239	KB	Zhejiang	2003-2007	Human	2056	251	1				19		21						37								46
4254	KB	Beijing	2007	Human	313	34					3						17		5			8					9
4312	KB	Beijing	1998-2007	Human	859	89					44				12		38		49						22		47

4361	KB	Beijing	2007	Human	1600	76					2						15					4					10
4374	KB	Guangdong	2007	Human	1128	71	0		49				3		1	0	29		13	36		19	39				
4387	KB	Sichuan	2007	Human	338	18		7	6	0				8		0	8										
4415	KB	Guangdong	2004-2007	Human	3257	60	16					56	45		20		60		20						4		60
4437	KB	Henan	2006-2007	Poultry	1128	68	60		66					30					38			32	54	40			
4447	NA	Guangxi	2005	Food	682	73		0		0	0	2		4		0	60		1	24	4	10	43				6
4492	AIS	Fujian	2006	Human	51548	86	4			3	0	3		17	4	7			2						0		26
4535	KB	Shanghai	2006	Human	3603	196	7						1	45			23		8	82		20	118				20
4641	dilution	Hubei	2004	Human	4536	64					3		3		3	2	39		25	49			43				63
4787	AIS	Zhejiang	2001-2004	Food	1047	98	6				0	11			0		17		5		12				1		8
4833	KB	Hebei	2005	Food	387	81	7		37		2	3		2	2		13		9	32	4	12	16				40
4848	KB	Shanghai	2002-2003	Food	303	112	7	2	30				0				32		19			27	71			2	32
4875	KB	Ningxia	2001-2006	Human	1942	30	7					29	23		9		30		9						2		30
4907	KB	Henan	2006	Food	540	57	18		39								18		20	35		18	22				18
4914	KB	Jiangsu	2002-2004	Food	957	20	7			0			1	3	0	0			2		1				0		4
5370	KB	Zhejiang	2001	Human	12850	25	0	3	4		0	0			0		1		1			3	4			0	4
5375	KB	Jiangxi	2001	Human	15420	193																			183		158
5376	AIS	Fujian	2002-2004	Food	978	64	5					8					13										5
5384	KB	Guangdong	2000-2001	Human	50630	130	1	14	94								24	108				24	112				6
5414	KB	Hubei	1997-2000	Human	45353	558	0											558				544	476				0
5518	KB	Guangdong	1999-2000	Human	10083	27	3	5	3			0	0	0			27	27				0	10			6	
5664	KB	Fujian	1991-1994	Human	887	73	32					35						29				33	57			62	29
5827	KB	Beijing	1991-1995	Human	8126	167			6				7						12			4	6				
5878	NA	Tibet	1993	Human	626	11											2						7		7	4	
6598	KB	Taiwan	2005-2006	Poultry	3160	359					1						15		0	18	1	10	56		0	199	17
6609	KB	Taiwan	2005-2007	Dogs	1109	168	3				0						38		0	47	13	40	64			166	37
6685	KB	Hebei	2019-2020	Poultry	588	150	60	64	143		20						141	146	55				66			36	53
6733	Microbroth dilution	Taiwan	2014-2016	Poultry	2040	243	75		129			91							2	165	79	168				189	179
6788	KB	Taiwan	2008	Poultry	1121	110	0		0		0	0		0			2		0		0	5	3			1	2
6791	KB	Taiwan	2005	Swine	440	58	22		34		0								0	38	2	36	46				35
6806	KB	Taiwan	2002-2003	Poultry	1595	164			154								50		13			59	64				63
6837	KB	Taiwan	2003-2005	Dogs	928	40	2	2	14	1	0								0	17	20	21	31				15
6877	KB	Taiwan	2000-2001	Poultry	2000	91	14												0	4	26		47			5	32
7063	Microbroth dilution	Henan	2014-2015	Poultry	2139	45	30	35	36													0		0	0		
7080	Microbroth dilution	Shanghai	2018-2019	Swine	1389	239	97	129	69								219		41	114	143	185	215				191
7091	Microbroth dilution	Hubei	2019	Swine	896	155	37						40				86		26		67	155	100				155
7093	KB	Beijing	2017	Food	1234	341	24				24						79								0		
7098	KB	Shandong	2019-2020	Poultry	360	155											0										
7101	KB	Shandong	2015-2017	Poultry	1288	86			25								57			51		35	41				33
7110	AIS	Shandong	2013-2018	Poultry	923	280	6										122				0	2	2		0		40
7113	KB	Chongqing	2018-2019	Swine	724	92	0		34	10	12						56		6	0	0	47	79				
7127	KB	Shandong	2018	Poultry	600	67	10										46										
7145	Microbroth dilution	Shaanxi	2013-2014	Poultry	814	46											46		24	46	46						46
7173	Microbroth dilution	Guangdong	2017-2019	Poultry	365	151	47		68				25			0	14		29	81		94	129		0		127
7177	Microbroth dilution	Hubei	2016	Swine	1440	177	103										128		88		53	111	164				136
7185	KB	Jiangsu	2016-2017	Swine	459	80	13	12	23	0	0						74		8			37	65			0	
7194	Microbroth dilution	Hubei	2016-2018	Swine	4744	922	317										661		292		304	458	804				840
7201	KB	Guangdong	2017-2018	Poultry	1000	54	15	32	17								35	20	7	11	22		24	8			
7205	Microbroth dilution	Shanghai	2016-2017	Food	1035	147	27	27	68								63		37	73		32	38	0			90
7207	Microbroth dilution	Gansu	2015-2018	Food	2182	43													17	37							
7210	Microbroth dilution	Henan	2017	Swine	1732	337	150	134	233		9						275		337		271	238	335			4	265

Table S5

Region	Provinces and municipality
Northern China	Beijing
	Gansu
	Hebei
	Heilongjiang
	Henan
	Inner Mongolia
	Jilin
	Liaoning
	Ningxia
	Qinghai
	Shaanxi
	Shandong
	Shanxi
	Tianjin
	Xinjiang
Southern China	Anhui
	Chongqing
	Fujian
	Guangdong
	Guangxi
	Guizhou
	Hainan
	Hong Kong
	Hubei
	Hunan
	Jiangsu
	Jiangxi
	Shanghai
	Sichuan
	Taiwan
	Tibet
	Yunnan
	Zhejiang
	Macau

Table S6

Antimicrobial agent abbreviation		
**Drug class or subclass**	**Antimicrobial agent**	**Abbreviation**
	Gentamicin	GEN
	Kanamycin	KAN
Aminoglycoside	Streptomycin	STR
	Cefoxitin	FOX
	Ceftriaxone	CRO
	Cefazolin	CZ
	Cefotaxime	CTX
	Cephalothin	CEP
	Ceftazidime	CAZ
Cephem	Cefepime	FEP
	Penicillin	PEN
Penicillin	Ampicillin	AMP
	Ciprofloxacin	CIP
Quinolone	Nalidixic acid	NAL
β-lactam	Amoxicillin/Clavulanate	AMC
Phenicol	Chloramphenicol	CHL
Tetracycline	Tetracycline	TET
Macrolide	Azithromycin	AZM
Carbapenem	Imipenem	IPM
Polymyxin	Colistin	COL
Sulfonamides	Trimethoprim-Sulfamethoxazole	SXT

Other Abbreviations	
**Full name**	**Abbreviations**
Food animal	FA
Food	F
Human	H
Poultry	P
Swine	S
Ruminant	R
Northern China	N
Southern China	S
China	C
Polymerase Chain Reaction	PCR
Pulse Field Gel Electrophoresis	PFGE
Whole Genome Sequencing	WGS
AIS	Automated identification system
KB	Kirby-Bauer
